# TRIM37 interacts with TRIM28 to maintain primordial germ cell identity during migration

**DOI:** 10.1038/s41422-026-01272-2

**Published:** 2026-07-30

**Authors:** Qing Li, Xidi Yin, Chuanyin Li, Li Guo, Yingyi Wang, Yanbo Cheng, Nan Liu, Qi Yin, Yifu Ding, Meng Yan, Zihan Liu, Junyu Chen, Jin Zhang, Shuo Huang, Wangpeng Gu, Anjing Guo, Huanhuan Deng, Yuanyuan Li, Di Chen, Ian Chambers, Hui Zheng, Ronggui Hu, Man Zhang, Jinsong Li

**Affiliations:** 1 Key Laboratory of Multi-Cell Systems, Shanghai Key Laboratory of Molecular Andrology, Center for Excellence in Molecular Cell Science, Shanghai Institute of Biochemistry and Cell Biology, University of Chinese Academy of Sciences, Chinese Academy of Sciences, Shanghai, China; 2 Department of Histoembryology, Genetics and Developmental Biology, Shanghai Key Laboratory of Reproductive Medicine, Shanghai Jiao Tong University School of Medicine, Shanghai, China; 3 Institute for Clinical Research, Shanghai General Hospital, Shanghai Jiao Tong University School of Medicine, Shanghai, China; 4 Department of Colorectal Surgery and Oncology (Key Laboratory of Cancer Prevention and Intervention, China National Ministry of Education, Key Laboratory of Molecular Biology in Medical Sciences, Zhejiang Province, China), the Second Affiliated Hospital, Zhejiang University School of Medicine, Hangzhou, Zhejiang China; 5 School of Life Science and Technology, ShanghaiTech University, Shanghai, China; 6 GMU-GIBH Joint School of Life Sciences, the Fifth Affiliated Hospital of Guangzhou Medical University, Guangzhou Medical University, Guangzhou, Guangdong China; 7 Guangzhou National Laboratory, Guangzhou, Guangdong China; 8 Center for Regeneration and Cell Therapy of Zhejiang University, Center for Reproductive Medicine of The Second Affiliated Hospital, University of Edinburgh Institute (ZJU-UoE Institute), Zhejiang University School of Medicine, Zhejiang University, Hangzhou, Zhejiang China; 9 Institute for Stem Cell Research, School of Biological Sciences, University of Edinburgh, Edinburgh, UK; 10 Liangzhu Laboratory, MOE Frontier Science Center for Brain Science and Brain-Machine Integration, State Key Laboratory of Brain-Machine Intelligence, Zhejiang University, Hangzhou, Zhejiang China

**Keywords:** Embryonic germ cells, Embryonic germ cells

## Abstract

During primordial germ cell (PGC) specification, repression of somatic programs is essential for the establishment of germline identity. However, mechanisms that safeguard PGC fate thereafter remain unknown. Here, we identify the E3 ubiquitin ligase TRIM37 as a critical safeguard of PGC fate during migration. *Trim37* deficiency causes severe PGC defects beginning at embryonic day 9.5 (E9.5) with complete PGC depletion by E12.5, and leads to an aberrant transition toward somatic cell states. Mechanistically, TRIM37 binds TRIM28 through its MATH domain and ubiquitinates TRIM28 via its RING domain, enhancing the TRIM37–TRIM28 interaction and promoting the nuclear retention of TRIM37. Forced nuclear export of TRIM37 results in PGC loss. Moreover, disruption of TRIM37 ligase activity or mutation of TRIM28 ubiquitination sites compromises PGC maintenance. We further show that the TRIM37–TRIM28 complex, likely acting in cooperation with AP2γ, restricts chromatin accessibility and H3K27ac levels at somatic gene loci, thereby repressing somatic transcriptional programs in PGCs. Together, our findings uncover a TRIM37–TRIM28–AP2γ regulatory complex that safeguards germ cell fate by preventing the activation of somatic transcriptional programs during PGC migration.

## Introduction

The germline and soma are the two essential cell types within an individual embryo proper. During embryonic development, primordial germ cells (PGCs), the precursors of gametes, are vital for gametogenesis and species continuity.^1,2^ In mice, PGCs form from pluripotent epiblast cells at ~E6.25 and are induced by bone morphogenetic protein (BMP) signaling from extraembryonic tissues.^3–6^ BMP, together with WNT signaling, downregulates OTX2 and upregulates germline-specific factors to segregate germ cells from somatic cells (SCs).^3–5,7^ Three master transcription factors, BLIMP1 (also known as PRDM1), PRDM14, and AP2γ (also known as TFAP2C), are essential for mouse PGC specification^8–10^ and coordinately downregulate somatic genes while activating germline-specific and pluripotency-associated regulators.^11,12^ Following specification, PGCs proliferate and migrate through the dorsal mesentery to the genital ridges.^3,13,14^ During migration, extensive epigenetic reprogramming occurs, including genome-wide DNA demethylation and alterations in histone modifications, which prepare the epigenome for meiosis and eventual fertilization.^1–3,15^ However, the mechanisms that maintain PGC identity during migration and epigenetic reprogramming remain largely unknown.

Here, we identify TRIM37, a member of the TRIM E3 ubiquitin ligase family,^16^ as indispensable for maintaining PGC identity during migration. TRIM37 consists of a Really Interesting New Gene (RING) domain, B-box motifs, a coiled-coil region, and a meprin and TRAF homology (MATH) domain at its C-terminus.^16^ *TRIM37* mutation causes Mulibrey (muscle-liver-brain-eye) nanism in humans, a rare autosomal recessive disorder characterized by severe growth failure due to defects in mesoderm-derived tissues, as well as infertility.^16–18^ We show that TRIM37 interacts with TRIM28, a corepressor of Krüppel-associated box zinc finger proteins (KRAB-ZFPs).^19^ The interaction between TRIM37 and TRIM28 represses somatic gene expression and maintains PGC fate. TRIM37 ubiquitinates TRIM28 at lysine residues K774 and K779, strengthening the interaction between TRIM37 and the TRIM28–NuRD repressor complex at somatic cell-specific genomic loci. Our results show that repression of somatic developmental genes by the TRIM37–TRIM28 complex is essential for maintaining PGC fate.

## Results

### Genetic screening in mice identifies *Trim37* that is essential for PGC development

We previously described a system to screen for functional genes in mice.^20,21^ In this approach, androgenetic haploid embryonic stem cells (AG-haESCs) with deletions in the differentially methylated regions of the imprinted loci *H19*-DMR and *IG*-DMR (termed DKO-AG-haESCs) are injected into the oocyte cytoplasm to efficiently produce gene-modified animals, referred to as semi-cloned mice.^20^ Combining DKO-AG-haESCs with an sgRNA library allows mice with specific gene mutations to be produced in a single step.^21^ However, the overall efficiency of the method remained suboptimal. Recently, through combined application with the CRISPR-Cas9-based base editor, this approach allowed us to characterize individual residues critical for the function of DND1 that is an essential gene in PGC development,^22^ in which, a CAG promoter showed to have a high mutagenesis efficiency in vivo.^23^ Therefore, we first aimed to improve and optimize the approach for functional gene screen by replacing the EFS promoter^21^ with the CAG promoter in the DKO-AG-haESC line carrying an *Oct4-EGFP* transgene (Supplementary information, Fig. S1a). We generated cell lines referred to as EFS-C9 and CAG-C9. Comparison of the CAG-C9 and EFS-C9 lines showed that the CAG-C9 lines had higher expression of Cas9 mRNA and protein (compare Supplementary information, Fig. S1b, c with S1g, h) and superior GFP mutagenesis efficiency (Supplementary information, Fig. S1d−f, i−k). We selected the CAG-C9-3 line for subsequent experiments. As expected, the three designed sgRNAs efficiently induced indels in *Dnd1* in CAG-C9-3 cells (Supplementary information, Fig. S2a, b). Next, the ability of these cells to mutate *Dnd1* in vivo was tested by injecting CAG-C9-3 cells infected with a lentivirus carrying *Dnd1* sgRNA1 into oocytes to produce semi-cloned embryos (Supplementary information, Fig. S2c). Encouragingly, 13 of 15 semi-cloned embryos derived from CAG-C9-3 cells showed biallelic mutations in *Dnd1*, leading to PGC developmental failure at E12.5 (Supplementary information, Fig. S2d).

**Fig. 1:**
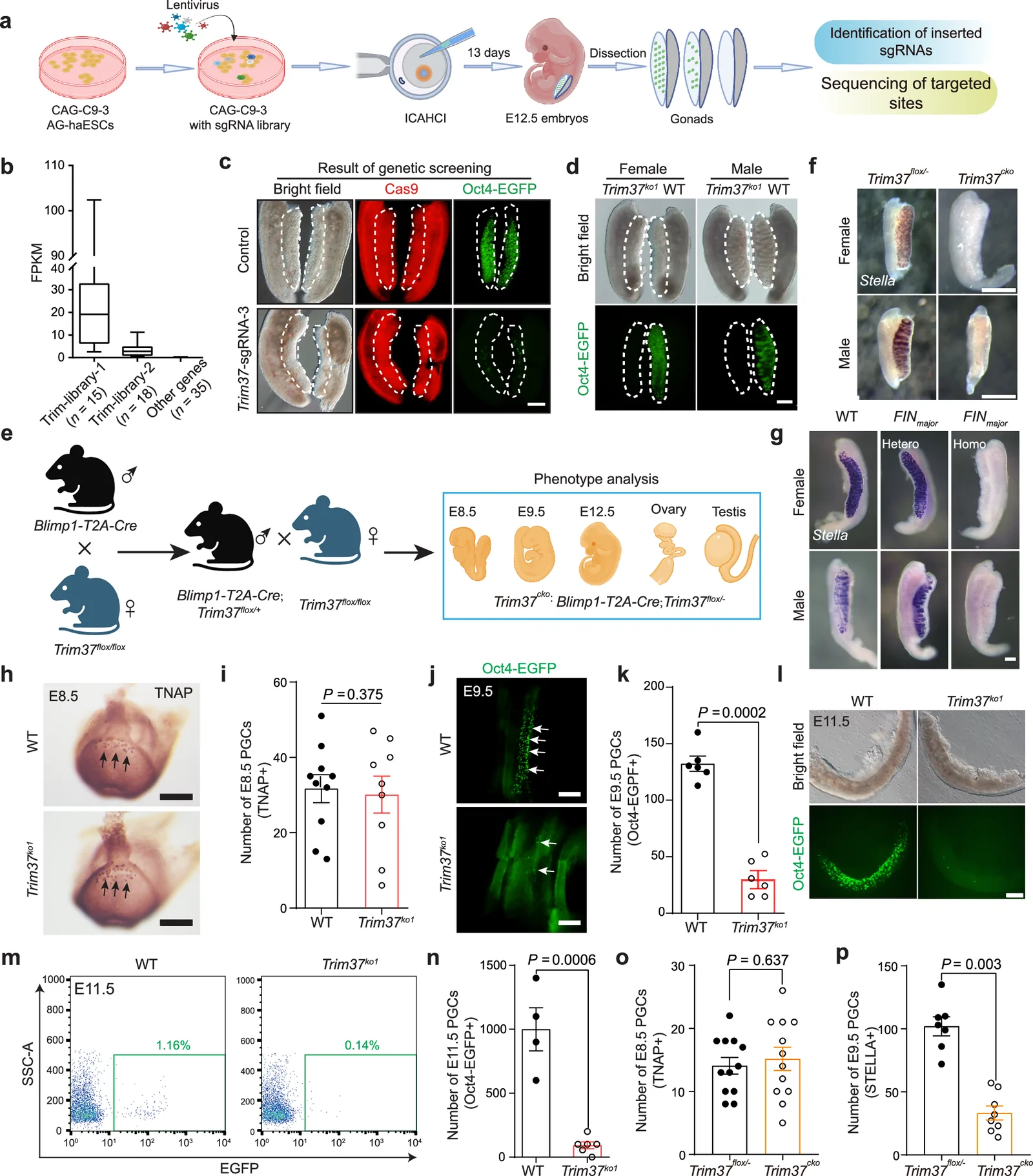
*Trim37* is required for PGC maintenance at E9.5. **a** Schematic of in vivo screening for key genes involved in PGC development using semi-cloning technology with CAG-C9-3 AG-haESCs carrying an sgRNA library. ICAHCI, intracytoplasmic AG-haESC injection. **b** Expression levels of TRIM family genes in E9.5 PGCs determined by RNA-seq. According to FPKM values, TRIM family genes were divided into three groups (Supplementary information, Table S1). **c** Representative images illustrating the screening results, in which rare PGCs (green signal) were observed in the gonads of E12.5 embryos carrying *Trim37*-sgRNA-3 (*n* = 2). This screening phenotype was subsequently validated using independent *Trim37* knockout and conditional knockout mouse models. The middle panel shows gonads under fluorescence imaging, with Cas9 expression indicated by red fluorescence. Scale bar, 200 µm. **d** Phenotype analysis of gonads in E12.5 embryos (wild-type and *Trim37*^*ko1*^). Green fluorescence indicates Oct4-EGFP expression and shows the locations of PGCs. Scale bar, 200 μm. **e** Strategy for generating *Trim37*^*cko*^ embryos and mice using *Blimp1-T2A-Cre* and *Trim37*^*flox/flox*^ parents. **f** Analysis of PGCs in E12.5 *Trim37*^*flox/–*^ and *Trim37*^*cko*^ gonads by in situ hybridization (ISH) using a *Stella* probe, showing no PGCs in *Trim37*^*cko*^ gonads. Scale bars, 500 μm. **g** Analysis of PGCs in E12.5 wild-type, *FIN*_*major*_ heterozygous, and homozygous gonads by ISH using a *Stella* probe, showing no PGCs in homozygous mutant gonads. Scale bar, 100 μm. **h** Analysis of PGC numbers in E8.5 wild-type and *Trim37*^*ko1*^ embryos by alkaline phosphatase staining. Arrows indicate TNAP-positive PGCs. Scale bars, 200 μm. **i** Boxplots showing the number of TNAP-positive PGCs in E8.5 embryos (wild-type and *Trim37*^*ko1*^). **j** Whole-mount immunofluorescence staining for Oct4-EGFP in E9.5 wild-type and *Trim37*^*ko1*^ embryos. Arrows indicate Oct4-EGFP-positive PGCs. Scale bars, 200 μm. **k** Boxplots showing the number of PGCs with Oct4-EGFP signals in E9.5 embryos (wild-type and *Trim37*^*ko1*^). **l** Representative images of gonads in E11.5 embryos (wild-type and *Trim37*^*ko1*^). Scale bars, 200 μm. **m** Representative FACS results showing Oct4-EGFP signals in PGCs from E11.5 wild-type and *Trim37*^*ko1*^ gonads. **n** Boxplots showing the number of PGCs with high EGFP intensity in E11.5 gonads (*n* = 4 embryos for wild-type and *n* = 6 embryos for *Trim37*^*ko1*^). **o** Boxplots showing the number of TNAP-positive PGCs in E8.5 embryos (*Trim37*^*flox/–*^ and *Trim37*^*cko*^). **p** Boxplots showing the number of PGCs with STELLA signals in E9.5 embryos (*Trim37*^*flox/–*^ and *Trim37*^*cko*^). Data are presented as mean ± SEM, with each circle representing an individual embryo. *P*-values were determined using an unpaired two-sided Student’s *t*-test in **i**, **k**, and **n**–**p**. Four embryos were analyzed for each group in **d**, **f**, and **g**.

Using the CAG-C9-3 cell-mediated in vivo mutagenesis system, we performed genetic screening for genes crucial for PGC development using sgRNA libraries targeting different proteins (Fig. 1a). In one library, we targeted the TRIM gene family, one of the largest E3 ubiquitin ligase families.^24^ TRIM proteins possess a tripartite motif (TRIM) composed of a RING domain, one or two B-box domains, and a coiled-coil region (RBCC)^24,25^ and may contain additional domains. TRIM proteins participate in various developmental processes, including innate immunity, carcinogenesis, and germ cell development, through ubiquitination-mediated protein degradation or stabilization.^25–27^ We first performed RNA-seq of wild-type PGCs at E9.5 and analyzed the FPKM values of TRIM family genes (Supplementary information, Table S1). Among the 68 members,^28^ 33 TRIM genes were expressed (average FPKM > 0.5) in PGCs and were selected for further analysis (Fig. 1b). These genes were divided into two groups containing 15 and 18 genes according to their transcript levels, and three sgRNAs per gene were designed for sgRNA library construction (Fig. 1b; Supplementary information, Table S2). After transfection of the sgRNA libraries into CAG-C9-3 cells, we obtained two stable cell populations carrying constitutively expressed Cas9 and sgRNAs (Trim-library-1 and Trim-library-2). Deep-sequencing analysis revealed that all sgRNAs were present in the cells (Supplementary information, Fig. S3a). We obtained E12.5 semi-cloned embryos using intracytoplasmic AG-haESC injection (ICAHCI) and dissected the gonads for phenotypic analysis (Fig. 1a). We chose embryos that exhibited impaired or failed PGC development for genotypic analysis. Genes targeted in at least two embryos with decreased PGCs were identified as potential candidates.

In our initial phenotype-driven screen, 238 semi-cloned embryos were produced from Trim-library-1 and Trim-library-2, among which two embryos exhibited PGC failure, and two others showed reduced PGC numbers (Supplementary information, Fig. S3b, c). To evaluate overall mutagenesis efficiency and library coverage, we genotyped 56 embryos, including all four phenotypic embryos and 52 embryos with apparently normal PGC development (Supplementary information, Fig. S3c and Table S3). Of these, 46 embryos carried biallelic mutations that collectively covered 24 *Trim* genes, indicating high editing efficiency and broad representation of the library in vivo (Supplementary information, Fig. S3c and Table S3). The two embryos with reduced PGC numbers were independently targeted at distinct genes, yet other embryos targeted at the same genes exhibited normal PGC numbers (Supplementary information, Table S3), suggesting potential off-target effects. In contrast, two independent embryos harboring biallelic *Trim37* mutations displayed severe PGC loss, fulfilling our predefined screening criteria for a positive hit and identifying *Trim37* as a positive candidate gene in this screen (Fig. 1c; Supplementary information, Fig. S3c). To validate its function in PGC development, we generated two independent *Trim37* knockout models (*Trim37*^*ko*1^, 7-bp insertion in exon 4; *Trim37*^*ko*2^, 1-bp deletion in exon 2) by zygote injection of CRISPR-Cas9 with different sgRNAs (Supplementary information, Fig. S3d, e). Both knockout models lacked detectable TRIM37 protein (Supplementary information, Fig. S3f, g). Notably, PGCs were completely absent in the E12.5 gonads of both models (Fig. 1d; Supplementary information, Fig. S3h). Together, these results confirm *Trim37* as an essential factor for mouse PGC development.

### TRIM37 protects PGC development in a predominantly PGC-intrinsic manner

*Trim37* was expressed in both PGCs and SCs during PGC development (Supplementary information, Fig. S4a, b). To determine whether *Trim37*-deletion-induced PGC depletion is germ cell autonomous, we first constructed a *Trim37* knockout embryonic stem cell line (*Trim37*-del) based on an E14-derived ESC line in which 3×Flag was knocked in at the C-terminus of *Trim37* (*Trim37-3*×*Flag*) (Supplementary information, Fig. S4c, d). We then induced *Trim37-3*×*Flag* and *Trim37*-del ESCs into PGC-like cells (PGCLCs) in vitro according to a published protocol.^7^ The results showed that *Trim37* deletion significantly decreased the number of PGCLCs on day 6 but not on day 4 (Supplementary information, Fig. S4e−i), suggesting that *Trim37* is crucial for PGC development rather than PGC specification.

Next, to further investigate the role of *Trim37* in PGCs in vivo, we first generated *Blimp1-T2A-Cre* knock-in mice and validated Cre-mediated loxP recombination in gonads using Rainbow mice^29^ (Supplementary information, Fig. S5a−c). We next produced a *Trim37*^*flox*^ mouse model using semi-cloning technology (Supplementary information, Fig. S5d). By crossing the two models, we generated PGC-specific *Trim37* knockout mice, which displayed normal growth (Fig. 1e; Supplementary information, Fig. S5d−g). However, these mice completely lacked germ cells in both testes and ovaries (Supplementary information, Fig. S5h, i). In situ hybridization (ISH) for *Stella* showed a complete absence of PGCs in E12.5 *Trim37*^*cko*^ embryos (Fig. 1f). These results demonstrate that PGC depletion in *Trim37* knockout mice is most likely PGC autonomous.

### *Trim37* is involved in PGC migration

Phenotypic analysis of *Trim37*^*ko*1^ and *Trim37*^*ko*2^showed that adult mice from both lines displayed no overt abnormalities in body weight or morphology (Supplementary information, Fig. S5j−m), failing to phenocopy the prenatal-onset growth failure observed in Mulibrey nanism (MUL) patients.^16,30^ Interestingly, however, their testes and ovaries were atrophic and completely devoid of germ cells (Supplementary information, Fig. S5n−q), effectively recapitulating the infertility phenotype associated with MUL.^31^ Previous studies have shown that Finnish MUL patients carrying the *FIN*_*major*_ mutation (c.493-2A>G) in *TRIM37* are infertile, as this mutation causes aberrant splicing at the next AG site, resulting in a truncated protein lacking 174 amino acids.^17,18^ We therefore created a homozygous *FIN*_*major*_ mouse model carrying this mutation (Supplementary information, Fig. S6a) and found normal growth but no testicular or ovarian germ cells in adults (Supplementary information, Fig. S6b−e). In addition, both male and female genital ridges lacked PGCs at E12.5 (Fig. 1g). These results suggest a conserved function of TRIM37 in germ cell development in both mice and humans.

To investigate the stage at which PGC development fails in *Trim37* knockout mice, embryos were assessed at different embryonic days. Alkaline phosphatase staining revealed comparable numbers of PGCs at the base of the allantois in wild-type and *Trim37*^*ko*1^ embryos at E8.5 (Fig. 1h, i). However, whole-mount EGFP immunostaining in *Trim37*^*ko*1^ embryos showed a 70% reduction in PGC numbers 1 day later at E9.5 (Fig. 1j, k). This number further declined to less than 10% at E11.5, as quantified by flow cytometry (Fig. 1l−n). Consistently, similar results were obtained in *Trim37*^*cko*^ embryos. At E8.5, comparable numbers of PGCs were found in control and *Trim37*^*cko*^ embryos (Fig. 1o; Supplementary information, Fig. S6f). However, by E9.5, PGC numbers were specifically decreased in *Trim37*^*cko*^ embryos (Fig. 1p; Supplementary information, Fig. S6g). These results establish that TRIM37 is not required for PGC specification but plays an essential role in PGC migration.

### *Trim37* maintains PGC fate by inhibiting somatic transition

**Fig. 2:**
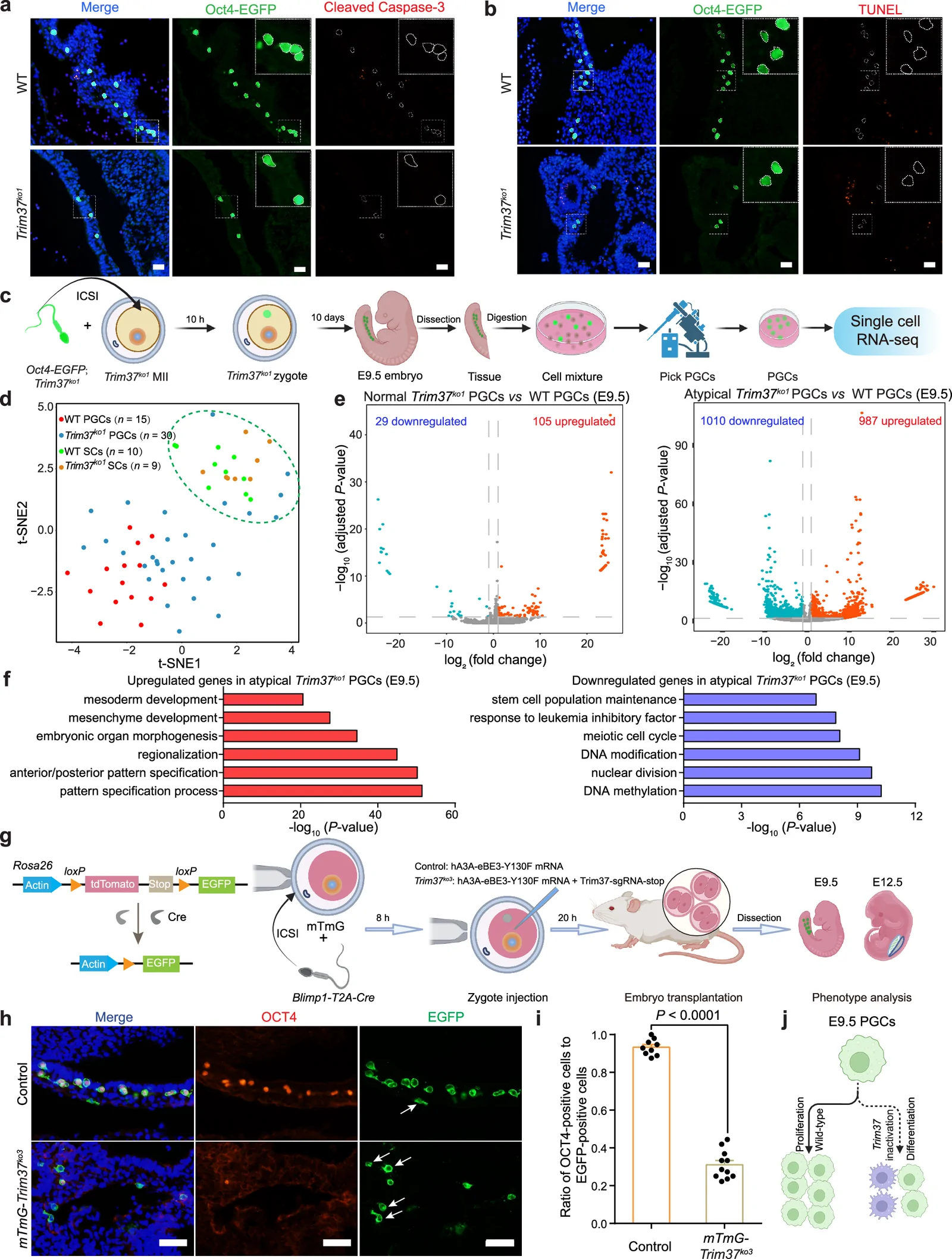
*Trim37* deficiency alters PGC fate during migration. **a**, **b** Immunofluorescence analysis of cleaved caspase-3 (**a**) and TUNEL staining (**b**) in E9.5 wild-type and *Trim37*^*ko1*^ embryos, focusing on Oct4-EGFP-positive PGCs. Cleaved caspase-3 and TUNEL are established markers of apoptosis. Signals were observed in positive control samples but were absent in both wild-type and *Trim37*^*ko1*^ PGCs. Four embryos were analyzed per group. Scale bars, 100 μm. **c** Schematic diagram of picking single PGCs from E9.5 embryos for scRNA-seq. **d** *t*-SNE analysis and cluster assignment for scRNA-seq data from wild-type and *Trim37*^*ko1*^ somatic cells (SCs) and PGCs at E9.5. *n* denotes the number of single cells analyzed per genotype, derived from three independent embryos. **e** Volcano plots showing differentially expressed genes between *Trim37*^*ko1*^ and wild-type PGCs (adjusted *P*-value < 0.05, FC > 2). *Trim37*^*ko1*^ PGCs were divided into two groups according to *t*-SNE analysis: normal *Trim37*^*ko1*^ PGCs, which were similar to wild-type PGCs, and atypical *Trim37*^*ko1*^ PGCs, which clustered with SCs. **f** Enriched gene ontology (GO) terms of significantly upregulated and downregulated genes in atypical *Trim37*^*ko1*^ PGCs compared with wild-type PGCs at E9.5. **g** Schematic diagram of the generation of the lineage-tracing system mediated by the *Blimp1-T2A-Cre* and *mTmG* mouse models. **h** Immunofluorescence analysis of E9.5 control and *mTmG-Trim37*^*ko3*^ embryos showing lineage tracing results, highlighting OCT4-positive PGCs (red), the lineage-tracing marker EGFP (green), and DAPI (blue). Arrows indicate GFP-positive non-PGCs. The *mTmG-Trim37*^*ko3*^ mutation was generated using a highly efficient cytosine base editor at the zygote stage. Scale bars, 50 μm. **i** Boxplots showing the ratios of PGCs with OCT4 signals relative to all EGFP-positive cells in control and *mTmG-Trim37*^*ko3*^ embryos. Data are presented as mean ± SEM, with each circle representing an individual embryo (*n* = 9 for control and *n* = 11 for *mTmG-Trim37*^*ko3*^). *P* < 0.0001; *P*-values were determined using an unpaired two-sided Student’s *t*-test. **j** Model illustrating how *Trim37* inactivation influences PGC fate at E9.5.

To investigate the fate of PGCs in *Trim37* knockout embryos, we first evaluated whether *Trim37* deficiency causes apoptosis in PGCs by cleaved caspase-3 staining and terminal deoxynucleotidyl transferase dUTP nick end labeling (TUNEL) assay. However, no apoptotic signal was observed in PGCs from *Trim37*^*ko*1^ embryos at E9.5 (Fig. 2a, b). To explore the mechanisms underlying PGC developmental failure, we carried out single-cell RNA sequencing (scRNA-seq) of wild-type and *Trim37*-deficient PGCs and SCs at E8.5 and E9.5 (Fig. 2c; Supplementary information, Fig. S7a). Because Oct4-EGFP does not reliably label early PGCs, we used the *Nanog-EGFP* transgenic model at E8.5 to distinguish PGCs from SCs based on EGFP expression^32^ (Supplementary information, Fig. S7a). To generate *Trim37* knockout PGCs in *Nanog-EGFP* transgenic embryos, we designed an sgRNA targeting exon 5 of *Trim37* (Supplementary information, Fig. S7b). This sgRNA effectively introduced a stop codon at the glutamine residue corresponding to amino acid 116 of TRIM37 (termed *Trim37*^*ko3*^) by intracytoplasmic co-injection with the hA3A-eBE3-Y130F base editor,^33^ leading to complete PGC deficiency at E12.5 (33 homozygotes out of 36 embryos) (Supplementary information, Fig. S7b−d). Interestingly, *t*-distributed stochastic neighbor embedding (*t*-SNE) analysis showed that *Trim37*^*ko3*^ PGCs clustered with their wild-type counterparts at E8.5 (Supplementary information, Fig. S7e−h). However, at E9.5, 20% (6 of 30) of *Trim37*-depleted PGCs (termed atypical *Trim37*^*ko1*^ PGCs) clustered with SCs (Fig. 2d; Supplementary information, Fig. S7i, j). Moreover, gene expression in atypical PGCs displayed characteristics of somatic development (Fig. 2e, f). We next integrated our scRNA-seq data with published E9.5 embryo datasets.^34^ While wild-type and normal *Trim37* knockout PGCs clustered with previously reported PGC populations, atypical E9.5 *Trim37* knockout PGCs instead grouped with mesoderm-associated lineages (Supplementary information, Fig. S8a). Consistently, pseudotime analysis showed that these cells deviated from the canonical PGC developmental trajectory (Supplementary information, Fig. S8b), indicating that PGCs lose their identity and begin to express somatic lineage genes.

To test the hypothesis that *Trim37* deficiency leads to loss of PGC fate, we established a lineage tracing system. *Blimp1-T2A-Cre* sperm were injected into *mTmG* oocytes to trace PGCs, followed by injection of a base editor mixture to introduce *Trim37*^*ko3*^ into the resulting embryos (Fig. 2g). In these embryos, PGCs switch from red fluorescence (membrane-targeted tdTomato, mT) to green fluorescence (membrane-targeted enhanced green fluorescent protein, mG) due to *Blimp1-T2A-Cre* expression^35^ (Fig. 2g). GFP was detectable in control gonads but not in *mTmG*-*Trim37*^*ko3*^ gonads at E12.5 (Supplementary information, Fig. S9a), suggesting that *Trim37* deficiency leads to the loss of PGCs prior to E12.5. To further investigate the timing of PGC loss, we collected control and *mTmG*-*Trim37*^*ko3*^ embryos at E9.5 and analyzed OCT4 protein (a PGC marker) and GFP (traced PGCs) signals (Fig. 2h). The results showed that the majority of GFP-positive cells (237 cells from 9 embryos) were PGCs in controls ( ~93.3%), whereas the proportion was ~32% in *mTmG*-*Trim37*^*ko3*^ embryos (185 cells from 11 embryos), with the remaining GFP^+^ population lacking OCT4 expression (Fig. 2h, i). These results indicate that *Trim37* is essential for maintaining PGC fate during migration and suggest that TRIM37 depletion may induce a switch from PGC to SC identity beginning at E9.5 (Fig. 2j).

### TRIM37 interacts with TRIM28 to maintain PGC fate

**Fig. 3:**
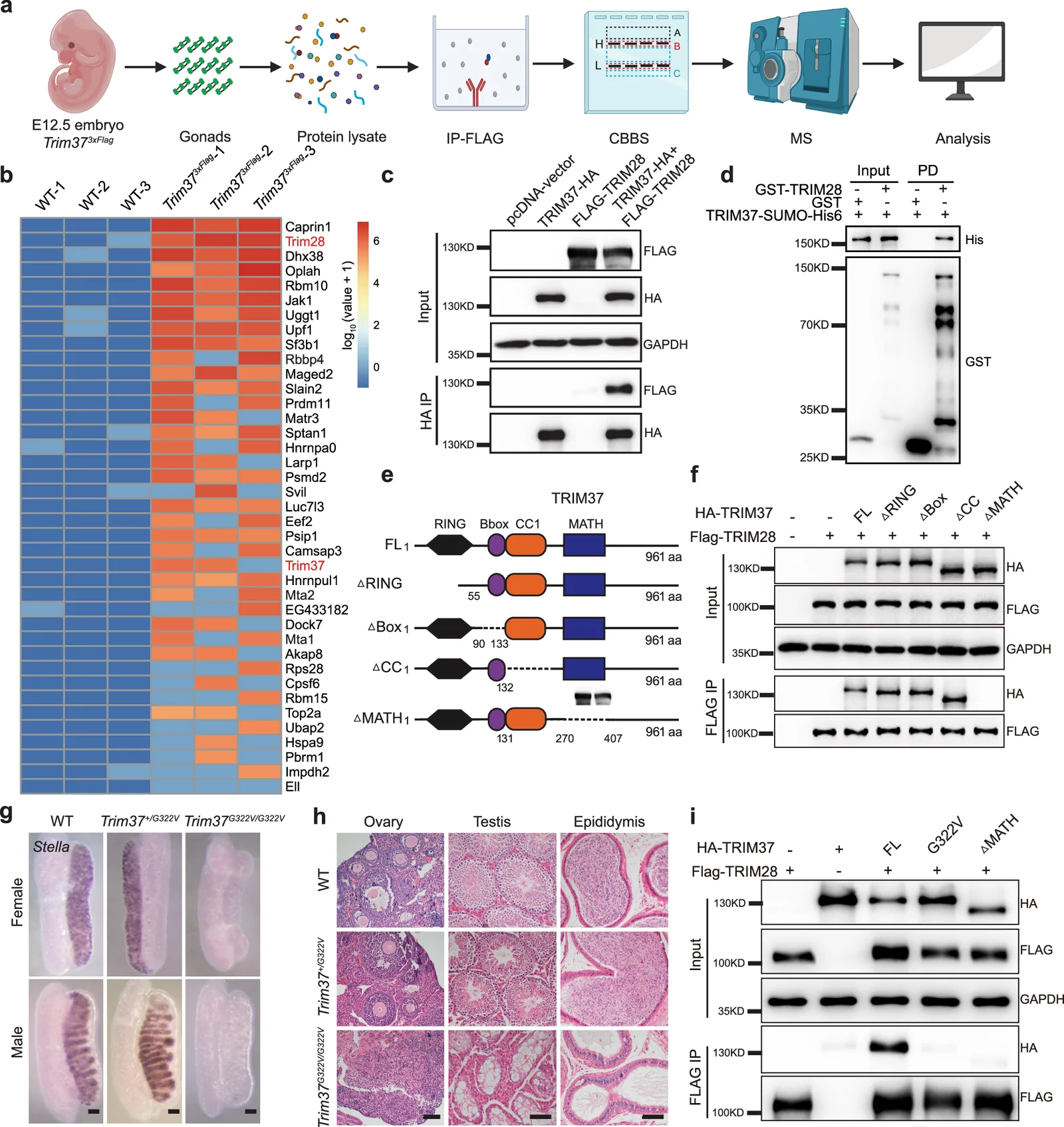
TRIM37 interacts with TRIM28 through its MATH domain to protect PGC development. **a** Diagram outlining the procedure for identifying TRIM37-interacting proteins by mass spectrometry (MS). Protein lysates were collected from E12.5 *Trim37*^*3×Flag*^ gonads. Immunoprecipitated proteins were divided into three groups (bands A, B, and C) according to band sizes following Coomassie brilliant blue staining (CBBS). **b** Heatmap of abundance values for proteins detected by MS analysis in wild-type and *Trim37*^*3×Flag*^ gonads. Deep blue squares indicate that no peptide was detected. **c** HEK293T cells expressing the indicated proteins were immunoprecipitated (IP) with anti-HA affinity gel and immunoblotted with anti-Flag, anti-HA, and anti-GAPDH antibodies. **d** GST pull-down assay showing that GST-tagged TRIM28 directly interacts with His6-tagged TRIM37. PD, GST pull-down. **e** Schematic illustration of full-length TRIM37 and its truncation mutants. FL, full-length; RING, Ring-finger domain; Bbox, B-box domain; CC, coiled-coil domain; MATH, meprin and TRAF–C homology domain. **f** Immunoblot analysis of co-immunoprecipitated lysates from HEK293T cells co-transfected with Flag-tagged TRIM28 and HA-tagged full-length TRIM37 or its truncation mutants. **g** Representative images of E12.5 wild-type, *Trim37*^*+/G322V*^, and *Trim37*^*G322V/G322V*^ gonads analyzed by ISH using a *Stella* probe, showing no PGCs in *Trim37*^*G322V/G322V*^ gonads. Five embryos were used for each group. Scale bars, 100 μm. **h** Histological analysis of ovaries, testes, and epididymides of wild-type, *Trim37*^*+/G322V*^, and *Trim37*^*G322V/G322V*^ mice showing no germ cells in the mutant organs. Three adult mice (8 weeks of age) were used for each group. Scale bars in all columns, 100 μm. **i** Immunoblot analysis of co-immunoprecipitated lysates from HEK293T cells co-transfected with Flag-tagged TRIM28 and HA-tagged full-length TRIM37 or its mutants (G322V and MATH deletion).

To further assess how TRIM37 maintains PGC identity, we examined gonadal extracts for TRIM37-interacting proteins by immunoprecipitation (IP) followed by mass spectrometric analysis (Fig. 3a). We first generated an AG-haESC line carrying 3×Flag at the N-terminus of TRIM37 (*Trim37*^*3×Flag*^ AG-haESCs) and produced a mouse model by injecting these cells into oocytes (Supplementary information, Fig. S9b). IP-mass spectrometry (MS) of *Trim37*^*3×Flag*^ and wild-type gonadal lysates identified 39 proteins (Fig. 3b; Supplementary information, Table S4). Among these candidates, TRIM28 ranked second and was prioritized because of its known role in heterochromatin maintenance and repression of differentiation genes,^36,37^ although its early embryonic lethality prevented its inclusion in the initial screening.^38,39^ To confirm the interaction between TRIM37 and TRIM28, co-IP assays were performed in HEK293T cells overexpressing tagged TRIM37 and TRIM28 proteins. The results revealed a strong interaction between TRIM37 and TRIM28 (Fig. 3c). Glutathione S-transferase (GST) pull-down assays using recombinant GST-TRIM28 and SUMO-TRIM37 proteins expressed in *E. coli* established that the interaction between TRIM37 and TRIM28 is direct (Fig. 3d).

To identify the functional domain of TRIM37 responsible for TRIM28 binding, we co-overexpressed TRIM28 with full-length or various truncated TRIM37 constructs in HEK293T cells (Fig. 3e). These analyzes showed that deletion of the MATH domain abolished TRIM37 binding to TRIM28 (Fig. 3f). Among TRIM family members, only TRIM37 contains a MATH domain, which facilitates protein–protein interactions.^16^ To investigate whether the TRIM37–TRIM28 interaction is required for PGC development, we constructed an AG-haESC line carrying a deletion of the MATH and injected it into *Trim37*^*ko1*^ oocytes (Supplementary information, Fig. S9c, d). The results showed that deletion of the TRIM37 MATH domain caused almost complete depletion of PGCs at E12.5 (Supplementary information, Fig. S9e).

A previous study reported that MUL patients carry a G322V mutation in the MATH domain.^40^ To determine whether mutation of G322 alone was sufficient to disrupt PGC development, we generated mice carrying the G322V mutation (*Trim37*^*G322V/G322V*^ model). Notably, these mice recapitulated the infertility phenotype observed in *Trim37*^*ko1*^ mice, exhibiting complete loss of PGCs and mature germ cells in both males and females (Fig. 3g, h). Co-IP showed that the G322V mutation dramatically decreased the interaction between TRIM37 and TRIM28 (Fig. 3i). These results establish that the TRIM37–TRIM28 interaction is essential for PGC development and suggest that the MUL patient-associated mutation causes infertility by disrupting the TRIM37–TRIM28 interaction.

**Fig. 4:**
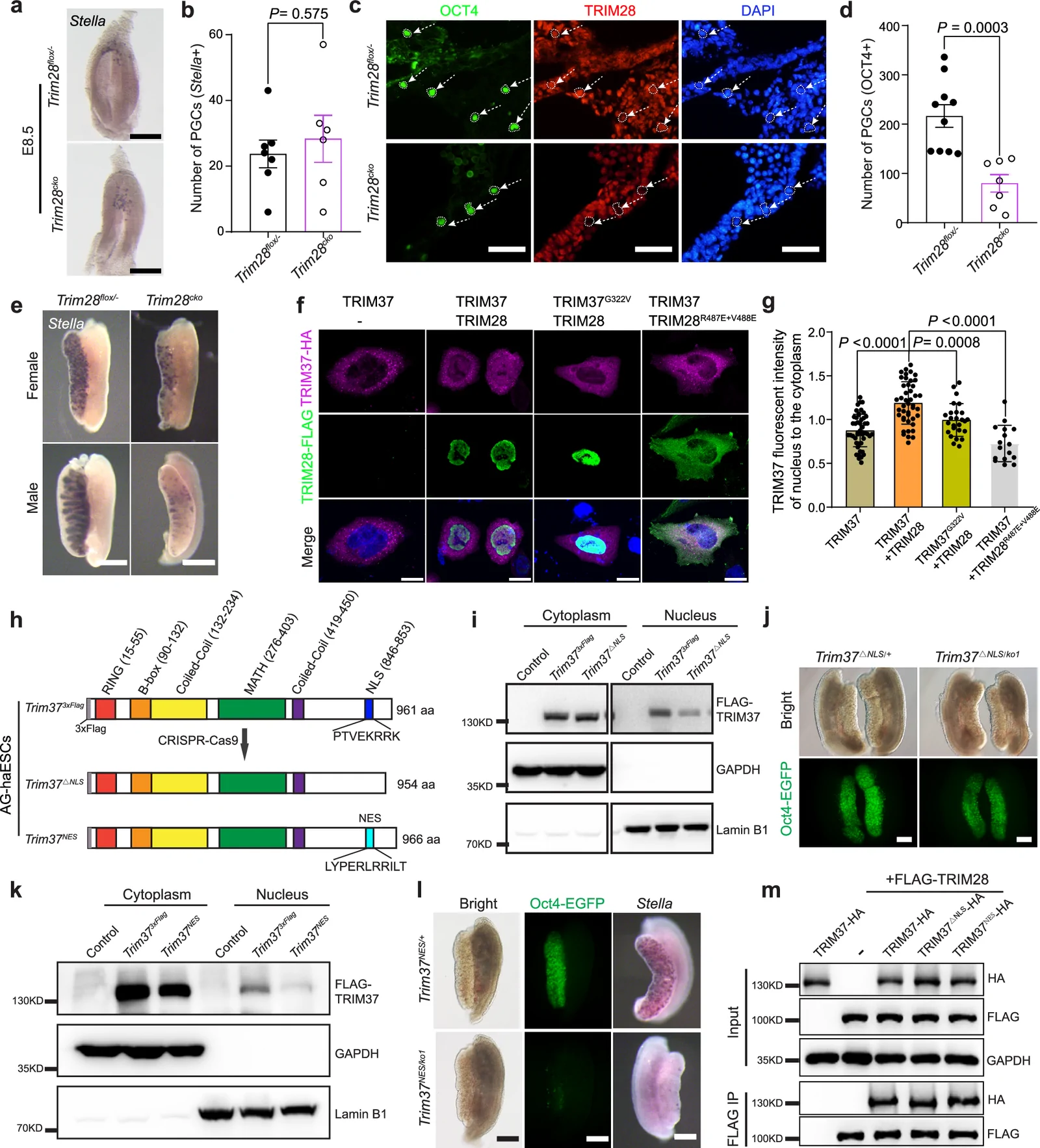
TRIM28-mediated nuclear localization of TRIM37 is essential for PGC development. **a** Representative images of E8.5 *Trim28*^*flox/–*^ and *Trim28*^*cko*^ embryos analyzed by ISH using a *Stella* probe. Scale bars, 200 μm. **b** Box plots showing the number of *Stella-*positive PGCs in E8.5 embryos (*n* = 7 for *Trim28*^*flox/–*^ and *n* = 6 for *Trim28*^*cko*^). **c** Immunofluorescence analysis of E9.5 *Trim28*^*flox/–*^ and *Trim28*^*cko*^ embryos for OCT4-positive PGCs (green), TRIM28 (red), and DAPI (blue). TRIM28 was specifically absent in PGCs from *Trim28*^*cko*^ embryos. Arrows indicate OCT4-positive PGCs. Six embryos were used for each group. Scale bars, 50 μm. **d** Box plots showing the number of PGCs with OCT4 signals in E9.5 embryos (*n* = 10 for *Trim28*^*flox/–*^ and *n* = 7 for *Trim28*^*cko*^). Data are presented as mean ± SEM, with each circle representing an individual embryo. **e** Representative images of E12.5 *Trim28*^*flox/–*^ and *Trim28*^*cko*^ gonads analyzed by ISH using a *Stella* probe, showing that PGC numbers were significantly decreased in female and male *Trim28*^*cko*^ gonads. Five embryos were used for each group. Scale bars, 100 μm. **f** Confocal microscopy of HeLa cells transfected with wild-type HA-tagged TRIM37 or the G322V mutant together with Flag-tagged wild-type TRIM28 or the R487E + V488E mutant. Nuclei were stained with Hoechst 33342 (blue). Scale bars, 10 μm. **g** Box plots showing the ratio of nuclear to cytoplasmic fluorescence intensity of wild-type TRIM37-HA and the G322V mutant corresponding to **f**. Data are presented as mean ± SEM. *P*-values were determined using an unpaired two-sided Student’s *t*-test in **b**, **d**, **g**. **h** Schematic illustration of generation of *Trim37*^Δ*NLS*^ and *Trim37*^*NES*^ AG-haESCs from *Trim37*^*3×Flag*^ AG-haESCs using CRISPR-Cas9 technology. The NLS sequence of TRIM37 was predicted in a previous study.^42^ **i** Immunoblot analysis showing expression levels of TRIM37 protein in the cytoplasm and nucleus of *Trim37*^*3×Flag*^ and *Trim37*^Δ*NLS*^ AG-haESCs using anti-Flag, anti-GAPDH, and anti-Lamin B1 antibodies. The control was 408-TKO-2 AG-haESCs. Lamin B1 is a major structural component of the nucleus.^73^ **j** Representative images of gonads in E12.5 semi-cloned embryos (*Trim37*^Δ*NLS/+*^ and *Trim37*^Δ*NLS/ko1*^). Green fluorescence indicates expression of Oct4-EGFP and shows the location of PGCs. Three embryos were used for each group. Scale bars, 100 μm. **k** Immunoblot analysis showing expression levels of TRIM37 in the cytoplasm and nucleus of *Trim37*^*3×Flag*^ and *Trim37*^*NES*^ AG-haESCs. **l** Representative images of gonads in E12.5 semi-cloned embryos (*Trim37*^*NES/+*^ and *Trim37*^*NES/ko1*^). Green fluorescence indicates expression of Oct4-EGFP and shows the locations of PGCs. A *Stella* probe was used for ISH to identify PGCs. Three embryos were used for each group. Scale bars, 100 μm. **m** Immunoblot analysis of co-immunoprecipitated lysates from HEK293T cells co-transfected with Flag-tagged TRIM28 and HA-tagged full-length TRIM37 or the indicated mutants. Cell lysates were subjected to IP with anti-Flag affinity gel, followed by immunoblotting with anti-Flag, anti-HA, and anti-GAPDH antibodies.

To directly determine the requirement for TRIM28 in PGC development, we constructed *Trim28*^*flox/flox*^ mice and crossed them with *Blimp1-T2A-Cre* mice (Supplementary information, Fig. S9f−h). Phenotypic analysis showed that PGC numbers in *Trim28*^*cko*^ embryos were normal at E8.5 but decreased from E9.5 to E12.5 (Fig. 4a−e; Supplementary information, Fig. S9i). These results indicate that loss of TRIM28 results in defective PGC development.

### TRIM28-mediated nuclear retention of TRIM37 is essential for PGC development

To further investigate the interaction between TRIM37 and TRIM28, we overexpressed TRIM37 in HeLa cells and observed that the majority of TRIM37 localized to the cytoplasm (Fig. 4f). However, co-expression of TRIM28 with TRIM37 prompted the colocalization of TRIM37 and TRIM28 within the nucleus (Fig. 4f). This indicates that TRIM28 promotes nuclear accumulation of TRIM37. The G322V mutation in the TRIM37 MATH domain, which disrupts the interaction with TRIM28, dramatically decreased the nuclear accumulation of TRIM37 (Fig. 4f, g). Furthermore, compound R487E + V488E mutations, which interfere with the nuclear import of TRIM28,^41^ also disrupted nuclear accumulation of TRIM37 (Fig. 4f, g). These results indicate that TRIM28 facilitates the nuclear localization of TRIM37, suggesting that TRIM37 may play an essential role in the nucleus.

To determine whether nuclear localization is critical for TRIM37 function, we deleted a predicted nuclear localization sequence (NLS)^42^ in *Trim37*^*3×Flag*^ AG-haESCs (Fig. 4h). Western blot analysis showed that TRIM37 was reduced in the nuclei of *Trim37*^*ΔNLS*^ AG-haESCs (Fig. 4i). However, gonads derived from embryos generated by injection of *Trim37*^*ΔNLS*^ AG-haESCs into *Trim37*^*ko1*^ oocytes showed normal PGC development, possibly due to residual nuclear localization of TRIM37 (Fig. 4i, j). Therefore, to force TRIM37 to exit the nucleus, the NLS was replaced with a nuclear export signal (NES) sequence (*Trim37*^*NES*^) (Fig. 4h). Generation of AG-haESCs carrying *Trim37*^*NES*^ showed that nuclear TRIM37 was almost completely lost in *Trim37*^*NES*^ cells (Fig. 4k). In this case, and in contrast to the findings with *Trim37*^*ΔNLS*^, PGCs were nearly eliminated from *Trim37*^*NES/ko1*^ gonads at E12.5 (Fig. 4l). These changes occurred without affecting the TRIM37–TRIM28 interaction (Fig. 4m), suggesting that TRIM28 facilitates the nuclear function of TRIM37 during mouse PGC development.

### Ubiquitination of TRIM28 by TRIM37 protects PGC development

**Fig. 5:**
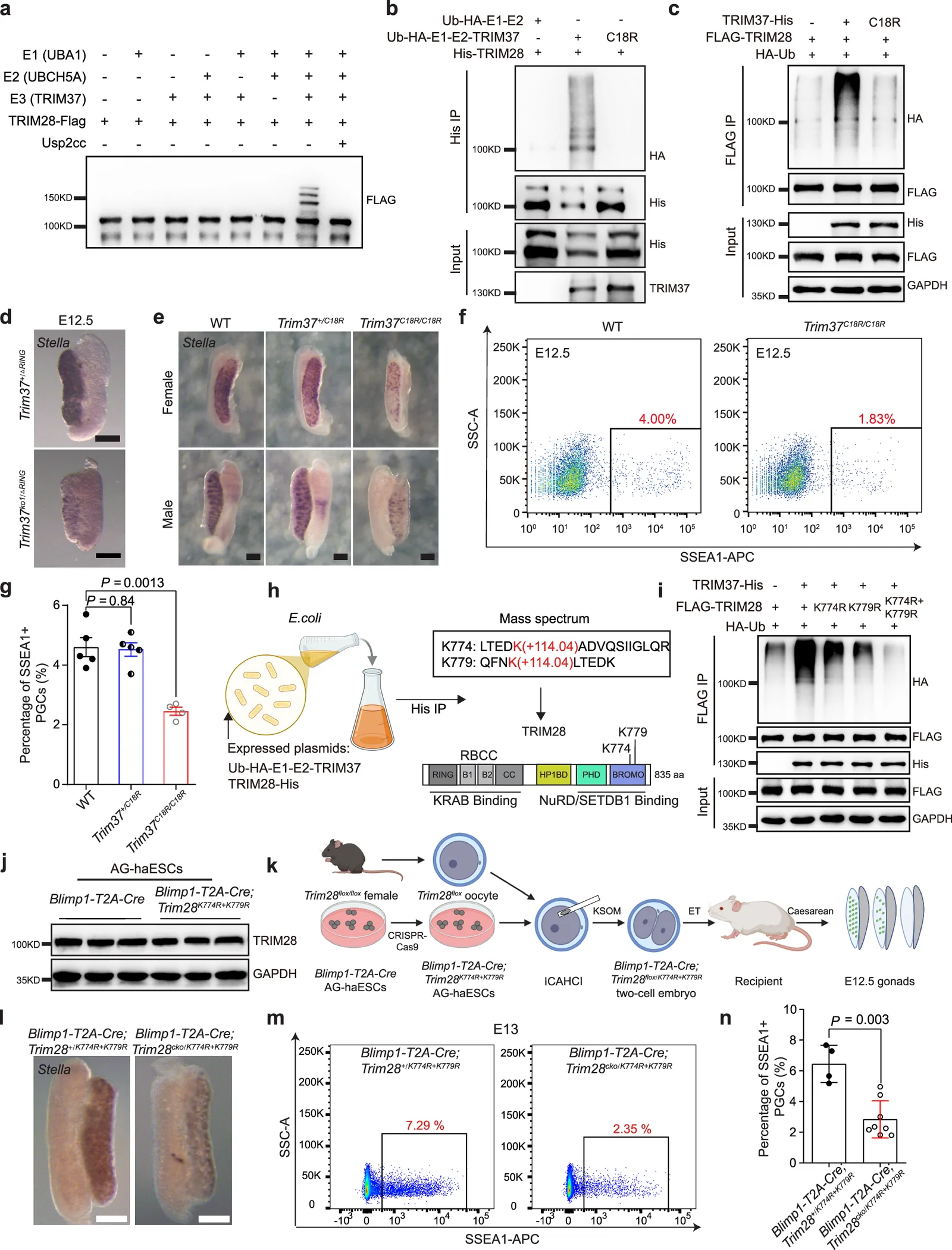
TRIM37 ubiquitinates TRIM28 at K774 and K779 to maintain PGC fate. **a** TRIM37 ubiquitinates TRIM28 in vitro. An in vitro ubiquitination assay was carried out using recombinant TRIM37, UBA1 (E1), UBCH5A (E2), and TRIM28 proteins together with the indicated components. Usp2cc, the catalytic core of human deubiquitinase Usp2. **b** Immunoblot analysis of the *E. coli* ubiquitination system in BL21 cells co-transformed with the pACYC vector expressing HA-tagged Ub, E1, E2, and either TRIM37 or TRIM37^C18R^ together with the pET22b-His-TRIM28 vector, using anti-HA, anti-His, and anti-TRIM37 antibodies. **c** Immunoblot analysis of lysates from HEK293T cells transfected with Flag-tagged TRIM28, HA-tagged Ub, and His-tagged TRIM37 or TRIM37^C18R^. **d** Representative images of E12.5 gonads from semi-cloned embryos (*Trim37*^*+/ΔRING*^ and *Trim37*^*ko1*/Δ*RING*^) analyzed by ISH using a *Stella* probe, showing significantly decreased PGC numbers in *Trim37*^*ko1/*Δ*RING*^ gonads. Three embryos were used for each group. Scale bars, 100 μm. **e** Representative images of E12.5 wild-type, *Trim37*^*+/C18R*^, and *Trim37* ^*C18R/C18R*^ gonads analyzed by ISH using a *Stella* probe, showing significantly decreased PGC numbers in *Trim37* ^*C18R/C18R*^ gonads. Five embryos were used for each group. Scale bars, 100 μm. **f** Representative FACS results showing SSEA1-positive PGCs from E12.5 wild-type and *Trim37* ^*C18R/C18R*^ gonads. **g** Box plots showing the percentage of SSEA1-APC-positive PGCs in E12.5 gonads (*n* = 5 embryos for wild-type, *n* = 5 embryos for *Trim37*^*+/C18R*^, and *n* = 4 embryos for *Trim37* ^*C18R/C18R*^). Data are presented as mean ± SEM, with each circle representing an individual embryo. *P*-values were determined using an unpaired two-sided Student’s *t*-test. **h** Schematic illustration of the strategy used to identify TRIM28 ubiquitination sites from *E. coli* lysates. Ubiquitination site mapping of TRIM28 was analyzed by MS. Both K774 and K779 are located in the BROMO domain of TRIM28. **i** Immunoblot analysis of lysates from HEK293T cells transfected with Flag-tagged TRIM28, TRIM28^K774R^, TRIM28^K779R^, or TRIM28^K774R+K779R^ together with the indicated plasmids. Lysates were immunoprecipitated with anti-Flag affinity gel and analyzed using anti-HA, anti-Flag, anti-His, and anti-GAPDH antibodies. **j** Western blots showing TRIM28 expression levels in *Blimp1-T2A-Cre* and *Blimp1-T2A-Cre;Trim28*^*K774R+K779R*^ AG-haESC lines, indicating that the K774R and K779R mutations do not affect TRIM28 stability. GAPDH was used as a loading control. **k** Schematic illustration of the analysis of the influence of conditional *TRIM28*^*K774R+K779R*^ mutations on PGC development through AG-haESC-mediated semi-cloning technology. **l** Representative images of *Blimp1-T2A-Cre*; *Trim28*^*+/K774R+K779R*^ and *Blimp1-T2A-Cre*; *Trim28*^*cko/K774R+K779R*^ gonads analyzed by ISH using a *Stella* probe, showing significantly decreased PGC numbers in *Blimp1-T2A-Cre*; *Trim28*^*cko/K774R+K779R*^ gonads. Three embryos were used for each group. Scale bars, 100 μm. **m**, **n** Representative FACS results (**m**) and statistical analysis (**n**) showing SSEA1-positive PGCs from *Blimp1-T2A-Cre*; *Trim28*^*+/K774R+K779R*^ (*n* = 4 embryos) and *Blimp1-T2A-Cre*; *Trim28*^*cko/K774R+K779R*^ gonads (*n* = 8 embryos). Data are presented as mean ± SEM, with each circle representing an individual embryo. *P*-values were determined using an unpaired two-sided Student’s *t*-test.

Previous studies indicate that TRIM37 is an E3 ubiquitin ligase.^43,44^ To assess whether TRIM37 directly ubiquitinates TRIM28, we performed in vitro ubiquitination reactions. The results showed that TRIM37 conjugated mono- and poly-Ub chains to TRIM28 as an E3 ligase (Fig. 5a). Simultaneous overexpression of TRIM28 and TRIM37 in *E. coli* resulted in robust ubiquitination of TRIM28 (Fig. 5b). Ubiquitination activity was dependent on a critical cysteine residue (C18) within the TRIM37 RING domain,^43^ as TRIM37^C18R^ failed to catalyze TRIM28 ubiquitination in *E. coli* (Fig. 5b). Consistently, in mammalian cells, ubiquitination of TRIM28 also required an intact C18 residue in TRIM37 (Fig. 5c). *Trim37* deletion in mice reduced ubiquitination of TRIM28 in tissues with high *Trim37* expression (brain and testis)^18^ (Supplementary information, Fig. S10a, b). Notably, the G322V mutation of TRIM37, which disrupts the TRIM37–TRIM28 interaction, abolished TRIM28 ubiquitination by TRIM37 (Supplementary information, Fig. S10c). Collectively, these findings suggest that TRIM37 can ubiquitinate TRIM28 and that this ubiquitination requires the MATH domain-mediated TRIM37–TRIM28 interaction.

To explore the role of the E3 ubiquitin ligase activity of TRIM37 in PGC development, we deleted the RING domain in AG-haESCs (*Trim37*^Δ*RING*^) (Supplementary information, Fig. S10d). Injection of these AG-haESCs into *Trim37*^*ko1*^ oocytes disrupted PGC development at E12.5 (Fig. 5d; Supplementary information, Fig. S10e, f). Consistent with this, *Trim37*^*C18R/C18R*^ mice exhibited reduced PGC numbers at E12.5 (Fig. 5e−g) and significantly impaired germ cell development in both testes and ovaries (Supplementary information, Fig. S10g, h). Together, these data indicate that the E3 ubiquitin ligase activity of TRIM37 is important for PGC development.

Canonical ubiquitination sites in substrates are characterized by Gly-Gly remnants on ubiquitinated lysine (K) residues.^45^ To identify the lysine residues in TRIM28 modified by ubiquitin, ubiquitinated TRIM28 proteins were immunopurified from *E. coli* lysates for MS analysis (Fig. 5h). K774 and K779 in the bromodomain of TRIM28 were identified as residues ubiquitinated by TRIM37 (Fig. 5h). Mutation of these lysine residues to arginine (R) reduced TRIM28 ubiquitination in HEK293T cells, especially when the mutations were combined (K774R + K779R) (Fig. 5i). The K774R + K779R mutations in TRIM28 also impaired the interaction between TRIM28 and TRIM37 (Supplementary information, Fig. S10i). Moreover, the C18R mutation in TRIM37 hindered its interaction with TRIM28 and its nuclear accumulation (Supplementary information, Fig. S10j, k), suggesting that ubiquitination of TRIM28 enhances its interaction with TRIM37.

To explore the effect of the TRIM28 double-KR mutation on PGC development, we first generated a K774R + K779R AG-haESC line based on *Blimp1-T2A-Cre* cells (Fig. 5j; Supplementary information, Fig. S10l). We then injected *Trim28*^*K774R+K779R*^ AG-haESCs into *Trim28*^*flox*^ oocytes to obtain conditional point-mutant embryos (Fig. 5k). ISH for *Stella* and flow cytometry revealed that TRIM28^K774R+K779R^ reduced PGC numbers at E12.5, partially mimicking the *Trim37* knockout phenotype (Fig. 5l−n). These results indicate that TRIM37-mediated ubiquitination of TRIM28 at K774 and K779 facilitates the nuclear accumulation of TRIM37, thereby playing an important role in PGC development.

### TRIM37 and TRIM28 act with AP2γ to remodel chromatin and repress somatic gene programs

**Fig. 6:**
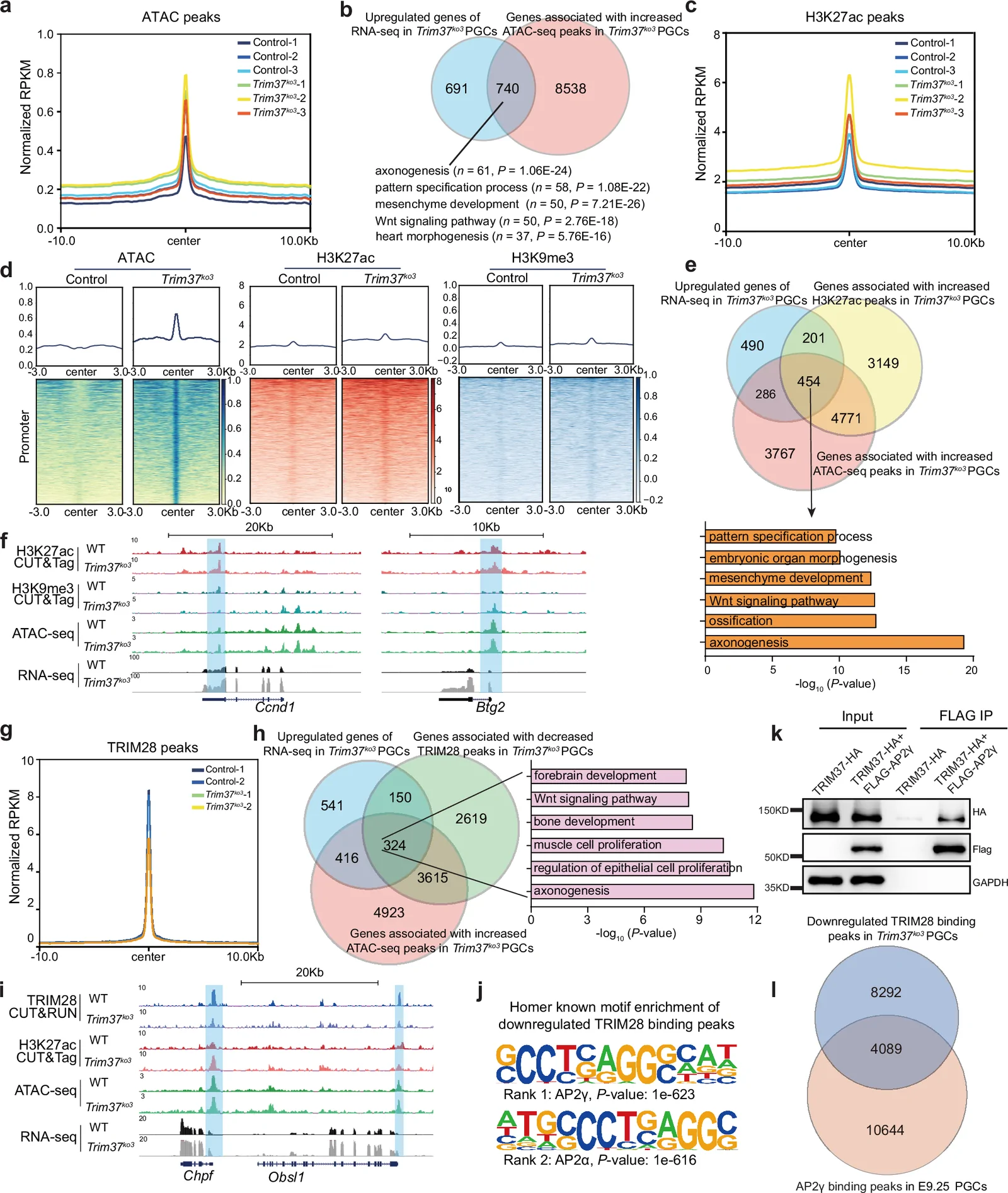
*Trim37* deficiency increases chromatin accessibility associated with developmental genes in PGCs. **a** Line plot displaying the intensity of centered average ATAC-seq signals in control and *Trim37*^*ko3*^ PGCs. **b** Venn diagram showing the overlap between upregulated genes from RNA-seq and upregulated peak-associated genes from ATAC-seq in *Trim37*^*ko3*^ PGCs. GO terms for the overlapping genes are presented beneath the diagram. “*n*” indicates the number of enriched genes. **c** Line plot displaying the intensity of centered average H3K27ac signals in control and *Trim37*^*ko3*^ PGCs. Regions extending 10-kb upstream and downstream from the peak centers are shown. **d** Line plots and heatmaps displaying ATAC-seq, H3K27ac, and H3K9me3 signals in control and *Trim37*^*ko3*^ PGCs at promoter regions with upregulated peaks identified by ATAC-seq. **e** Venn diagram showing the overlap between upregulated genes from RNA-seq, upregulated peak-associated genes from ATAC-seq, and upregulated peak-associated genes from H3K27ac Cleavage Under Targets and Tagmentation (CUT&Tag) in *Trim37*^*ko3*^ PGCs. GO terms for the overlapping genes are presented in the lower panel. **f** UCSC browser views displaying RNA-seq, ATAC-seq, H3K27ac, and H3K9me3 CUT&Tag signals for representative activated genes in *Trim37*^*ko3*^ PGCs, with blue-shaded boxes highlighting open chromatin regions. **g** Line plot displaying the intensity of centered average TRIM28 signals in control and *Trim37*^*ko3*^ PGCs. Regions extending 10-kb upstream and downstream from the peak centers are shown. **h** Venn diagram showing the overlap between upregulated genes from RNA-seq, upregulated peak-associated genes from ATAC-seq, and downregulated peak-associated genes from TRIM28 Cleavage under Targets & Release Using Nuclease (CUT&RUN) in *Trim37*^*ko3*^ PGCs. GO terms for the overlapping genes are presented in the right panel. **i** UCSC browser views displaying RNA-seq, TRIM28 CUT&RUN, H3K27ac CUT&Tag, and ATAC-seq signals for the *Chpf* and *Obsl1* regions in *Trim37*^*ko3*^ PGCs, with red-shaded boxes highlighting open chromatin regions. **j** HOMER motif prediction of downregulated TRIM28 binding peaks in *Trim37*^*ko3*^ PGCs. **k** HEK293T cells expressing the indicated proteins were immunoprecipitated with anti-Flag affinity gel and immunoblotted with anti-HA, anti-Flag, and anti-GAPDH antibodies. **l** Venn diagram showing the overlap between AP2γ binding peaks and downregulated TRIM28 binding peaks in E9.25 *Trim37*^*ko3*^ PGCs.

To further explore the molecular mechanisms underlying the repression of gene expression by the TRIM37–TRIM28 complex, we first explored the relationship between chromatin accessibility and gene transcription following *Trim37* inactivation using RNA-seq and ATAC-seq analyzes of PGCs at E9.25 (Supplementary information, Fig. S11a). Notably, upregulated genes in E9.25 *Trim37*^*ko3*^ PGCs were enriched in pattern specification and mesenchyme development (Supplementary information, Fig. S11b−e), consistent with the trends observed in scRNA-seq analyzes of E9.5 *Trim37*^*ko1*^ PGCs (Supplementary information, Fig. S7i, j). At the same time, *Trim37* deficiency increased global chromatin accessibility, with 27,919 upregulated peaks compared with 5215 downregulated peaks, particularly in regions associated with developmental genes (Fig. 6a; Supplementary information, Fig. S11f−h). More than 50% of upregulated genes linked to somatic development acquired open chromatin in *Trim37*^*ko3*^ PGCs (Fig. 6b).

TRIM28 is a crucial co-repressor involved in chromatin remodeling and transcriptional repression through the recruitment of repressor complexes, including SETDB1 and the NuRD complex.^19,36,38^ To explore which co-repressor may be recruited by the TRIM28 complex, H3K27ac and H3K9me3 profiles were analyzed in both control and *Trim37*^*ko3*^ PGCs using CUT&Tag (Supplementary information, Fig. S11a). Globally, *Trim37* deficiency led to increased H3K27ac levels at genomic regions associated with developmental processes (Fig. 6c; Supplementary information, Fig. S12a−c). In contrast, a subset of pattern specification-related genes exhibited altered H3K9me3 signals in *Trim37*-deficient PGCs, with both gains and losses detected (Supplementary information, Fig. S12d–f). However, the overall enrichment of H3K9me3 at these differential peaks remained very low (Supplementary information, Fig. S13a), suggesting that *Trim37* loss does not induce substantial changes in H3K9me3-mediated heterochromatin. Consistently, H3K9me3 displayed relatively low enrichment at promoters across the three gene groups analyzed (Supplementary information, Fig. S13b). By comparison, H3K27ac signals exhibited a modest but consistent increase at transcription start sites (TSSs) following *Trim37* knockout (Supplementary information, Fig. S13c), particularly in genes that became transcriptionally activated (FPKM ≤ 1 in control and FPKM ≥ 5 in *Trim37*^*ko3*^ PGCs) (Supplementary information, Fig. S13d). In addition, distal H3K27ac signals were clearly enriched at regulatory regions associated with genes upregulated in *Trim37*^*ko3*^ PGCs (Supplementary information, Fig. S13e), suggesting that enhancer activation also contributes to the transcriptional derepression observed in *Trim37*-deficient PGCs.

H3K27ac is regulated by the NuRD histone deacetylase complex and is closely associated with chromatin accessibility.^46,47^ Notably, regions showing enhanced chromatin accessibility in ATAC-seq data were also marked by elevated H3K27ac levels but low H3K9me3 across promoters, intergenic regions, and gene bodies in *Trim37*^*ko3*^ PGCs (Fig. 6d; Supplementary information, Fig. S14a). Approximately 454 of 1431 upregulated genes in *Trim37*^*ko3*^ PGCs exhibited open chromatin regions and increased H3K27ac signals in their regulatory regions (Fig. 6e). These genes, such as *Ccnd1* and *Btg2*, were enriched in axonogenesis, mesenchyme development, and embryonic organ morphogenesis (Fig. 6e, f). To further investigate the relationship between open chromatin regions and TRIM28 complex binding sites in *Trim37*-deficient PGCs, we performed CUT&RUN for TRIM28 in control and *Trim37*^*ko3*^ PGCs (Fig. 6g; Supplementary information, Fig. S14b). Among the differential TRIM28-binding peaks, 95.8% (12,381/12,922) were downregulated in *Trim37*^*ko3*^ PGCs and were associated with developmental genes (Supplementary information, Fig. S14b−d). Furthermore, ~324 of the 1431 upregulated genes in *Trim37*^*ko3*^ PGCs, including *Chpf*, *Obsl1*, and *Rnf122*, exhibited open chromatin regions and loss of TRIM28 binding in their regulatory regions (Fig. 6h, i; Supplementary information, Fig. S14e). These findings collectively indicate that TRIM37 deficiency compromises the ability of the TRIM28–NuRD complex to repress developmental genes through chromatin remodeling.

**Fig. 7:**
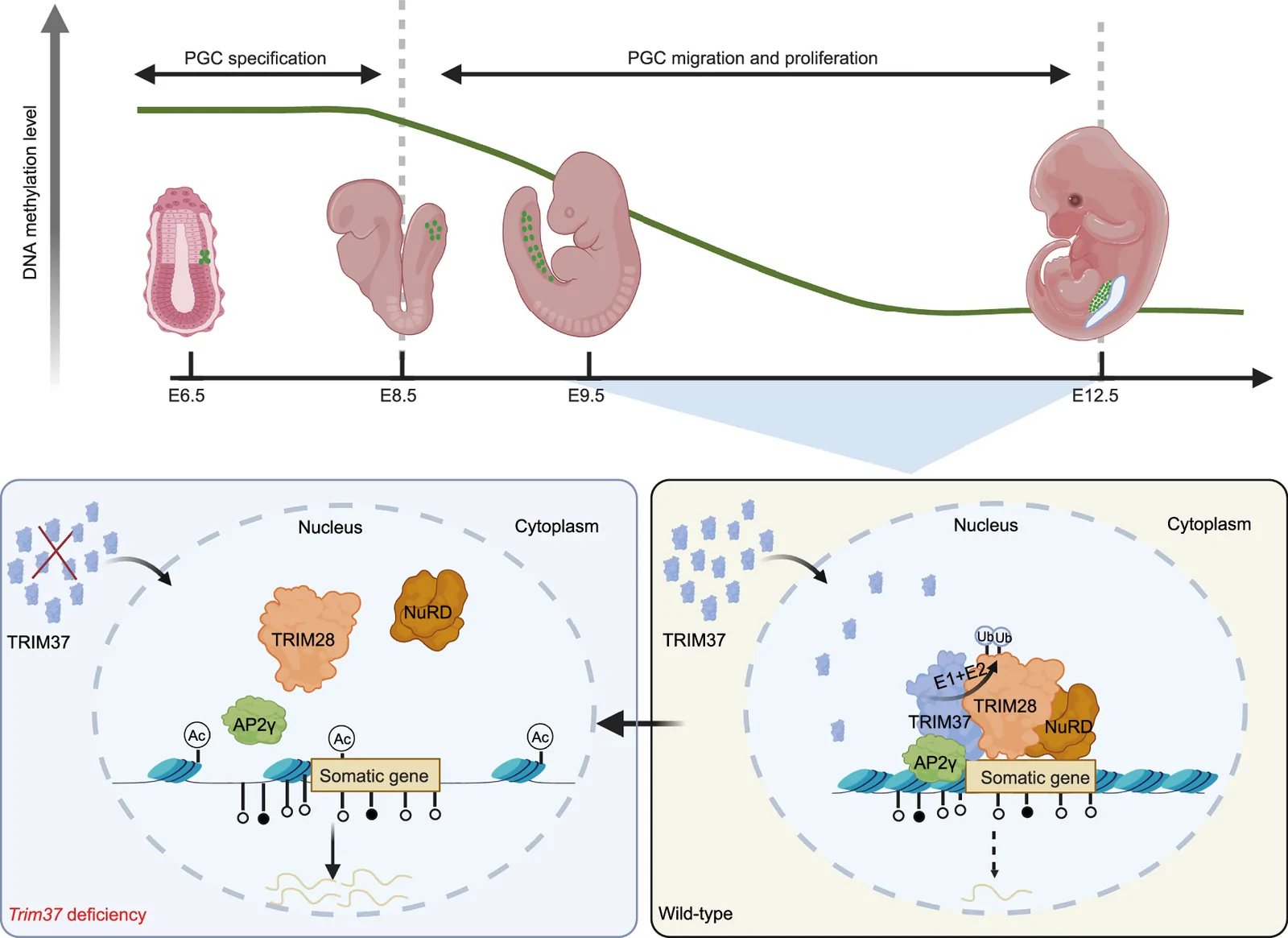
The TRIM37–TRIM28–AP2γ complex represses somatic programs to maintain migrating PGC identity. Schematic of mouse PGC development during migration, highlighting genome-wide DNA demethylation as a hallmark of epigenetic reprogramming.^2^ The TRIM37–TRIM28–AP2γ complex decreases chromatin accessibility and H3K27ac levels, thereby repressing somatic gene expression. This newly identified mechanism ensures the preservation of migrating PGC identity during extensive epigenetic resetting. PGCs are shown as green circles in the embryo.

Neither TRIM37 nor TRIM28 is a DNA-binding protein, and they are thus likely to act through intermediary sequence-specific DNA-binding proteins.^48,49^ We therefore performed an unbiased motif analysis of the TRIM28 downregulated peaks in *Trim37*^*ko3*^ PGCs. This analysis showed strong enrichment of AP2γ binding sites within the downregulated TRIM28 binding peaks (Fig. 6j). Previous studies have reported that AP2γ-null PGCs exhibit derepression of somatic genes in specified PGCs .^10^ AP2γ expression persists until E12.5 during PGC development,^10^ suggesting that AP2γ may function after germ cell specification. In line with this notion, conditional deletion of *Ap2γ* in PGCs after specification using *Blimp1-Cre* resulted in severe germ cell depletion, with only a few residual PGCs detectable at E9.5 and complete loss of PGCs by E12.5 (Supplementary information, Fig. S15a−f). We next performed a co-IP assay to assess a possible interaction between TRIM37 and AP2γ, which revealed interaction between these proteins in HEK293T cells (Fig. 6k). To examine the relationship between AP2γ occupancy and TRIM37–TRIM28-associated chromatin sites, we performed CUT&RUN for AP2γ using E9.25 PGCs (Supplementary information, Fig. S11a). Notably, 4089 of the 12,381 downregulated TRIM28 binding peaks in *Trim37*^*ko3*^ PGCs overlapped with AP2γ binding sites (Fig. 6l). Additionally, the downregulated TRIM28 binding peaks showed stronger AP2γ binding, which was also associated with developmental genes (Supplementary information, Fig. S16a, b). Together, the enrichment of AP2γ motifs at TRIM28-occupied loci, the physical interaction between TRIM37 and AP2γ, and their genomic co-occupancy support a model in which AP2γ may cooperate with, or help stabilize, the TRIM37–TRIM28 repressor complex at a subset of somatic gene loci. Consistent with this model, we propose that TRIM37 and TRIM28 act in concert with AP2γ to restrict inappropriate activation of somatic gene programs in migrating PGCs (Fig. 7).

## Discussion

The somatic program must be repressed to specify germ cells from the pluripotent epiblast.^9,50,51^ However, it remains uncertain whether the conflict between the germline and soma concludes after specification. Previous work showed that pluripotency transcription factors play important roles in PGC development, with conditional inactivation of either *Oct4* or *Sox2* resulting in loss of PGCs due to apoptosis or reduced proliferation, respectively.^52,53^ In addition, deletion of *Nanog* or *Esrrb* from the germline reduces PGC numbers by 50%−80% between E12.5 and E15.^54–56^ These studies have supported the prevailing hypothesis that, during migration, the PGC population is maintained through a balance between proliferation and apoptosis, with apoptosis representing the exclusive fate of mammalian PGCs that exit the germline due to genetic mutations or lack of somatic environmental cues.^52,57^ Moreover, developmentally compromised PGCs that evade apoptosis may serve as the origin of non-gonadal pediatric germ cell tumors.^58^ Our findings demonstrate that migrating germ cells rely on the TRIM37–TRIM28 repressor complex to actively repress somatic fate following specification.

A long-standing question in developmental biology is how germ cell fate, characterized by pluripotency and developmental immortality, is stably maintained during progressive somatic differentiation.^59^ In *C. elegans*, PGCs exhibit a pronounced propensity to adopt somatic cell identities upon loss of key epigenetic regulators, such as the histone chaperone LIN-53 or components of the polycomb repressive complex 2 (PRC2).^60,61^ Similarly, in zebrafish, depletion of *Dnd1* causes PGCs to undergo fate transformation, accompanied by marked alterations in cell morphology and shifts in transcriptional programs toward those of somatic lineages.^62^ However, whether these reprogrammed PGCs are capable of fully differentiating into functional somatic cell types remains unresolved.^62^ In this context, our results support a model in which *Trim37*-deficient PGCs exit the germline program and enter an aberrant somatic-like state marked by partial and heterogeneous activation of multiple somatic gene programs, rather than bona fide lineage-restricted transdifferentiation. As a future direction, we propose testing whether, in the absence of *Trim37*, PGCs progressively lose intrinsic germline determinants and fail to stabilize a coherent somatic identity because they do not receive appropriate lineage-specifying cues, ultimately resulting in elimination through cell death.

Mechanistically, mono- and poly-ubiquitination of TRIM28 by the RING domain of TRIM37 facilitates the interaction between TRIM28 and TRIM37 and promotes the nuclear accumulation of TRIM37, thereby enabling the TRIM37–TRIM28 complex to exert its repressive function. TRIM37 deficiency disrupted the binding profile of the TRIM28 repressor complex, leading to increased chromatin accessibility and H3K27ac modification at soma-specific loci and the upregulation of associated genes. Intriguingly, our data suggest that AP2γ, a critical transcription factor in PGC specification, might be responsible for the recruitment of the TRIM37–TRIM28 repressor complex to downstream target loci in migrating PGCs. Previous studies have shown that AP2γ enhances somatic gene repression and germline program activation during PGC specification.^10,12^ Considering that AP2γ is expressed in PGCs from E7.5 to E12.5, a period encompassing the entire germ cell migration period, our work supports the notion that AP2γ is not only important for PGC specification but also critical for the maintenance of germ cell fate in migrating PGCs. However, TRIM28 peaks only partially and inconsistently overlapped with regions of increased ATAC-seq signal or AP2γ binding sites, suggesting that additional mechanisms beyond the currently proposed TRIM37–TRIM28–AP2γ model may contribute to the regulation of somatic gene repression in PGCs. It is also possible that technical limitations, such as low cell number, epitope accessibility, or transient protein–DNA interactions, hinder accurate detection of TRIM28 or AP2γ chromatin binding in PGCs. Taken together, these observations support a model in which AP2γ likely cooperates with the TRIM37–TRIM28 complex at a subset of loci, while additional yet-to-be-identified factors may also contribute to targeting or stabilizing the complex at other sites.

Interestingly, TRIM37 mutants identified in MUL patients also lead to infertility in mouse models (*FIN*_*major*_ and G322V). Moreover, *AP2γ* and *TRIM28* are expressed together with *TRIM37* during human PGC development (Supplementary information, Fig. S16c), implying a conserved mechanism of TRIM37-mediated PGC regulation in humans and mice. Future studies using human PGCLC systems will be important to directly test this conservation, particularly through targeted disruption of TRIM37 or modeling of patient-associated mutations. In summary, our work outlines a comprehensive mechanism that protects germ cell fate from somatic differentiation in specified PGCs, providing a deeper understanding of germ cell biology and potentially offering implications for reproductive health and developmental biology.

## Materials and methods

### Mouse studies

All animal procedures were approved by the Institutional Animal Care and Use Committee of the Center for Excellence in Molecular Cell Science, Chinese Academy of Sciences. Mice were housed in individually ventilated cages (IVCs) in an accredited specific pathogen-free facility. The room was maintained at a controlled temperature (20−25 °C) and humidity (30%−70%) under a 12 h light-dark cycle. B6D2F1 (C57BL/6J × DBA2) female mice were used as oocyte donors. ICR female mice were used as pseudopregnant foster mothers. *Oct4-EGFP* and *Nanog-EGFP* transgenic mice have been described previously and were used for labeling PGCs.^23,32^ *Ap2γ*^*flox/flox*^ mice (RRID: IMSR-JAX:023408) were purchased from The Jackson Laboratory.^63^ *mTmG* mice (full name: B6.129(Cg)-*Gt(ROSA)26Sor*^*tm4(ACTB-tdTomato,-EGFP)Luo*^/J; JAX strain 007676) were used to trace PGCs. *Trim37*^*ko1*^, *Trim37*^*3×Flag*^, *Trim37*^*flox*^, *Trim37*^*C18R*^, *Trim37*^*G322V*^, and *FIN*_*major*_ mice were described and used in our recent work,^64^ which was conducted in parallel with the current study. In this study, we further generated additional mouse models, including *Trim37*^*ko2*^, *Trim37*^*ko3*^, *Trim37*^Δ*RING*^, *Trim37*^Δ*MATH*^, *Trim37*^Δ*NLS*^, *Trim37*^*NES*^, *Blimp1-T2A-Cre*, *Trim28*^*flox*^, and *Trim28*^*K774R+K779R*^. Embryos were collected from recipient mice and analyzed at specific developmental stages, including E8.5, E9.25, E9.5, E11.5, and E12.5, to assess PGCs.

### Plasmid construction

To generate CRISPR-Cas9 plasmids for gene manipulation in AG-haESCs, specific sgRNAs were synthesized, annealed, and inserted into the BpiI (Thermo Fisher Scientific, Waltham, MA, USA)-linearized pX330-mCherry plasmid (Cat# 98750; Addgene, Watertown, MA, USA). To generate donor plasmids, the left and right homologous arm sequences were amplified from genomic DNA using Phanta Max Super-Fidelity DNA Polymerase (Cat# P505; Vazyme, Nanjing, China) and then ligated into the PMD19T vector together with the knock-in sequence. To generate expression plasmids, wild-type and mutant CDSs of Cas9, TRIM37, TRIM28, and AP2γ were ligated into PiggyBac-CAG, pcDNA-CMV, or pACYC-Ub-HA plasmids. All inserted sequences were validated by Sanger sequencing.

### AG-haESC culture and transfection

AG-haESCs carrying deletions in the *H19*-DMR and *IG*-DMR regions, as well as an *Oct4-EGFP* transgene (termed *H19*^Δ*DMR*^-*IG*^*ΔDMR*^-AGH-OG3 or 408), were used to generate EFS-C9 and CAG-C9 cells for genetic screening. Enhanced AG-haESCs carrying *H19*, *H19*-DMR, and *IG*-DMR deletions together with an *Oct4-EGFP* transgene (termed *H19*^*Δ13kb*^-*IG*^*ΔDMR*^-AGH-OG3-2 or 408-TKO-2^65^), were used for the generation of *Trim37*^*3×Flag*^, *Trim37*^*ΔNLS*^, and *Trim37*^*NES*^ cells. AG-haESCs (named *IG*^*ΔDMR*^*-H19*^*ΔDMR*^-AGH-2 or O48^20^) were used for the generation of *Blimp1-T2A-Cre*, *Trim28*^*flox*^, *Trim37*^*ΔMATH*^, *Trim37*^*ΔRING*^, and *Trim28*^*K774R+K779R*^ cells. These AG-haESCs were cultured in 2i medium consisting of DMEM (Millipore, Billerica, MA, USA) supplemented with 15% FBS (ExCell Bio, Shanghai, China), penicillin–streptomycin, non-essential amino acids, nucleosides, L-glutamine, β-mercaptoethanol, 1000 U/mL LIF, 1 μM PD0325901 (Selleck Chemicals, Houston, TX, USA), and 3 μM CHIR99021 (Selleck Chemicals). Cells were passaged into a new 6-well plate at a density of 1.2 × 10^6^ cells per well, ~30 min before transfection in 2 mL of 2i medium and transfected with 250 µL of Opti-MEM containing 8 µL of Lipofectamine 3000 and 4−6 µg of plasmid DNA. After 18−24 h, mCherry-positive haploid cells were enriched by fluorescence-activated cell sorting (FACS) using an Aria II cell sorter (BD Biosciences, Franklin Lakes, NJ, USA) and seeded into a new well of a 6-well plate at a density of 6000−10,000 cells per well. After 6−8 days, single-cell-derived clones were picked and genotyped using PCR and Sanger sequencing.

### PGCLC differentiation

Differentiation was performed as previously described with minor modifications.^7,66^ Briefly, *Trim37-3*×*Flag* and *Trim37*-del ESCs were cultured in N2B27 medium supplemented with 2i/LIF for at least four passages. Subsequently, cells were seeded into 12-well plates coated with 10 ng/mL fibronectin at a density of 1 × 10^5^ cells per well in N2B27 medium supplemented with 1% KSR, bFGF, and activin A to generate epiblast-like cells (EpiLCs). After 2 days, EpiLCs were collected and aggregated at a density of 2000 cells per 25 μL drop in PGCLC medium containing 50 ng/mL BMP4, 50 ng/mL BMP8a, 10 ng/mL SCF, 10 ng/mL EGF, and 1000 U/mL LIF. The drops were plated onto the lids of tissue culture dishes and incubated over a PBS reservoir for 2 days. Subsequently, the hanging drops were transferred to untreated culture dishes and cultured in PGCLC medium with rotation at 72 rpm. After 2 days (PGCLC day 4) and 4 days (PGCLC day 6), aggregates were collected and dissociated into single cells using 0.05% trypsin for 10 min. The dissociated cells were stained with anti-SSEA1 eFluor™ 660 (1:20 dilution; Cat# 50-8813-41; Invitrogen, Carlsbad, CA, USA) and anti-CD61 PE (1:50 dilution; Cat# 104307; BioLegend, San Diego, CA, USA) and then analyzed by flow cytometry.

### Construction of the lentiviral sgRNA library

sgRNA sequences targeting each gene were selected based on previous work^67^ and synthesized by Sangon Biotech, Shanghai, China. All sgRNA sequences were annealed, mixed thoroughly, and then ligated into the BbsI-linearized pKLV-U6(BbsI)-PGKpuro2ABFP vector (Cat# 50946; Addgene) with T4 DNA ligase (NEB, Ipswich, MA, USA). To assess library coverage, we randomly picked more than ten *E. coli* clones from each dish to ensure that different clones contained different sgRNAs. Bacteria from 5 × 10-cm dishes were scraped and combined for purification of sgRNA library plasmids using an EndoFree Maxi Plasmid kit (Tiangen, Beijing, China). The method used to produce lentivirus from the sgRNA library plasmids and the lentiCas9-Blast plasmid (Cat# 52962; Addgene) was similar to that described in our previous work.^23^ For the lentiviral sgRNA library, virus volumes were optimized to achieve a multiplicity of infection of 0.3, ensuring that most cells received a single copy of the lentiviral vector. EFS-C9 or CAG-C9 AG-haESCs were infected with lentivirus for ~48 h and then selected with puromycin for 48 h. The infected cells were passaged and sorted for haploid cells by flow cytometry for ICAHCI and subsequently collected for genomic DNA extraction.

### Generation of embryos and mice via ICAHCI

To generate semi-cloned embryos and mice via ICAHCI, haploid cells carrying different gene modifications were arrested in M phase by culture in 2i medium containing 0.05 μg/mL demecolcine for 12 h. Arrested haploid cells were trypsinized and suspended in HCZB medium for ICAHCI. MII oocytes were collected from the oviducts of superovulated B6D2F1 females (8 weeks old), *Trim37*^*ko1*^ heterozygotes (6−10 weeks old), or *Trim28*^*flox*^ homozygotes (6−10 weeks old). A single haploid cell was then injected into the cytoplasm of an MII oocyte using a Piezo-drill micromanipulator. The reconstructed zygotes were cultured in CZB medium for 30 min and then activated for 5−6 h in Ca^2+^-free CZB with SrCl_2_. After activation, the reconstructed embryos were cultured in AA-KSOM medium (Merck, Darmstadt, Germany) at 37 °C in 5% CO_2_. After 18−24 h, two-cell embryos were transferred into the oviducts of 0.5 days post coitum (dpc) pseudopregnant ICR females (18−23 embryos per oviduct). Embryos were dissected from recipient mice at specific stages after transplantation, and gene-modified pups were obtained from recipient mice at E19.5.

### Deep sequencing and analysis

To analyze the distribution of sgRNAs in Trim-library-1 and Trim-library-2 haploid cells, genomic DNA was extracted from the cells. sgRNA inserts were amplified from the extracted DNA using Phanta® Max Super-Fidelity DNA Polymerase (Vazyme). Libraries were prepared using adapter-modified PCR and sequenced on a HiSeq X Ten platform (2 × 150 bp PE; Illumina, San Diego, CA, USA) at Annoroad Gene Technology Corporation, Beijing, China. The frequencies of different sgRNAs were determined by mapping clean reads to the reference sequence.

### RNA extraction and real-time PCR

Total RNA was isolated from AG-haESCs using TRIzol reagent (Invitrogen). Approximately 1 µg of RNA was reverse transcribed into cDNA using a Wiper kit (Vazyme). Real-time PCR (RT-PCR) was performed on a Mastercycler Nexus Gradient GSX1 thermal cycler (Eppendorf, Hamburg, Germany) using Phanta® Max Super-Fidelity DNA Polymerase. Approximately 10 PGCs were isolated from E9.5 embryos using a Piezo-drill micromanipulator and transferred into a 200-µL tube containing 3.5 µL of lysis buffer. The lysate was then used for cDNA amplification with a Single Cell Full-Length mRNA Amplification Kit (Cat# N712; Vazyme). Real-time quantitative PCR (RT-qPCR) was performed in triplicate on a CFX96 system (Bio-Rad, Hercules, CA, USA) using SYBR Green Mix (Toyobo, Osaka, Japan). The expression level of *Trim37* was normalized to that of the internal control gene *Gapdh*.

### Base editing-mediated gene knockout in the embryo

To trace PGC fate after *Trim37* knockout, we adopted a base editor previously reported in our recent work^33^ to efficiently inactivate genes in zygotes. The mRNA of an efficient base editor, hA3A-eBE3-Y130F, and *Trim37-*sgRNA-stop were transcribed using an mMESSAGE mMACHINE T7 ULTRA Transcription Kit (Cat# AM1345; Invitrogen) and a MEGAshortscript T7 Kit (Cat# AM1354; Invitrogen), respectively, according to the manufacturer’s instructions. mRNAs and sgRNAs were subsequently purified using a MEGAclear Transcription Clean-Up Kit (Cat# AM1908; Invitrogen), resuspended in hot (95 °C) RNase-free water, and stored at −80 °C. The mixture of hA3A-eBE3-Y130F mRNA (100 ng/μL) and *Trim37-*sgRNA-stop (100 ng/μL) was diluted in RNase-free water, centrifuged at 13,400 × *g* for 10 min at 4 °C, and injected into the cytoplasm of zygotes (*mTmG*♀ × *Blimp1-T2A-Cre*♂ or B6D2F1♀ × *Nanog-EGFP*♂) using a micromanipulator (Olympus, Tokyo, Japan) and a FemtoJet microinjector (Eppendorf). The resulting embryos were transferred into recipient mice and collected at specific stages for analysis of PGC development.

### Hematoxylin-eosin staining

Testes, epididymides, and ovaries were fixed in 4% paraformaldehyde overnight at room temperature, dehydrated through a graded ethanol series (50%, 60%, 70%, 80%, 95%, and 100%; diluted with distilled water), embedded in paraffin, and sectioned. Serial 4-μm cross sections were mounted on slides, deparaffinized in xylene, and rehydrated through graded ethanol concentrations (100%, 95%, 70%, and 50%; diluted with distilled water), followed by staining with hematoxylin and eosin. Images were captured using a BX51 microscope (Olympus).

### Isolation of PGCs by FACS

Dissected gonads (E11.5 and E12.5) and E9.25 embryos were enzymatically dissociated using 100 µL of 0.25% trypsin (Cat# 25200072; Thermo Fisher Scientific) at 37 °C for 8 min. Trypsin activity was quenched by adding 200 μL of MEF medium (10% FBS in DMEM), and the cell suspensions were centrifuged at 300× *g*. Cells from *Trim37*^*ko1*^ gonads were directly resuspended in MEF medium, passed through a 40-µm cell strainer into a 5-mL Falcon Corning FACS tube, and subjected to FACS. Oct4-EGFP- or Nanog-EGFP-positive PGCs were sorted using the FITC channel and used for further analysis. Cells from *Trim37*^*C18R*^ and *Trim28*^*K774R+K779R*^ gonads were resuspended in FACS buffer (0.1% BSA in PBS), stained with anti-SSEA1 eFluor™ 660 (1:20 dilution; Cat# 50-8813-41; Invitrogen), and then subjected to FACS. SSEA1-positive PGCs were sorted and collected for further analysis.

### Whole-mount embryo alkaline phosphatase staining and ISH

For alkaline phosphatase staining, E8.5 embryos were fixed in 4% paraformaldehyde for 15 min at room temperature or overnight at 4 °C and then washed twice with TBST. The embryos were then incubated in 70% ethanol for 8 h at 4 °C, stained in freshly prepared alkaline phosphatase staining solution for 5−7 min at room temperature, and submerged in 80% glycerin until they sank to the bottom.

For ISH, gonads and E8.5 embryos were first fixed in 4% paraformaldehyde overnight at 4 °C. Subsequently, samples were dehydrated through a graded methanol series (25%, 50%, and 75%; diluted with PBS containing 0.1% Tween-20 (PBST)) and then stored in 100% methanol at −20 °C. The dehydrated embryos were rehydrated and subjected to prehybridization in prehybridization solution at 68 °C for 5 h. Embryos were hybridized with a digoxigenin-labeled *Stella* riboprobe generated by in vitro transcription from a linearized template using a DIG RNA Labelling Kit (Cat# 11175025910; Roche, Basel, Switzerland), and hybridization was carried out overnight at 68 °C. Next, embryos were blocked overnight at 4 °C in blocking solution containing an anti-DIG antibody conjugated to alkaline phosphatase (Cat# 11093274910; Roche). Finally, embryos were stained with 20 μL/mL NBT/BCIP (Cat# 11681451001; Roche) at room temperature and submerged in 80% glycerin until they sank to the bottom.

### Whole-mount embryo immunostaining

E9.5 embryos (*Trim37*^*ko1*^ and *Trim37*^*cko*^) were harvested, fixed in 4% paraformaldehyde at 4 °C for 2 h, and washed twice with PBS. Next, embryos were blocked overnight at 4 °C in blocking buffer (5% donkey serum, 1% BSA, and 0.3% Triton X-100 in PBS) before primary antibody incubation. *Trim37*^*ko1*^ and *Trim37*^*cko*^ embryos were incubated for 48 h at 4 °C with anti-GFP (1:2000 dilution; Cat# ab13970; Abcam, Cambridge, UK) and anti-STELLA (1:200 dilution; Cat# ab19878; Abcam), respectively, diluted in blocking buffer. Samples were then incubated at room temperature for 24 h with goat anti-chicken Alexa Fluor 488 (1:1000 dilution; Cat# ab150169; Abcam), donkey anti-rabbit Alexa Fluor 488 (1:1000 dilution; Cat# A-21206; Thermo Fisher Scientific), and DAPI (1 μg/mL; Cat# D8417; Sigma, St. Louis, MO, USA) diluted in PBST. Subsequently, samples were washed overnight at room temperature with washing buffer (0.2% Triton X-100 and 0.5% 1-thioglycerol in PBS) and then cleared for 36 h at room temperature in clearing medium (40% N-methylacetamide, 1.455 g/mL Histodenz, 0.1% Triton X-100, and 0.5% 1-thioglycerol in PBS). Samples were immersed in fresh clearing medium and sealed with vacuum grease for imaging.

### Embryo and cell immunostaining

For section immunostaining, E9.5 embryos were collected, washed with cold PBS, fixed in 4% paraformaldehyde overnight at 4 °C, and dehydrated through a graded sucrose series (10%, 20%, and 30%; diluted with distilled water). Embryos were embedded in an appropriate volume of Neg 50 Frozen Section Medium (Thermo Fisher Scientific), rapidly frozen in liquid nitrogen, and stored at −80 °C. Approximately 10-μm-thick cross-sections were mounted on positively charged microscope slides.

For cell immunostaining, HeLa cells were transfected with different plasmids for 12 h and fixed in 4% paraformaldehyde for 1 h at room temperature. Embryo sections or fixed cells were permeabilized with 0.2% Triton X-100 for 15 min at room temperature and blocked with 2.5% BSA for 1 h at room temperature. Samples were incubated overnight at 4 °C with primary antibodies diluted in 2.5% BSA at the indicated dilutions. The primary antibodies used in this study were anti-GFP (1:2000 dilution; Cat# ab13970; Abcam), anti-STELLA (1:200 dilution; Cat# ab19878; Abcam), anti-Oct3/4 (1:200 dilution; Cat# sc-5279; Santa Cruz Biotechnology, Dallas, TX, USA), anti-AP2γ (1:200 dilution; Cat# sc-12762; Santa Cruz Biotechnology), anti-cleaved caspase-3 (1:400 dilution; Cat# 9661; Cell Signaling Technology, Danvers, MA, USA), anti-KAP1 (1:500 dilution; Cat# ab10483; Abcam), anti-MVH (1:200 dilution; Cat# ab270534; Abcam), anti-HA (1:1000 dilution; Cat# 3724S; Cell Signaling Technology), and anti-Flag (1:1000 dilution; Cat# F1804; Sigma). Samples were then incubated for 1 h at room temperature with secondary antibodies diluted in PBST, including goat anti-chicken Alexa Fluor 488 (1:1000 dilution; Cat# ab150169; Abcam), donkey anti-rabbit Alexa Fluor 488 (1:1000 dilution; Cat# A-21206; Invitrogen), goat anti-rabbit Cy3 (1:1000 dilution; Cat# 111-165-144; Jackson ImmunoResearch, West Grove, PA, USA), goat anti-rabbit Alexa Fluor 647 (1:1000 dilution; Cat# A21245; Invitrogen), goat anti-mouse Cy3 (1:1000 dilution; Cat# 115-165-146; Jackson ImmunoResearch), and goat anti-mouse Alexa Fluor 488 (1:1000 dilution; Cat# A11029; Invitrogen). Sections were washed three times with PBST, and DNA was stained with DAPI (1 μg/mL; Cat# D8417; Sigma) for 15 min. Fluorescent images were captured using a TCS SP8 laser scanning confocal microscope (Leica, Wetzlar, Germany) and a BX51 microscope.

### Nuclear and cytoplasmic extract isolation

Control and *Trim37*^*3×Flag*^ AG-haESCs were cultured in 10-cm dishes, washed twice with ice-cold PBS, trypsinized, and centrifuged at 1000 rpm for 5 min. Cell pellets were gently resuspended in 400 µL of lysis buffer (10 mM Tris, pH 8.0, 140 mM NaCl, 1.5 mM MgCl_2_, and 0.5% Igepal) containing 1× protease inhibitor cocktail (Cat# 11697498001; Sigma) and incubated on ice for 3 min. The lysate was centrifuged at 1000 × *g* for 3 min at 4 °C to separate the nuclei from the cytoplasmic fraction. To obtain pure nuclear protein, nuclear pellets were washed twice with 350 µL of lysis buffer and once with lysis buffer containing 0.5% deoxycholic acid. To obtain pure cytoplasmic protein, the supernatant fraction was centrifuged at 13,000 rpm for 10 min at 4 °C, and the resulting supernatant was carefully collected into a new tube. Finally, the purified nuclear and cytoplasmic fractions were boiled in 60 µL of 2× SDS loading buffer (Cat# P0015L; Beyotime Biotechnology, Shanghai, China) for 15 min at 100 °C. Nuclear and cytoplasmic extracts were analyzed by western blot.

### Co-IP

Briefly, HEK293T cells were cultured in 6-cm dishes and transfected with various combinations of expression plasmids using polyethyleneimine (Cat# 23966-1; Polysciences, Warrington, PA, USA) according to the manufacturer’s instructions. After 24 h, cells were washed twice with cold PBS and lysed in immunoprecipitation buffer (50 mM Tris-Cl, 150 mM NaCl, 5 mM EDTA, and 1% NP-40, pH 7.8) containing 1× protease inhibitor cocktail (Sigma) on ice for 1 h. The lysates were centrifuged at 13,200 rpm for 30 min, and the resulting supernatants were used for immunoprecipitation with Anti-Flag Affinity Gel (Cat# A2220; Sigma) or Anti-HA Affinity Gel (Cat# GNI4510-HA; GNI, Shanghai, China) overnight at 4 °C. Beads were washed five times with low- and high-salt lysis buffers, and bound proteins were eluted by boiling the beads in 1× SDS loading buffer for 15 min at 100 °C, followed by immunoblotting analysis.

### MS analysis of TRIM37 IP

Gonads (30−40 per sample) were collected from wild-type and *Trim37*^*3×-Flag*^ embryos and lysed in buffer containing 50 mM Tris-HCl, pH 7.5, 200 mM NaCl, and 1% NP-40. The lysates were subjected to IP using Anti-Flag Affinity Gel. Bound proteins were eluted by boiling the beads in 1× SDS loading buffer for 15 min at 100 °C, followed by electrophoresis. The gel was stained with Coomassie brilliant blue (CBB) and divided into three sections based on protein size: band A (≥ 60 kDa), band B (15−35 kDa and 45−65 kDa), and band C (< 15 kDa or 30−50 kDa). MS analysis of the gel sections was performed by Novogene Co., Ltd (Beijing, China). Subsequently, protein identification and quantification were performed using Proteome Discoverer software (v1.4; Thermo Fisher Scientific) based on the original spectra. A value of “0” indicates that although the protein was identified, its peak area was too small for quantification and was therefore reported as 0. Candidate proteins were selected based on size and presence in all three *Trim37*^*3×Flag*^ replicates but were nearly absent from control samples.

### *E. coli* ubiquitination system reconstitution and MS

All components of the ubiquitination system were cloned into the pACYCDuet-1 vector (Novagen, Madison, WI, USA) using standard cloning techniques. HA-tagged ubiquitin (HA-Ub), UBCH5A, and UBA1 coding sequences were inserted into the first multiple cloning site (MCS) under the control of the T7 promoter/lac operator and ribosome-binding sites. These elements were separated by Shine-Dalgarno sequences to ensure proper expression. The mouse TRIM37 coding sequence was inserted into the second MCS, generating the plasmid pACYC-HA-Ub-UBCH5A-TRIM37. pACYC-HA-Ub-UBCH5A-TRIM37 and pET22b-TRIM28 plasmids were co-transformed into *E. coli* BL21 cells and were selected with ampicillin and chloramphenicol. Protein expression in the BL21 strain was induced with 0.3 mM isopropyl β-D-1-thiogalactopyranoside (IPTG; Sigma) at an OD600 of 0.6, followed by incubation for 20 h at 16 °C. Cells were harvested and lysed in lysis buffer containing 8 M urea, 300 mM NaCl, 50 mM Na_2_HPO_4_, 50 mM Tris-HCl, and 0.5% NP-40, pH 8.0. Lysis was performed using an ultrasonic homogenizer (JY92-1IN; Ningbo Scientz Biotechnology Co., Ltd, Ningbo, China). Ubiquitinated proteins were enriched using Ni-NTA agarose beads (Sangon Biotech, Shanghai, China) and subsequently analyzed by immunoblotting or MS. TRIM28 protein was purified from the reconstituted *E. coli* system and dissolved in elution buffer containing 8 M urea and 50 mM Tris-HCl, pH 8.0. The dissolved protein was subjected to trypsin digestion, and the resulting peptides were separated using an EASY-nLC system (Thermo Fisher Scientific). MS analysis was performed on a Q Exactive mass spectrometer (Thermo Fisher Scientific). Protein and ubiquitination data were analyzed using Thermo Proteome Discoverer 2.1 software (Thermo Fisher Scientific), with searches conducted against the UniProt Human database (http://www.uniprot.org/).

### Ubiquitination assay in cells

HEK293T cells were transfected with the specified vector combinations. After 48 h, cells were harvested and lysed in buffer A (50 mM Tris-Cl, 150 mM NaCl, 1% NP-40, 1% sodium deoxycholate, 1% SDS, pH 7.6). The lysates were boiled for 10 min at 95 °C, diluted tenfold with buffer B (50 mM Tris-Cl, 150 mM NaCl, pH 7.6), and sonicated. Immunoprecipitation was performed overnight at 4 °C using an anti-TRIM28 antibody coupled to agarose beads (Cat# 16-266; Merck Millipore, Darmstadt, Germany) or Anti-Flag Affinity Gel (Cat# A4596; Sigma). The immunoprecipitates were washed three times with RIPA buffer (50 mM Tris-Cl, 150 mM NaCl, 1% NP-40, 0.5% sodium deoxycholate, 0.1% SDS, pH 7.6), boiled in 2× SDS-PAGE sample buffer, and analyzed by immunoblotting with the indicated antibodies. Tissue samples were homogenized in buffer A using a Touch Homogenizer (Precellys 24; Bertin, Montigny-le-Bretonneux, France), followed by processing as described above.

### In vitro ubiquitination assay

In vitro ubiquitination assays were performed as previously described.^68^ Briefly, the reaction mixture (final volume: 50 μL) contained 50 ng of His6-Ub, 20 ng of UBA1-His6 (E1), 50 ng of UBCH5A-His6 (E2), 100 ng of E3 (TRIM37 or its mutant)-SUMO-His6, and 100 ng of TRIM28-Flag in ubiquitination buffer (25 mM Tris-Cl, 100 mM NaCl, 1 mM DTT, 5 mM MgCl_2_, pH 7.6) supplemented with freshly added 2 mM ATP. The reaction was incubated for 1 h at 37 °C and then denatured by heating for 10 min at 100 °C in 1× SDS-PAGE loading buffer. The ubiquitination level of TRIM28 was analyzed by immunoblotting using an anti-Flag antibody.

### Protein expression and purification

The codons of TRIM37 and TRIM28 were optimized for expression in *E. coli*. Full-length mouse TRIM37 (residues 1–961) and its mutants were cloned into a modified pET28a vector featuring a SUMO protein fused at the N-terminus and a 6×His tag at the C-terminus. Full-length mouse TRIM28 (residues 1–1067) and its mutants were cloned into the pET-41a vector, with a GST tag fused at the N-terminus. The optimized gene sequences were synthesized and ligated into the vectors by Tsingke Biological Technology (Beijing, China), where the proteins were also purified. In brief, *E. coli* BL21 (DE3) competent cells carrying expression plasmids were induced with 0.2 mM isopropyl β-D-1-thiogalactopyranoside (IPTG) for 18 h at 16 °C, after which the cells were collected by centrifugation. The cell pellets were resuspended in TBS with 1 mM phenylmethylsulfonyl fluoride (PMSF) and disrupted by sonication on ice. The mixture was then centrifuged at 12,000 × *g* for 10 min. The supernatant was incubated with Ni-NTA resin, washed with PBS, pH 7.4, and sequentially eluted with PBS buffer, pH 7.4, containing 25 mM, 50 mM, 100 mM, and 200 mM imidazole. The collected protein was dialyzed against PBS at a 1:50 ratio overnight, followed by replacement of the dialysis solution and further dialysis for an additional 8 h. Proteins were concentrated using PEG 20,000. Purified proteins were used immediately or stored at −80 °C.

### GST pull-down assay

Purified GST-TRIM28 or its mutant (20 μg) was incubated with TRIM37-SUMO-His6 (20 μg) overnight at 4 °C with Glutathione Sepharose 4B beads (GE Healthcare, Chicago, IL, USA) in 1 mL pull-down buffer (20 mM Tris-Cl, 100 mM NaCl, 5 mM MgCl_2_, 1 mM EDTA, 1 mM DTT, 0.5% NP-40, 10 μg/mL BSA, pH 7.5). The beads were pelleted and washed five times with the same pull-down buffer. Bound proteins were denatured by heating the beads in 2× SDS-PAGE loading buffer for 10 min at 100 °C and analyzed by immunoblotting.

### Immunoblotting analysis

Cells and tissues were lysed in 1× RIPA buffer (Cat# 9806S; Cell Signaling Technology) containing 50 mM Tris-Cl, pH 7.4, 150 mM NaCl, 5 mM EDTA, 1% Triton X-100, 0.5% sodium pyrophosphate, and 0.1% sodium dodecyl sulfate and supplemented with protease inhibitor cocktail (Cat# P8340; Sigma) on ice for 1 h. Before gel electrophoresis, protein lysates were boiled in 5× loading buffer for 10 min at 100 °C. Protein lysates from different assays were separated by 10% SDS-PAGE, blotted onto a polyvinylidene difluoride (PVDF) membrane, blocked in blocking solution (10% non-fat dry milk in TBST buffer) for several hours, and incubated overnight at 4 °C in TBST buffer with the appropriate primary antibody (anti-Flag, 1:5000 dilution, Cat# M20008M (Abmart, Shanghai, China); anti-HA, 1:1000 dilution, Cat# 3724S (Cell Signaling Technology); anti-TRIM37, 1:1000 dilution, Cat# 96167 (Cell Signaling Technology); anti-KAP1, 1:2000 dilution, Cat# ab10483 (Abcam); anti-Lamin B1, 1:5000 dilution, Cat# ab16048 (Abcam); anti-HRP-conjugated His, 1:20,000 dilution, Cat# HRP-66005 (Proteintech, Rosemont, IL, USA); anti-HRP-conjugated GST, 1:20,000 dilution, Cat# HRP-66001 (Proteintech); anti-Ub, 1:5000 dilution, Cat# 10201-2-AP (Proteintech); and anti-GAPDH, 1:5000 dilution, Cat# 60004-1-Ig (Proteintech)). The membranes were washed three times for 10 min each with TBST and incubated for 1 h at room temperature in TBST with the appropriate secondary antibody (Goat anti-rabbit IgG, 1:5000 dilution, Cat# D110058-0001 (BBI Life Sciences, Shanghai, China) or goat anti-mouse IgG, 1:10,000 dilution, Cat# SA00001-1 (Proteintech)). Chemiluminescence detection was performed using an ECL reagent solution kit (ShareBio, Shanghai, China).

### scRNA-seq and data analysis

E8.5 *Nanog-EGFP* transgenic embryos and E9.5 *Oct4-EGFP* transgenic embryos were dissected from recipient mice and digested with 0.25% Trypsin-EDTA (Cat# 25200072; Thermo Fisher Scientific) at 37 °C. After digestion, embryos were gently pipetted and resuspended in HCZB medium. E8.5 *Trim37*^*ko3*^ embryos were generated by injecting hA3A-eBE3-Y130F mRNA and *Trim37*-sgRNA-stop into the cytoplasm of zygotes from *Nanog-EGFP* males mated with BDF1 females. E9.5 *Trim37*^*ko1*^ embryos were obtained by mating *Oct4-EGFP/Trim37*^*ko1*^ heterozygous males with *Trim37*^*ko1*^ heterozygous females. Single PGCs from wild-type and *Trim37* knockout embryos were isolated using a Piezo-drill micromanipulator and transferred into a 200-µL-tube containing 3.5 µL of lysis buffer. The lysate was used for cDNA amplification with a Single Cell Full-Length mRNA Amplification Kit (Cat# N712; Vazyme). RNA-seq libraries were constructed using a TruePrep DNA Library Prep Kit V2 (Cat# TD503; Vazyme) for Illumina. Sequencing was performed on a NovaSeq 6000 (Illumina) to generate 150-bp paired-end reads by Genergy Biotechnology Co., Ltd (Shanghai, China) according to the manufacturer’s instructions. Quality control of the raw FASTQ data was conducted using Trim Galore (v0.6.4), followed by alignment to the mm10 genome using STAR (v2.7.3a) with default settings. Only uniquely mapped reads were retained and filtered using Sambamba (v1.0.0). Mapping quality was assessed using RSeQC (v3.0.0). Raw counts were obtained using featureCounts (v2.0.6), whereas TPM and FPKM values were calculated using StringTie (v2.0). Differential gene expression analysis was performed using DESeq2 (v1.42.0), and GO analysis was conducted using clusterProfiler (v4.10.0).

Processed RNA-seq expression matrices and cell-type annotations were obtained from a published dataset. To reduce computational burden, up to 500 cells were randomly sampled from each annotated cell type, whereas all PGCs were retained. The published sci-RNA-seq dataset and Smart-seq2 data generated in this study were analyzed using Seurat (v4.3.0).^69^ Datasets from different platforms were integrated using an anchor-based strategy with reciprocal PCA (RPCA) following independent normalization and identification of highly variable genes. The integrated batch-corrected expression matrix was used for dimensionality reduction by PCA, followed by UMAP visualization based on the first 30 principal components. Pseudotime trajectory analysis was performed using Monocle3 (v1.0).^70^ Germ cell populations were extracted from the integrated dataset, and a Monocle3 object was constructed using the raw count matrix and corresponding metadata. Trajectory inference was performed following the standard Monocle3 workflow, with E8.5 wild-type PGCs specified as the root population representing the earliest developmental stage.

### Bulk RNA-seq and data analysis

E9.25 control and *Trim37*^*ko3*^ embryos carrying the *Nanog-EGFP* transgene were dissected from recipient mice and digested with 0.25% Trypsin-EDTA (Cat# 25200072; Thermo Fisher Scientific) at 37 °C. Following digestion, Nanog-EGFP-positive PGCs were isolated from the cell suspension by FACS. Control embryos were produced by injecting hA3A-eBE3-Y130F mRNA into the cytoplasm of zygotes from *Nanog-EGFP* males mated with BDF1 females. *Trim37*^*ko3*^ embryos were generated by co-injecting hA3A-eBE3-Y130F mRNA and *Trim37*-sgRNA-stop. Approximately 80 PGCs per sample were transferred into a 200-µL tube containing 3.5 µL of lysis buffer. The lysate was subjected to cDNA amplification using a Single Cell Full Length mRNA Amplification Kit (Cat# N712; Vazyme). RNA-seq libraries were prepared using a TruePrep DNA Library Prep Kit V2 (Cat# TD503; Vazyme) for Illumina sequencing. Sequencing was conducted on a NovaSeq 6000 at Genergy Biotechnology Co., Ltd (Shanghai, China). The raw RNA-seq data were first trimmed using TrimGalore (v0.6.10) to remove low-quality sequences and adapter contamination. Clean reads were then aligned to the mm10 reference genome using HISAT2 (v2.2.1). Gene-level counts were obtained using featureCounts (v2.0.1) based on UCSC GTF annotation, and FPKM values were calculated using Cufflinks (v2.2.1). Differential expression analysis was conducted in R using DESeq2 (v1.40.0), with genes considered differentially expressed if they had an adjusted *P*-value < 0.05 and a fold change > 2.

### ATAC-seq

Approximately 1500 PGCs per sample, isolated from E9.25 embryos following the aforementioned procedure, were used for miniATAC-seq as previously described.^71^ Libraries were sequenced on the NovaSeq 6000 platform at Genergy Biotechnology Co. Ltd (Shanghai, China).

### CUT&Tag

CUT&Tag was performed to detect H3K27ac (anti-H3K27ac antibody, Cat# ab4729; Abcam) and H3K9me3 (anti-H3K9me3 antibody, Cat# 39062; Active Motif, Carlsbad, CA, USA) modifications in the genomes of E9.25 control and *Trim37*^*ko3*^ PGCs. Approximately 1000 PGCs per sample were transferred into a 1.5-mL tube containing 500 µL of wash buffer. Samples were then processed, and libraries were constructed using a Hyperactive Universal CUT&Tag Assay Kit for Illumina Pro (Cat# TD904; Vazyme). Libraries were sequenced on the NovaSeq 6000 system at Genergy Biotechnology Co., Ltd (Shanghai, China).

### CUT&RUN

CUT&RUN was performed to detect TRIM28 or AP2γ binding in the genomes of E9.25 control and *Trim37*^*ko3*^ PGCs using anti-TRIM28 antibody (Cat# ab10483; Abcam) or an anti-AP2γ antibody (Cat# A7739; Abclonal, Wuhan, China), respectively. Approximately 3000 PGCs per sample were transferred into 1.5-mL tubes and resuspended in 60 µL of wash buffer (20 mM HEPES-KOH, pH 7.5, 150 mM NaCl, 0.5 mM spermidine, and protease inhibitors). Concanavalin A-coated magnetic beads (10 µL; Cat# 86057; Polysciences) were washed twice with binding buffer, resuspended, and added to the samples for 10 min at 23 °C with shaking. After placement on a magnetic stand to remove the buffer, samples were resuspended in antibody buffer (wash buffer with 0.02% digitonin and 2 mM EDTA) and incubated with the TRIM28 or AP2γ antibody overnight at 4 °C. The next day, samples were washed in dig wash buffer (wash buffer with 0.02% digitonin) and incubated in dig wash buffer containing pA-MNase (1:1000 dilution) for 1.5 h at 4 °C. After two washes, samples were resuspended in dig wash buffer, and 2 µL of 100 mM CaCl_2_ was added for digestion on ice for 25 min. To stop the reaction, stop buffer (340 mM NaCl, 20 mM EDTA, 4 mM EGTA, 50 μg/mL RNase A, 100 μg/mL glycogen, and 3 pg/mL *Drosophila* DNA) was added, followed by incubation for 3 min at 4 °C and for 39 min at 37 °C.

Following digestion, samples were centrifuged, and the supernatant was transferred into new tubes and treated with 2 μL of 10% SDS and proteinase K (20 mg/mL). Samples were vortexed, incubated for 10 min at 70 °C, and purified by phenol-chloroform extraction. DNA was precipitated with ethanol, washed, dried, and dissolved in 25 µL of water. For library construction, 3.5 µL of End Prep buffer, 1.5 µL of enzyme mix, and diluted adaptors were added, followed by ligation using reagents from the NEBNext Ultra II DNA Library Prep Kit. After ligation and USER enzyme treatment, samples were purified using AMPure beads, PCR amplified, and sequenced on a NovaSeq X Plus platform (Illumina).

### Data analysis of ATAC-seq, CUT&Tag, and CUT&RUN

Raw sequencing reads were trimmed using Trim Galore (v0.6.10) to remove adapter sequences and low-quality bases. The cleaned reads were then aligned to the mm10 reference genome using Bowtie2 (v2.5.1) with the following parameters: -N 1 -X 1000 --no-mixed --no-discordant. Unmapped reads, multiply mapped reads, and PCR duplicates were subsequently filtered out using Sambamba (v0.8.2). Peaks were called using MACS2 (v2.2.6) with the parameters -q 0.05 --nomodel --nolambda. To normalize read counts, RPKM (Reads Per Kilobase of transcript per Million mapped reads) values were calculated in 100-bp bins using bamCoverage from deepTools (v3.5.1). Differential binding peaks between control and *Trim37*^*ko3*^ samples were identified by pooling peaks across both groups and calculating the average *Z*-score-normalized RPKM values, with peaks showing a fold change ≥2 considered differentially bound. Finally, peaks were annotated to the nearest genes within 50 kb using the R package ChIPseeker.

## Supplementary information

Supplementary information, Fig. S1

Supplementary information, Fig. S2

Supplementary information, Fig. S3

Supplementary information, Fig. S4

Supplementary information, Fig. S5

Supplementary information, Fig. S6

Supplementary information, Fig. S7

Supplementary information, Fig. S8

Supplementary information, Fig. S9

Supplementary information, Fig. S10

Supplementary information, Fig. S11

Supplementary information, Fig. S12

Supplementary information, Fig. S13

Supplementary information, Fig. S14

Supplementary information, Fig. S15

Supplementary information, Fig. S16

Supplementary information, Table S1

Supplementary information, Table S2

Supplementary information, Table S3

Supplementary information, Table S4

Supplementary information, Table S5

Supplementary information, Table S6

## Data Availability

All sequencing data generated within this study have been uploaded to the NCBI Gene Expression Omnibus (GEO) and are available with accession codes: GSE289335 (RNA-seq), GSE289333 (ATAC-seq) and GSE289334 (CUT&Tag and CUT&RUN). Differentially expressed genes, ATAC-seq peaks, TRIM28 and AP2γ binding signals, and H3K27ac intensities are provided in Supplementary information, Table S6. The RNA-seq data of human PGCs were obtained from a previous study.^72^ All other data supporting the findings of this study are available from the corresponding author upon reasonable request.
