# Supplementary material for: TRIM37 interacts with TRIM28 to maintain primordial germ cell identity during migration
Source: Cell Res. 2026 Jul 30;36(10):785–803. doi: 10.1038/s41422-026-01272-2 (PMC13642344; doi:10.1038/s41422-026-01272-2)
Supplement: Supplementary file 1 [file 41422_2026_1272_MOESM10_ESM.pdf]

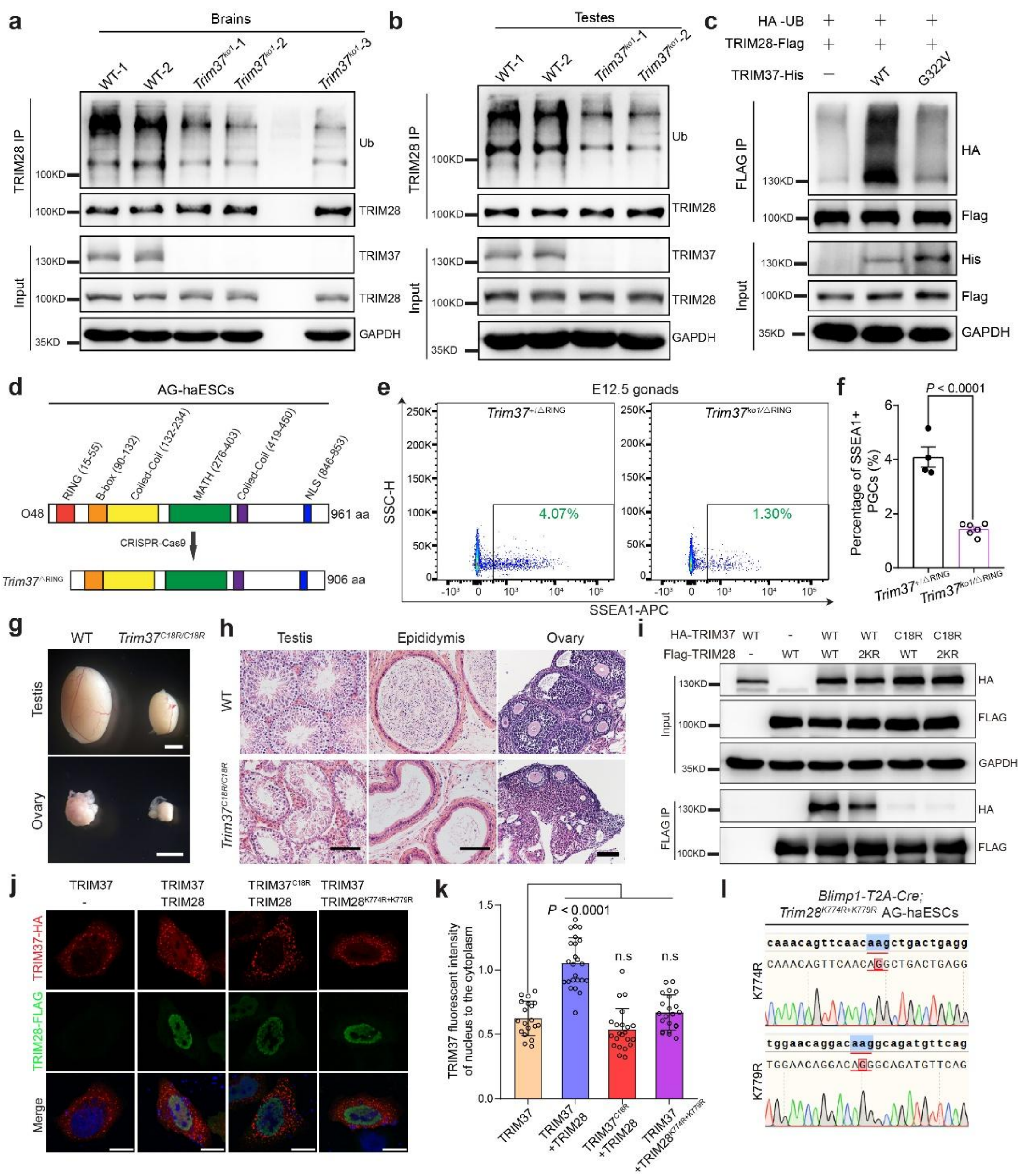

Fig. S10 The E3 ubiquitin ligase TRIM37 can ubiquitinate TRIM28 and regulate PGC development. a, **b** Immunoblot analysis of lysates obtained from the wild-type and *Trim37*^{ko1} brains (**a**) and testes (**b**). This step was followed by immunoprecipitation with an anti-TRIM28 affinity gel and analysis with anti-Ub, anti-TRIM28, anti-TRIM37, and anti-GAPDH antibodies. Two or three biological replicates for each group. **c** Immunoblot analysis of lysates obtained from the HEK293T cells transfected with Flag-tagged TRIM28, HA-tagged Ub, and His-tagged TRIM37 or TRIM37^{G322V}. This step was followed by immunoprecipitation with an anti-Flag affinity gel and analysis with anti-HA, anti-Flag, anti-His, and anti-GAPDH antibodies. **d** Schematic illustration of generating *Trim37*$^{\triangle RING}$ AG-haESCs *via* CRISPR-Cas9 technology. **e** Representative FACS results showing SSEA1 positive PGCs from E12.5 *Trim37*$^{+/\triangle RING}$ and *Trim37*$^{ko1/\triangle RING}$ gonads. **f** Boxplots showing the ratio of SSEA1-APC positive PGCs in E12.5 gonads (n = 4 embryos for *Trim37*$^{+/\triangle RING}$ and n = 6 embryos *Trim37*$^{ko1/\triangle RING}$). Data are presented as mean ± s.e.m., with each circle representing an individual embryo. *P*-values were determined using an unpaired two-sided Student's

t-test. **g** Morphology analysis showed testis and ovary from *Trim37*$^{C18R/C18R}$ mice were severely atrophied. Scale bars, 2 mm. **h** Histological analysis of the testis, epididymis, and ovary of the control and *Trim37*$^{C18R/C18R}$ mice showed abnormal germ cell development in the mutant organs. Three adult mice (8 weeks of age) were used for each group. Scale bars, 100 μm. **i** Immunoblot analysis was performed on lysates from HEK293T cells transfected with Flag-tagged TRIM28, HA-tagged TRIM37, and their mutants TRIM28$^{K774R+K779R}$ (2KR) and TRIM37^{C18R}. Immunoprecipitation was carried out using anti-Flag affinity gel, followed by immunoblotting with anti-Flag, anti-HA, and anti-GAPDH antibodies. **j** Confocal microscopy of the HeLa cells transfected with wild-type HA-tagged TRIM37 and C18R mutant, and Flag-tagged wild-type TRIM28 and K774R+K779R mutant. Nuclei were stained with Hoechst 33342 (blue). Scale bars, 10 μm. **k** Boxplots showing the ratio of fluorescent intensity of wild-type TRIM37-HA and C18R mutant in the nucleus to the cytoplasm related to **j**. Data are presented as mean ± s.e.m., and *P*-values were determined using an unpaired two-sided Student's t-test. **l** Sanger sequencing confirmed the successful introduction of K774R and K779R mutations into the genome of the *Blimp1-T2A-Cre; Trim28*$^{K774R+K779R}$ AG-haESC line.
