# Supplementary material for: TRIM37 interacts with TRIM28 to maintain primordial germ cell identity during migration
Source: Cell Res. 2026 Jul 30;36(10):785–803. doi: 10.1038/s41422-026-01272-2 (PMC13642344; doi:10.1038/s41422-026-01272-2)
Supplement: Supplementary file 2 [file 41422_2026_1272_MOESM11_ESM.pdf]

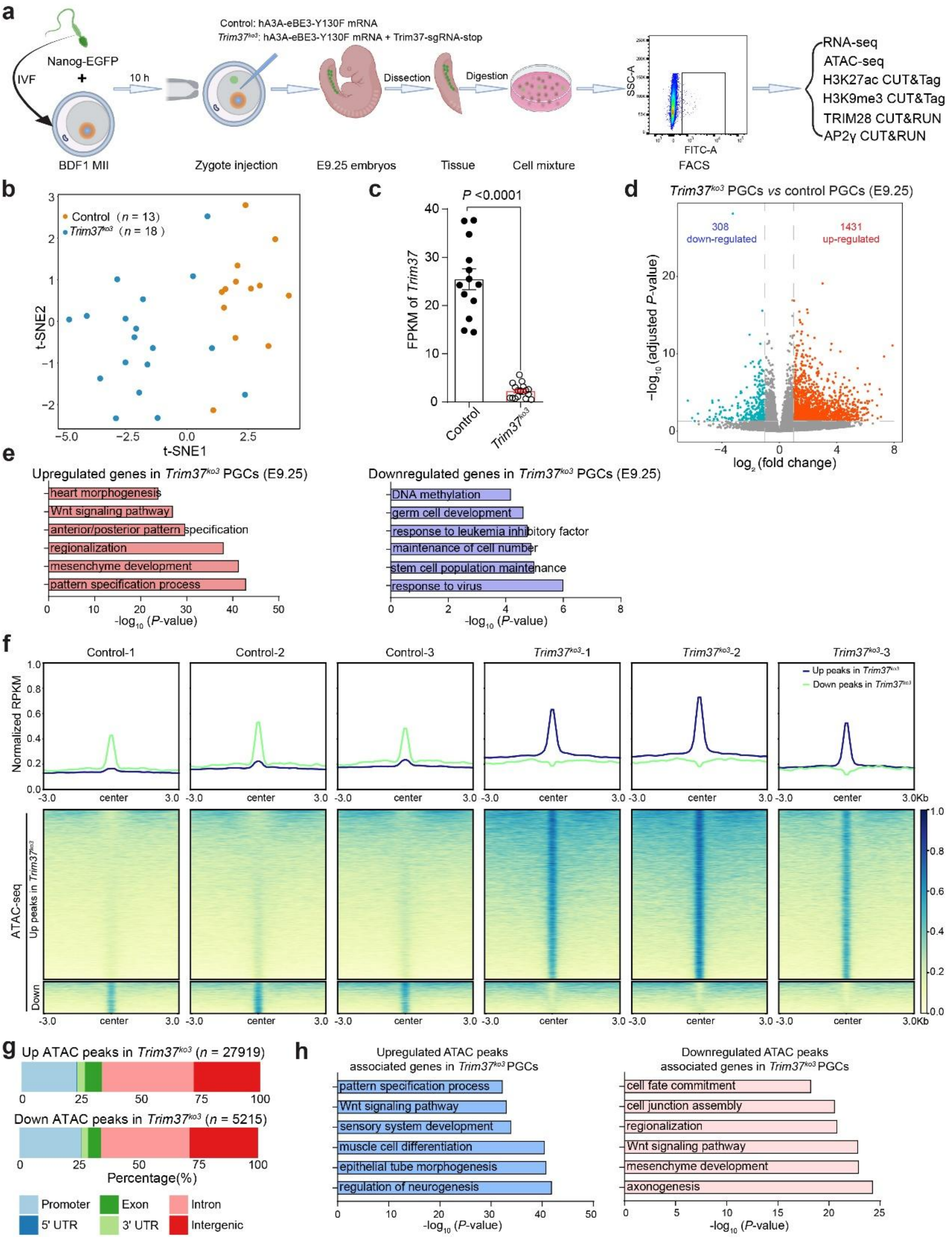

Fig. S11 *Trim37* deficiency caused upregulation of somatic genes and increased their chromatin accessibility in PGCs. a Schematic diagram of collecting *Trim37*^{ko3} PGCs from E9.25 embryos carrying *Nanog-EGFP* transgene for RNA-seq, ATAC-seq, CUT&Tag, and CUT&RUN. **b** t-SNE analysis and cluster allocation for the bulk RNA-seq data from control and *Trim37*^{ko3} PGCs at E9.25. *n* indicated the number of samples. Each sample includes about 50 PGCs with *Nanog-EGFP* enriched by FACS. **c** Bar plot showing the expression level of *Trim37* in the control and *Trim37*^{ko3} PGCs based on RNA-seq data. Data are shown as the average mean ± s.e.m. The *P*-value was calculated by Student's unpaired two-sided t-test. **d** Volcano plots show differentially expressed genes between *Trim37*^{ko3} and control PGCs at E9.25 (adjusted *P*-value < 0.05, FC > 2). **e** Enriched

GO terms of significantly upregulated and downregulated genes in *Trim37*^{ko3} PGCs compared to wild-type at E9.25. **f** Line plots and heatmaps show altered ATAC-seq peaks within 3 kb from the center in control and *Trim37*^{ko3} PGCs. **g** Enrichment plots display genomic features of up- and down-regulated ATAC-seq peaks in *Trim37*^{ko3} PGCs. **h** The GO terms analysis of genes associated with up- and down-regulated ATAC-seq peaks in *Trim37*^{ko3} PGCs.
