# Supplementary material for: TRIM37 interacts with TRIM28 to maintain primordial germ cell identity during migration
Source: Cell Res. 2026 Jul 30;36(10):785–803. doi: 10.1038/s41422-026-01272-2 (PMC13642344; doi:10.1038/s41422-026-01272-2)
Supplement: Supplementary file 3 [file 41422_2026_1272_MOESM12_ESM.pdf]

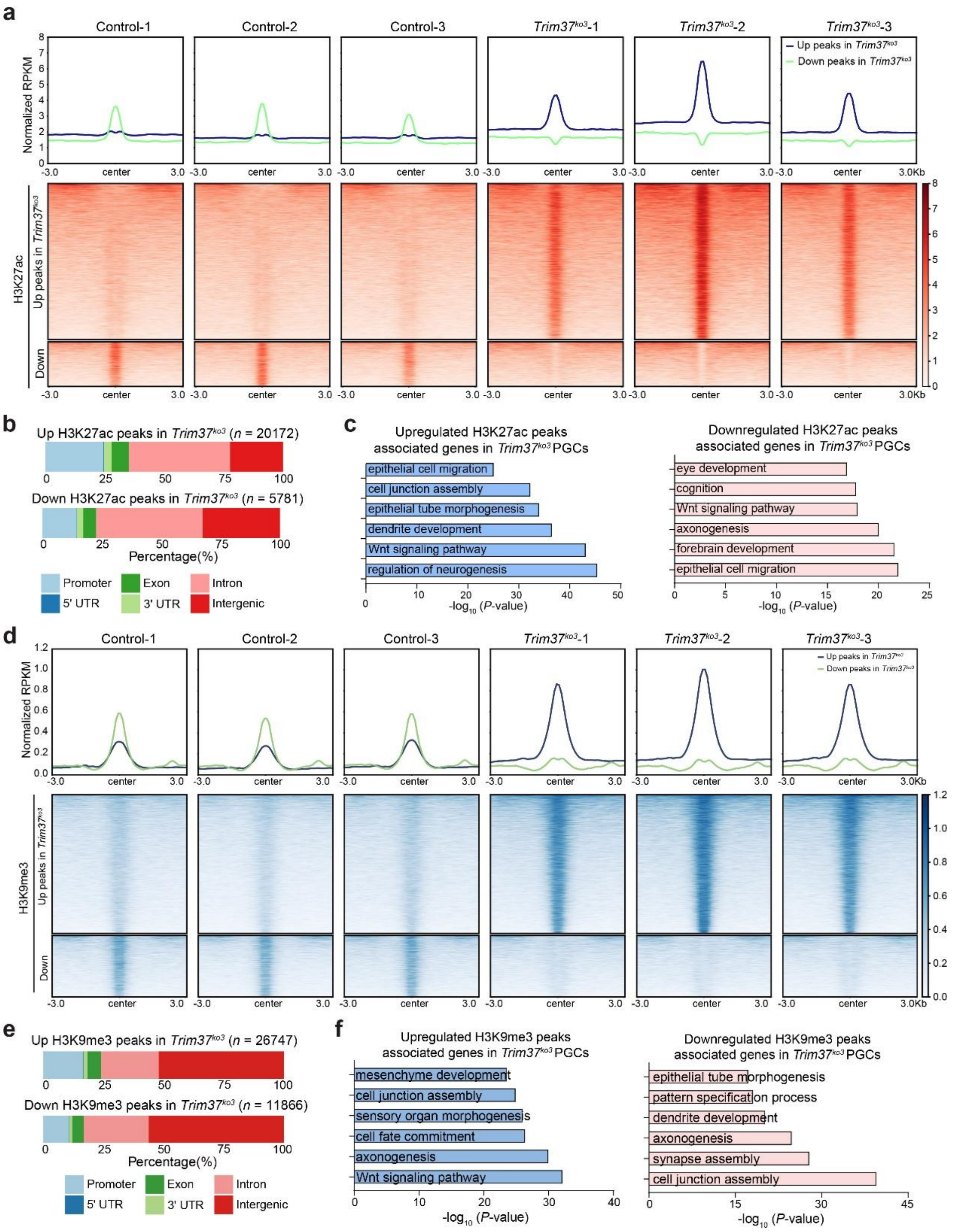

Fig. S12 Open chromatin regions in *Trim37* knockout PGCs are associated with increased H3K27ac modification. **a** Line plots and heatmaps show altered H3K27ac CUT&Tag peaks within 3 kb from the center in control and *Trim37^{ko3}* PGCs. **b** Enrichment plots display genomic features of up- and down-regulated H3K27ac CUT&Tag peaks in *Trim37^{ko3}* PGCs. **c** The GO terms analysis of genes associated with up- and down-regulated H3K27ac CUT&Tag peaks in *Trim37^{ko3}* PGCs. **d** Line plots and heatmaps show altered H3K9me3 CUT&Tag peaks within 3 kb from the center in control and *Trim37^{ko3}* PGCs. **e** Enrichment plots display genomic features of up- and down-regulated H3K9me3 CUT&Tag peaks in *Trim37^{ko3}* PGCs. **f** The GO terms analysis of genes associated with up- and down-regulated H3K9me3 CUT&Tag peaks in *Trim37^{ko3}* PGCs.
