# Supplementary material for: TRIM37 interacts with TRIM28 to maintain primordial germ cell identity during migration
Source: Cell Res. 2026 Jul 30;36(10):785–803. doi: 10.1038/s41422-026-01272-2 (PMC13642344; doi:10.1038/s41422-026-01272-2)
Supplement: Supplementary file 4 [file 41422_2026_1272_MOESM13_ESM.pdf]

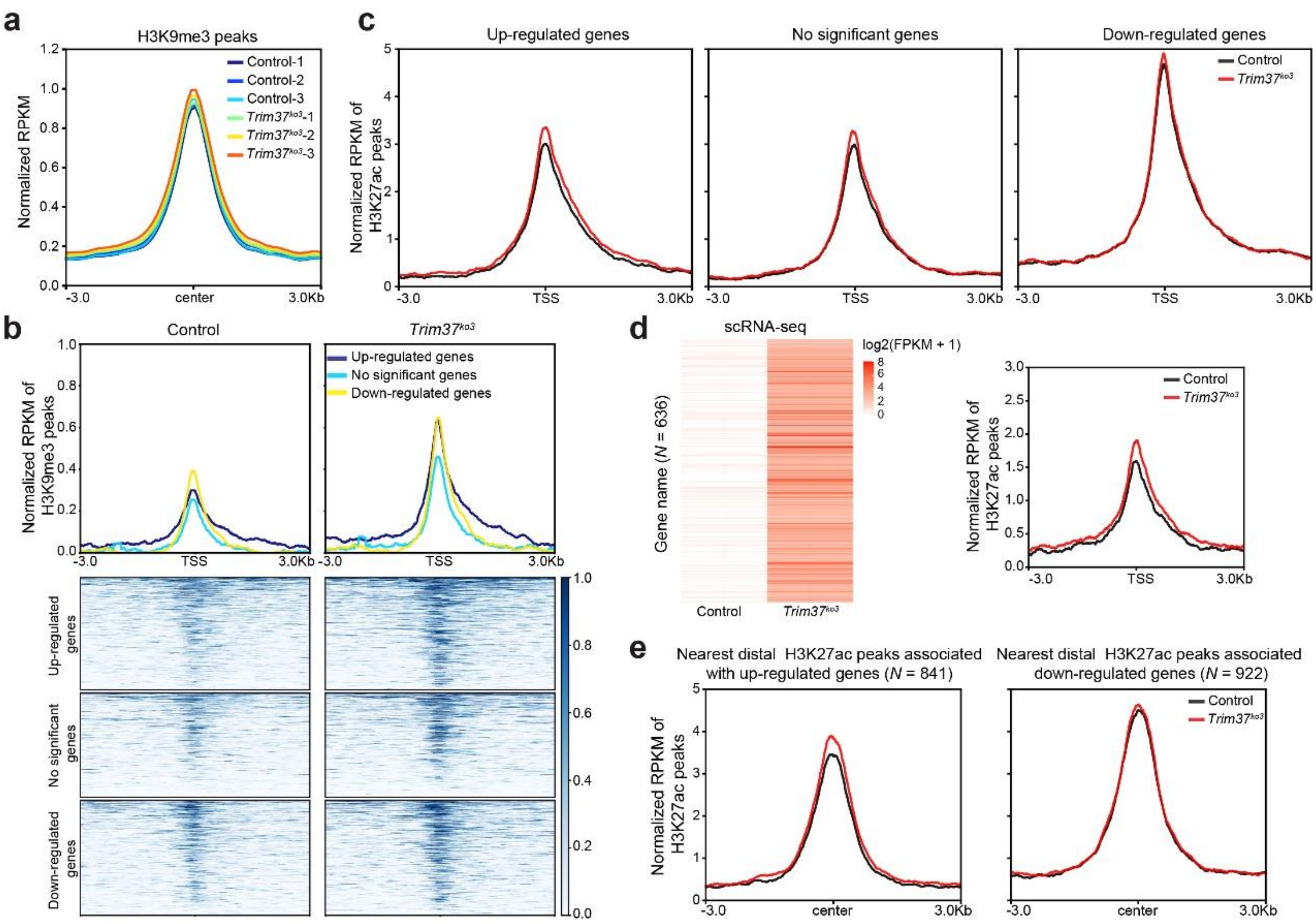

Fig. S13 *Trim37* deficiency is associated with altered H3K27ac landscapes at transcriptionally dysregulated genes in PGCs. a Average profiles of H3K9me3 CUT&Tag signals centered on H3K9me3 peaks in control and *Trim37^{ko3}* PGCs. **b** Heatmaps and average profiles showing H3K9me3 distribution at promoter regions (transcription start site ± 3 kb) of upregulated, downregulated, and non-significantly changed genes in control and *Trim37^{ko3}* PGCs. **c** Average profiles showing H3K27ac enrichment at promoter regions of upregulated, downregulated, and non-significantly changed genes in control and *Trim37^{ko3}* PGCs. **d** Left, heatmap showing the expression of activated genes in control and *Trim37^{ko3}* PGCs. Average FPKM values were calculated from merged datasets for each genotype. Genes with average FPKM ⩾ 5 in *Trim37^{ko3}* PGCs and ⩽ 1 in control PGCs were defined as activated genes. Right, H3K27ac enrichment at promoter regions of activated genes in control and *Trim37^{ko3}* PGCs. **e** Average H3K27ac signal profiles at the nearest distal H3K27ac peaks associated with upregulated or downregulated genes in control and *Trim37^{ko3}* PGCs. For each gene, the nearest distal H3K27ac peak was selected for downstream analysis.
