# Supplementary material for: TRIM37 interacts with TRIM28 to maintain primordial germ cell identity during migration
Source: Cell Res. 2026 Jul 30;36(10):785–803. doi: 10.1038/s41422-026-01272-2 (PMC13642344; doi:10.1038/s41422-026-01272-2)
Supplement: Supplementary file 5 [file 41422_2026_1272_MOESM14_ESM.pdf]

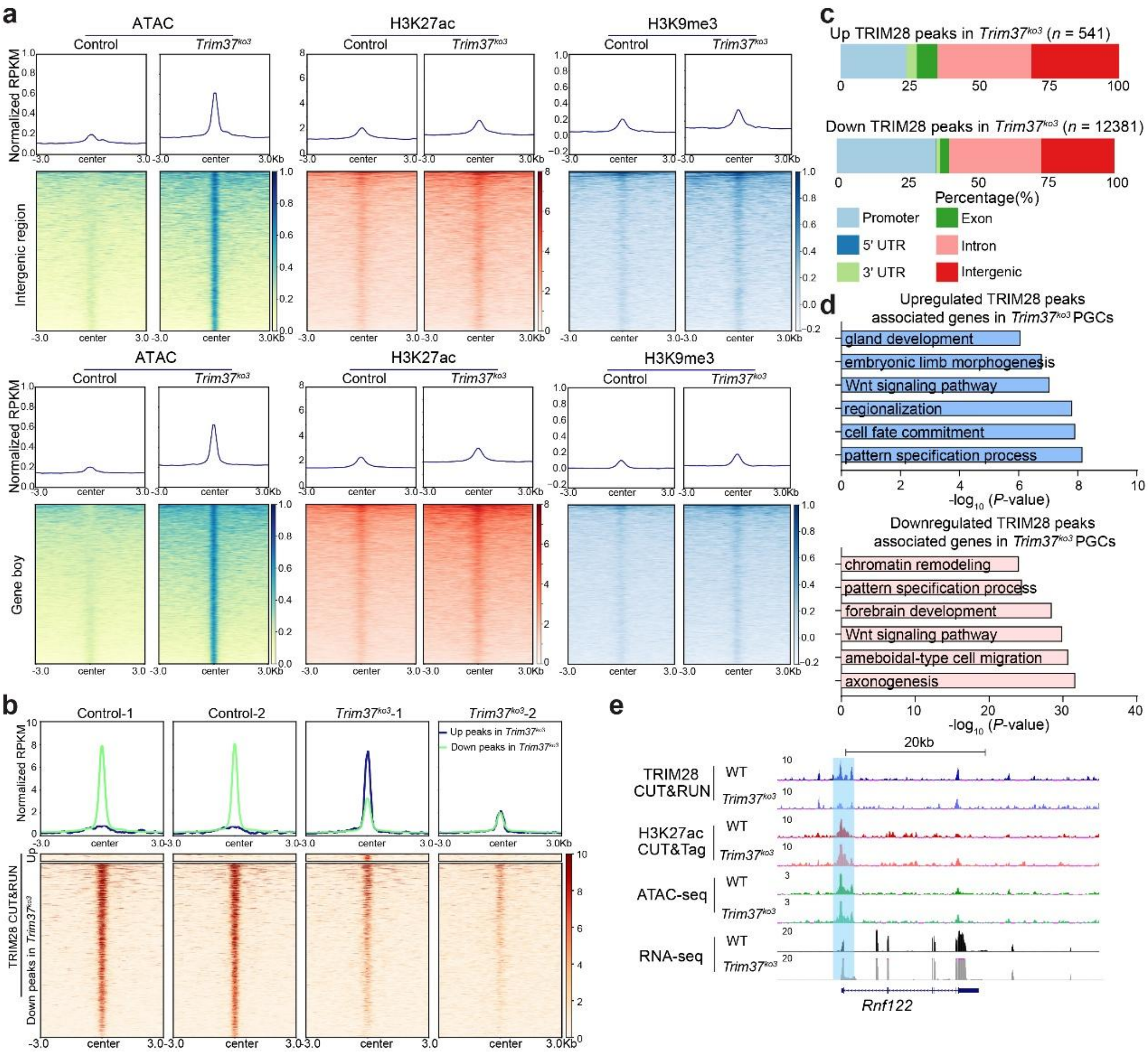

Fig. S14 TRIM37 deficiency alters the binding capacity of TRIM28 in PGCs. a Line plots and heatmaps display ATAC, H3K27ac, and H3K9me3 signals in control and *Trim37*^{ko3} PGCs at intergenic and gene body regions, with upregulated peaks identified from ATAC-seq data. **b** Line plots and heatmaps show altered TRIM28 CUT&RUN peaks within 3 kb from the center in control and *Trim37*^{ko3} PGCs. **c** Enrichment plots display genomic features of up- and down-regulated TRIM28 CUT&RUN peaks in *Trim37*^{ko3} PGCs. **d** The GO terms analysis of genes associated with up- and down-regulated TRIM28 CUT&RUN peaks in *Trim37*^{ko3} PGCs. **e** UCSC browser views displayed the signals of RNA-seq, TRIM28 CUT&RUN, H3K27ac CUT&Tag, and ATAC-seq for *Rnf122* in *Trim37*^{ko3} PGCs, with red-shaded boxes highlighting the open chromatin regions.
