# Supplementary material for: TRIM37 interacts with TRIM28 to maintain primordial germ cell identity during migration
Source: Cell Res. 2026 Jul 30;36(10):785–803. doi: 10.1038/s41422-026-01272-2 (PMC13642344; doi:10.1038/s41422-026-01272-2)
Supplement: Supplementary file 6 [file 41422_2026_1272_MOESM15_ESM.pdf]

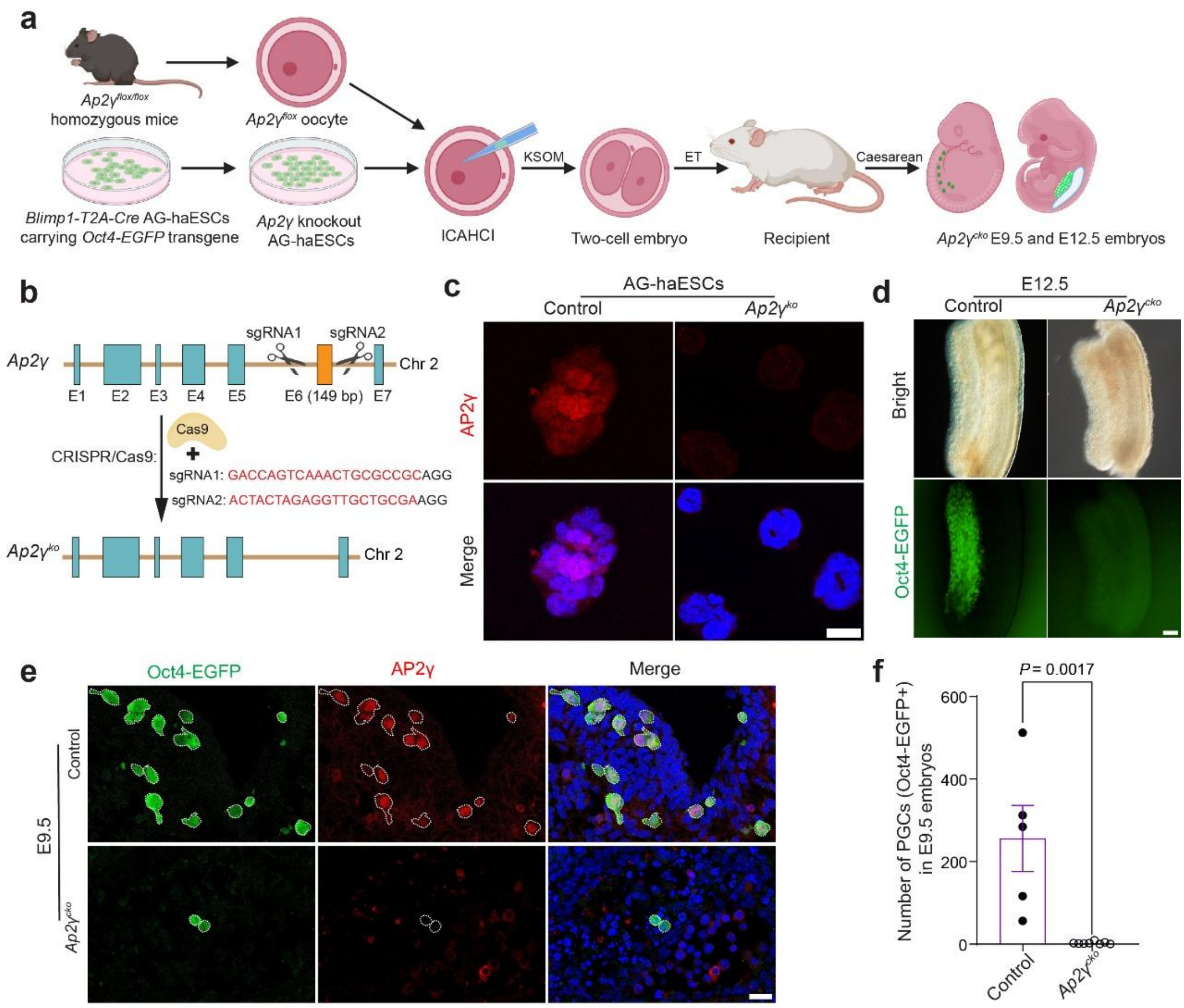

Fig. S15 *Blimp1-Cre*–mediated deletion of *Ap2γ* leads to PGC depletion from E9.5. a Schematic of the experimental strategy for rapid generation of *Ap2γ* conditional knockout embryos using semi-cloning technology to assess PGC development in one step. *Ap2γ*-inactivated AG-haESCs, generated by CRISPR-Cas9 based on *Blimp1-Cre* AG-haESCs carrying the *Oct4-EGFP* transgene, were injected into $Ap2\gamma^{flox}$ oocytes to produce conditional knockout embryos. **b** Schematic diagram of the generation of $Ap2\gamma^{ko}$ AG-haESCs using CRISPR-Cas9. Red bases indicate the sgRNA target sequence. **c** Confocal images showing AP2γ expression in control and $Ap2\gamma^{ko}$ AG-haESCs. Scale bar, 20 µm. **d** Representative images of gonads in E12.5 semi-cloned embryos (control and $Ap2\gamma^{cko}$). Green fluorescence indicates the expression of Oct4-EGFP, showing the location of PGCs. Two embryos were used for each group. Scale bars, 100 µm. **e** Immunofluorescence of E9.5 control and $Ap2\gamma^{cko}$ embryos evaluating OCT4-EGFP positive PGCs (green), AP2γ (red), and DAPI (blue). AP2γ specifically disappeared in the PGCs of the $Ap2\gamma^{cko}$ embryo. Scale bars, 20 µm. **f** Boxplots showing the number of PGCs with OCT4-EGFP signals in E9.5 embryos (n = 5 for control and n = *8* for $Ap2\gamma^{cko}$). Data are presented as mean ± s.e.m., with each circle representing an individual embryo. *P*-values were determined using an unpaired two-sided Student's t-test.
