# Supplementary material for: TRIM37 interacts with TRIM28 to maintain primordial germ cell identity during migration
Source: Cell Res. 2026 Jul 30;36(10):785–803. doi: 10.1038/s41422-026-01272-2 (PMC13642344; doi:10.1038/s41422-026-01272-2)
Supplement: Supplementary file 7 [file 41422_2026_1272_MOESM16_ESM.pdf]

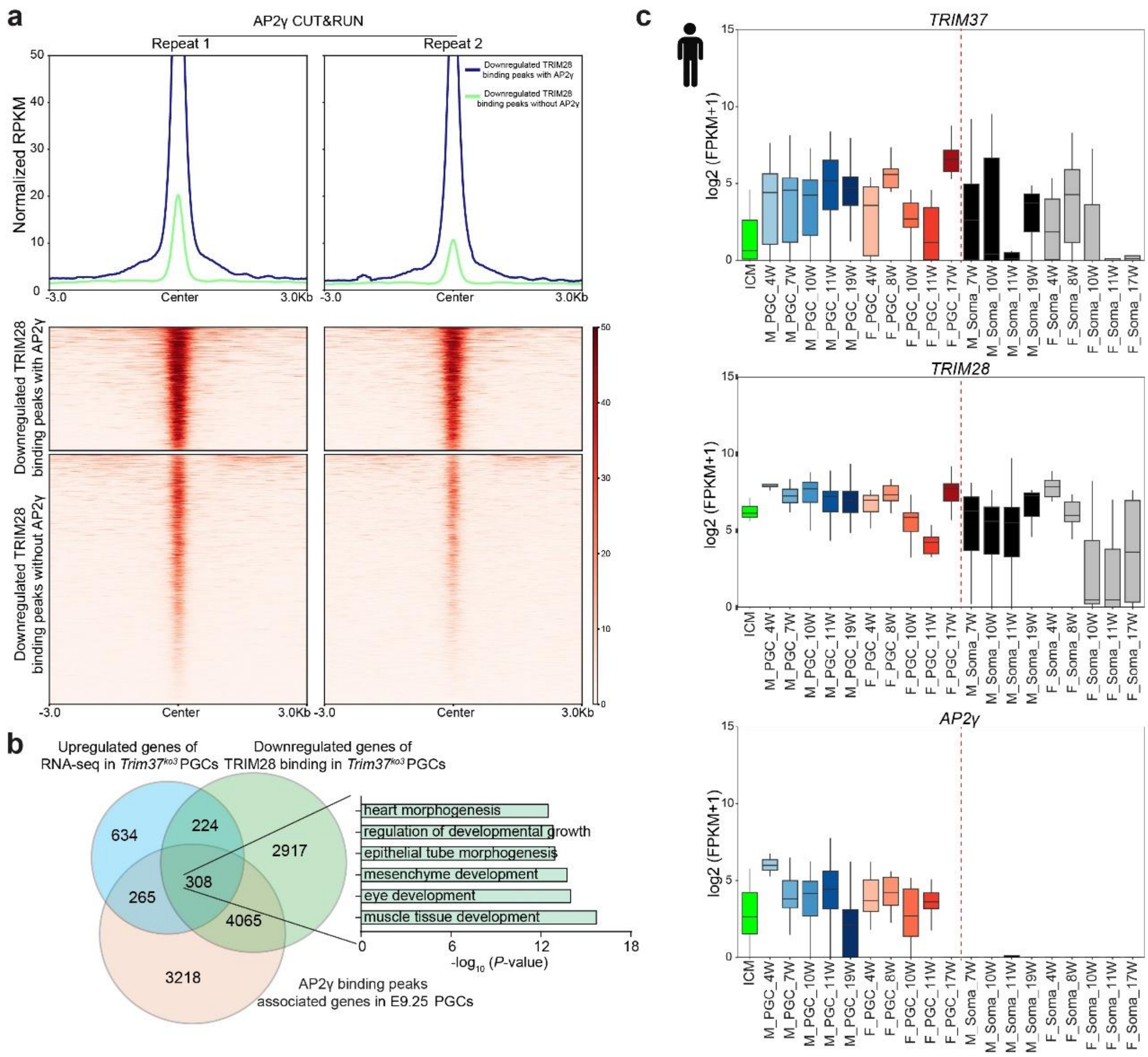

Fig. S16 AP2γ may help the TRIM37-TRIM28 complex suppress somatic genes in PGCs. a Line plots and heatmaps show AP2γ CUT&RUN peaks associated with TRIM28 downregulated binding peaks within 3 kb from the center in E9.25 wild-type PGCs. **b** Venn diagram showed the overlap between upregulated genes from RNA-seq, downregulated peaks associated genes from TRIM28 CUT&RUN in *Trim37*^{ko3} PGCs, and AP2γ CUT&RUN peaks associated genes from wild-type PGCs. The GO terms for the overlapping genes are presented in the right panel. **c** The expression levels of *TRIM37*, *TRIM28*, and *AP2γ* at different developmental stages of human PGCs were obtained from a previous study [72].
