# Supplementary material for: TRIM37 interacts with TRIM28 to maintain primordial germ cell identity during migration
Source: Cell Res. 2026 Jul 30;36(10):785–803. doi: 10.1038/s41422-026-01272-2 (PMC13642344; doi:10.1038/s41422-026-01272-2)
Supplement: Supplementary file 8 [file 41422_2026_1272_MOESM17_ESM.pdf]

Table S1. Expression of Trim family genes in E9.5 PGCs.

Gene names	WT-E9.5-1	WT-E9.5-2	Average FPKM	Trim library
Trim28	103.561	101.216	102.3885	Trim-library-1
Trim59	57.7108	68.6991	63.20495	Trim-library-1
Trim13	53.3083	47.8572	50.58275	Trim-library-1
Trim27	32.2956	32.7027	32.49915	Trim-library-1
Trim25	39.614	21.347	30.4805	Trim-library-1
Trim37	36.1982	22.7155	29.45685	Trim-library-1
Trim6	29.8951	19.7972	24.84615	Trim-library-1
Trim26	30.2616	8.03953	19.150565	Trim-library-1
Trim35	8.62539	20.5174	14.571395	Trim-library-1
Trim12a	11.9404	11.5814	11.7609	Trim-library-1
Trim23	4.07722	12.0143	8.04576	Trim-library-1
Trim17	4.5377	8.444	6.49085	Trim-library-1
Trim11	6.97249	5.01959	5.99604	Trim-library-1
Trim24	3.87815	2.02699	2.95257	Trim-library-1
Trim12c	3.93535	1.21779	2.57657	Trim-library-1
Trim71	12.1087	10.2664	11.18755	Trim-library-2
Trim30a	9.3117	9.3418	9.32675	Trim-library-2
Trim33	7.68013	6.48119	7.08066	Trim-library-2
Trim44	6.46308	4.65693	5.560005	Trim-library-2
Trim39	4.72744	3.98293	4.355185	Trim-library-2
Trim8	6.47346	2.13156	4.30251	Trim-library-2
Trim32	3.58948	4.75533	4.172405	Trim-library-2
Trim34a	4.16633	3.38564	3.775985	Trim-library-2
Trim68	3.68073	2.67524	3.177985	Trim-library-2
Trim41	1.11923	3.74182	2.430525	Trim-library-2
Trim36	1.40678	1.7767	1.59174	Trim-library-2
Triml2	0	2.96609	1.483045	Trim-library-2
Trim45	1.18661	1.61573	1.40117	Trim-library-2
Trim3	0.680152	1.66052	1.170336	Trim-library-2
Trim34b	1.3434	0.918	1.1307	Trim-library-2
Trim2	1.11188	0.819304	0.965592	Trim-library-2
Trim56	0.541035	0.925867	0.733451	Trim-library-2
Trim7	0.985446	0.363414	0.67443	Trim-library-2
Trim46	0.500433	0.0242238	0.2623284	Other Trims
Triml1	0	0.221187	0.1105935	Other Trims
Trim43b	0.204202	0	0.102101	Other Trims
Trim16	0	0.0331279	0.01656395	Other Trims
Trim47	0	0.0305737	0.01528685	Other Trims
Trim14	0	0	0	Other Trims

Trim38	0	0	0	Other Trims
Trim69	0	0	0	Other Trims
Trim54	0	0	0	Other Trims
Trim62	0	0	0	Other Trims
Trim63	0	0	0	Other Trims
Trim52	0	0	0	Other Trims
Trim67	0	0	0	Other Trims
Trim21	0	0	0	Other Trims
Trim30b	0	0	0	Other Trims
Trim65	0	0	0	Other Trims
Trim10	0	0	0	Other Trims
Trim15	0	0	0	Other Trims
Trim29	0	0	0	Other Trims
Trim30d	0	0	0	Other Trims
Trim30e-ps1	0	0	0	Other Trims
Trim31	0	0	0	Other Trims
Trim40	0	0	0	Other Trims
Trim42	0	0	0	Other Trims
Trim43a	0	0	0	Other Trims
Trim43c	0	0	0	Other Trims
Trim50	0	0	0	Other Trims
Trim55	0	0	0	Other Trims
Trim58	0	0	0	Other Trims
Trim60	0	0	0	Other Trims
Trim61	0	0	0	Other Trims
Trim66	0	0	0	Other Trims
Trim72	0	0	0	Other Trims
Trim75	0	0	0	Other Trims
Trim9	0	0	0	Other Trims
