# Supplementary material for: TRIM37 interacts with TRIM28 to maintain primordial germ cell identity during migration
Source: Cell Res. 2026 Jul 30;36(10):785–803. doi: 10.1038/s41422-026-01272-2 (PMC13642344; doi:10.1038/s41422-026-01272-2)
Supplement: Supplementary file 9 [file 41422_2026_1272_MOESM18_ESM.pdf]

Table S2. Lists of sgRNA sequences of the Trim-library.

Library	sgRNAs	Forward sequence	Reversed sequence
Trim-library-1	Trim11-sgRNA-1	CACCGAGTGCATCCGACGTTGCTGGT	TAAAACCAGCAACGTCGGATGCACTC
Trim-library-1	Trim11-sgRNA-2	CACCGCGCCTTCTCCGGTCCCGCAGT	TAAAACTGCGGGACCGGAGAAGGCGC
Trim-library-1	Trim11-sgRNA-3	CACCGGCCGACAGCTCGCGACACTGT	TAAAACAGTGTCGCGAGCTGTCGGCC
Trim-library-1	Trim12a-sgRNA-1	CACCGGCTAAACTGGTAACTAATTGT	TAAAACAATTAGTTACCAGTTTAGCC
Trim-library-1	Trim12a-sgRNA-2	CACCGGGTAACTAATTCGGCACACGT	TAAAACGTGTGCCGAATTAGTTACCC
Trim-library-1	Trim12a-sgRNA-3	CACCGGTAACTAATTCGGCACACAGT	TAAAACTGTGTGCCGAATTAGTTACC
Trim-library-1	Trim12c-sgRNA-1	CACCGGAAAACATTATCAACAAACGT	TAAAACGTTTGTTGATAATGTTTTCC
Trim-library-1	Trim12c-sgRNA-2	CACCGAACATTTCCTTATCGCATTGT	TAAAACAATGCGATAAGGAAATGTTC
Trim-library-1	Trim12c-sgRNA-3	CACCGTTCAAGCCTAGCCCAGAAGGT	TAAAACCTTCTGGGCTAGGCTTGAAC
Trim-library-1	Trim13-sgRNA-1	CACCGTACTCCCTAAAGGGTATCGGT	TAAAACCGATACCCTTTAGGGAGTAC
Trim-library-1	Trim13-sgRNA-2	CACCGGGAGATGCTCTTTCCCGCTGT	TAAAACAGCGGGAAAGAGCATCTCCC
Trim-library-1	Trim13-sgRNA-3	CACCGGCCCTCCAGTTACTCACGAGT	TAAAACTCGTGAGTAACTGGAGGGCC
Trim-library-1	Trim17-sgRNA-1	CACCGCCAGGAGCACCGGATGCACGT	TAAAACGTGCATCCGGTGCTCCTGGC
Trim-library-1	Trim17-sgRNA-2	CACCGCAGGAGCACCGGATGCACAGT	TAAAACTGTGCATCCGGTGCTCCTGC
Trim-library-1	Trim17-sgRNA-3	CACCGCTTTTGTCAGGAGGCGGTTGT	TAAAACAACCGCCTCCTGACAAAAGC
Trim-library-1	Trim23-sgRNA-1	CACCGTGTTACTTGTCGATCAAATGT	TAAAACATTTGATCGACAAGTAACAC
Trim-library-1	Trim23-sgRNA-2	CACCGCTGTTACTTGTCGATCAAAGT	TAAAACTTTGATCGACAAGTAACAGC
Trim-library-1	Trim23-sgRNA-3	CACCGTCTAGGAGACTCAGGTGTAGT	TAAAACTACACCTGAGTCTCCTAGAC
Trim-library-1	Trim24-sgRNA-1	CACCGGAGCGGCGGCGAGGCGTCCGT	TAAAACGGACGCCTCGCCGCCGCTCC
Trim-library-1	Trim24-sgRNA-2	CACCGTGGCGAAGGACCACCGGCCGT	TAAAACGGCCGGTGGTCCTTCGCCAC

Trim-library-1	Trim24-sgRNA-3	CACCGCTTGGGTGGCGAATGGCGAGT	TAAAACTCGCCATTCGCCACCCAAGC
Trim-library-1	Trim25-sgRNA-1	CACCGAAGAACACGGTAATGTGCGGT	TAAAACCGCACATTACCGTGTTCTTC
Trim-library-1	Trim25-sgRNA-2	CACCGAGGCCTCACTTCGACAGTCGT	TAAAACGACTGTCGAAGTGAGGCCTC
Trim-library-1	Trim25-sgRNA-3	CACCGAGTCAAGCCAGCGCCGACCGT	TAAAACGGTCGGCGCTGGCTTGACTC
Trim-library-1	Trim26-sgRNA-1	CACCGAGTGATATCCGCCCCATATGT	TAAAACATATGGGGCGGATATCACTC
Trim-library-1	Trim26-sgRNA-2	CACCGTGATATCCGCCCCATATCGGT	TAAAACCGATATGGGGCGGATATCAC
Trim-library-1	Trim26-sgRNA-3	CACCGCCGCCCCATATCGGGGAACGT	TAAAACGTTCCCCGATATGGGGCGGC
Trim-library-1	Trim27-sgRNA-1	CACCGGCGCCACATGCGACCCAACGT	TAAAACGTTGGGTCGCATGTGGCGCC
Trim-library-1	Trim27-sgRNA-2	CACCGACCGCTCGCGCGAGCACCGGT	TAAAACCGGTGCTCGCGCGAGCGGTC
Trim-library-1	Trim27-sgRNA-3	CACCGACACGCCCATCTCGCCGCCGT	TAAAACGGCGGCGAGATGGGCGTGTC
Trim-library-1	Trim28-sgRNA-1	CACCGGCAGCCGCGTCGTCCCCTGGT	TAAAACCAGGGGACGACGCGGCTGCC
Trim-library-1	Trim28-sgRNA-2	CACCGCCGCCGCAGCGAATAATTCGT	TAAAACGAATTATTCGCTGCGGCGGC
Trim-library-1	Trim28-sgRNA-3	CACCGCCGCCGCAGCGAATAATTCGT	TAAAACGAATTATTCGCTGCGGCGGC
Trim-library-1	Trim35-sgRNA-1	CACCGCAGACGACGCCCTCGTGTCGT	TAAAACGACACGAGGGCGTCGTCTGC
Trim-library-1	Trim35-sgRNA-2	CACCGAAGGAACGAGCGGTGCCCGGT	TAAAACCGGGCACCGCTCGTTCCTTC
Trim-library-1	Trim35-sgRNA-3	CACCGGACACTGCGCAAGACTTCCGT	TAAAACGGAAGTCTTGCGCAGTGTCC
Trim-library-1	Trim37-sgRNA-1	CACCGCCACTCCAGCTACGGGAACGT	TAAAACGTTCCCGTAGCTGGAGTGGC
Trim-library-1	Trim37-sgRNA-2	CACCGTCTGCTCATCCATTGCCTCGT	TAAAACGAGGCAATGGATGAGCAGAC
Trim-library-1	Trim37-sgRNA-3	CACCGATGAGACGTCGACGAAGTTGT	TAAAACAACTTCGTCGACGTCTCATC
Trim-library-1	Trim59-sgRNA-1	CACCGAATTAGCTCCTTAAAATACGT	TAAAACGTATTTTAAGGAGCTAATTC
Trim-library-1	Trim59-sgRNA-2	CACCGATGGTTGCCTGTAGTGTTCGT	TAAAACGAACACTACAGGCAACCATC
Trim-	Trim59-	CACCGGTTGTTCTCCAGTATT	TAAAACTAAAATACTGGAG

library-1	sgRNA-3	TTAGT	AACAACC
Trim-library-1	Trim6-sgRNA-1	CACC**G**TGGGAACCTCCGTCCTAAT**GT**	TAAAA**C**ATTAGGACGGAGGTTCCC**AC**
Trim-library-1	Trim6-sgRNA-2	CACCGCTCCGTCCTAATCGGCACCGT	TAAAACGGTGCCGATTAGGACGGAGC
Trim-library-1	Trim6-sgRNA-3	CACCGGATAGGAGGTCCGGCACACGT	TAAAACGTGTGCCGGACCTCCTATCC
Trim-library-2	Trim2-sgRNA-1	CACCGCCAGCTGGTCGTTCTCGCGGT	TAAAACCGCGAGAACGACCAGCTGGC
Trim-library-2	Trim2-sgRNA-2	CACCGACCATCCTCACCACTAACGGT	TAAAACCGTTAGTGGTGAGGATGGTC
Trim-library-2	Trim2-sgRNA-3	CACCGATTTTATCGTGGAAACGGAGT	TAAAACTCCGTTTCCACGATAAAATC
Trim-library-2	Trim30a-sgRNA-1	CACCGATGGATATCCAGCAATACTGT	TAAAACAGTATTGCTGGATATCCATC
Trim-library-2	Trim30a-sgRNA-2	CACCGCATGGATATCCAGCAATACGT	TAAAACGTATTGCTGGATATCCATGC
Trim-library-2	Trim30a-sgRNA-3	CACCGGGTAAGGAACTCGGCATACGT	TAAAACGTATGCCGAGTTCCTTACCC
Trim-library-2	Trim32-sgRNA-1	CACCGGGAGAAGTTGACTCGTCTAGT	TAAAACTAGACGAGTCAACTTCTCCC
Trim-library-2	Trim32-sgRNA-2	CACCGACACTGGCGGCAGATGGTAGT	TAAAACTACCATCTGCCGCCAGTGTC
Trim-library-2	Trim32-sgRNA-3	CACCGTTCAGCACCGTCAGGTTGTGT	TAAAACACAACCTGACGGTGCTGAAC
Trim-library-2	Trim33-sgRNA-1	CACCGGGCTGGGCCCGACGACGGGGT	TAAAACCCCGTCGTCGGGCCCAGCCC
Trim-library-2	Trim33-sgRNA-2	CACCGCGGCCACCCCCCCGTCGTCGT	TAAAACGACGACGGGGGGGTGGCCGC
Trim-library-2	Trim33-sgRNA-3	CACCGCGTCGATGCCGCGCCTCCGGT	TAAAACCGGAGGCGCGGCATCGACGC
Trim-library-2	Trim34a-sgRNA-1	CACCGGGACAAGAAGGCCATTTGCGT	TAAAACGCAAATGGCCTTCTTGTCCC
Trim-library-2	Trim34a-sgRNA-2	CACCGTTCTTCTTCGTGATGAGACGT	TAAAACGTCTCATCACGAAGAAGAAC
Trim-library-2	Trim34a-sgRNA-3	CACCGGCATGCTACAAAAGTTTAGGT	TAAAACCTAAACTTTTGTAGCATGCC
Trim-library-2	Trim34b-sgRNA-1	CACCGATGCAGTGATCAACCCCAGGT	TAAAACCTGGGGTTGATCACTGCATC
Trim-library-2	Trim34b-sgRNA-2	CACCGTTCTTGTTCGAGATGAGACGT	TAAAACGTCTCATCTCGAACAAGAAC
Trim-library-2	Trim34b-sgRNA-3	CACCGGCATGCTACAAAAGTTCAGGT	TAAAACCTGAACTTTTGTAGCATGCC

Trim-library-2	Trim36-sgRNA-1	CACCGAGAGAATAACGGGCTCGTGGT	TAAAACCACGAGCCCGTTATTCTCTC
Trim-library-2	Trim36-sgRNA-2	CACCGCTAGAGAATAACGGGCTCGGT	TAAAACCGAGCCCGTTATTCTCTAGC
Trim-library-2	Trim36-sgRNA-3	CACCGGGCTGCCCTAGCGGCCTGCGT	TAAAACGCAGGCCGCTAGGGCAGCCC
Trim-library-2	Trim39-sgRNA-1	CACCGCCATCGGCACGACGGTGTGGT	TAAAACCACACCGTCGTGCCGATGGC
Trim-library-2	Trim39-sgRNA-2	CACCGTACTGCCTAGCTGTCGGTTGT	TAAAACAACCGACAGCTAGGCAGTAC
Trim-library-2	Trim39-sgRNA-3	CACCGGGCCGGAGACTCCGGTATCGT	TAAAACGATACCGGAGTCTCCGGCCC
Trim-library-2	Trim3-sgRNA-1	CACCGCTGCAAGGCGCACACCATCGT	TAAAACGATGGTGTGCGCCTTGCAGC
Trim-library-2	Trim3-sgRNA-2	CACCGCGGAGATCGGTGCTCAACCGT	TAAAACGGTTGAGCACCGATCTCCGC
Trim-library-2	Trim3-sgRNA-3	CACCGCAGGCTCTGGTCAGCGACTGT	TAAAACAGTCGCTGACCAGAGCCTGC
Trim-library-2	Trim41-sgRNA-1	CACCGGTGCATCTGACGGATCACCGT	TAAAACGGTGATCCGTCAGATGCACC
Trim-library-2	Trim41-sgRNA-2	CACCGATAATCTTCTTCCCGCATAGT	TAAAACTATGCGGGAAGAAGATTATC
Trim-library-2	Trim41-sgRNA-3	CACCGGCACCCAACTCCTGGGCGAGT	TAAAACTCGCCCAGGAGTTGGGTGCC
Trim-library-2	Trim44-sgRNA-1	CACCGGCCTGGGCGCCATGGACGTGT	TAAAACACGTCCATGGCGCCCAGGCC
Trim-library-2	Trim44-sgRNA-2	CACCGCTGGCGATGCGCGTCGGCGGT	TAAAACCGCCGACGCGCATCGCCAGC
Trim-library-2	Trim44-sgRNA-3	CACCGACGACGCGCTCCCCGAAGAGT	TAAAACTCTTCGGGGAGCGCGTCGTC
Trim-library-2	Trim45-sgRNA-1	CACCGCTGCAGGCTACATAACTCGGT	TAAAACCGAGTTATGTAGCCTGCAGC
Trim-library-2	Trim45-sgRNA-2	CACCGCTGTGACCGGCCGGTGTGTGT	TAAAACACACACCGGCCGGTCACAGC
Trim-library-2	Trim45-sgRNA-3	CACCGACAGTCCCGACACACCGGCGT	TAAAACGCCGGTGTGTCGGGACTGTC
Trim-library-2	Trim56-sgRNA-1	CACCGAACGCACTGCTTCCGCTAGGT	TAAAACCTAGCGGAAGCAGTGCGTTC
Trim-library-2	Trim56-sgRNA-2	CACCGGAGGCCGAGATCCTTTCGCGT	TAAAACGCGAAAGGATCTCGGCCTCC
Trim-library-2	Trim56-sgRNA-3	CACCGCTCTGCAAGGATTGCCGCCGT	TAAAACGGCGGCAATCCTTGCAGAGC
Trim-	Trim68-	CACCGAAACTGGAGTTGAGG	TAAAACCGCGCCTCAACTC

library-2	sgRNA-1	CGCGGT	CAGTTTC
Trim-library-2	Trim68-sgRNA-2	CACCGGACTGCTTACAGGCCTCGCGT	TAAAACGCGAGGCCTGTAAGCAGTCC
Trim-library-2	Trim68-sgRNA-3	CACCGCTTCACGGGAGCTCGACAAGT	TAAAACTTGTCGAGCTCCCGTGAAGC
Trim-library-2	Trim71-sgRNA-1	CACCGCGCACTCACCTCATCGTCGGT	TAAAACCGACGATGAGGTGAGTGCGC
Trim-library-2	Trim71-sgRNA-2	CACCGGCGGCGTTGCCCTCGTCGCGT	TAAAACGCGACGAGGGCAACGCCGCC
Trim-library-2	Trim71-sgRNA-3	CACCGTCGAGGCACGGCCGGCAGAGT	TAAAACTCTGCCGGCCGTGCCTCGAC
Trim-library-2	Trim7-sgRNA-1	CACCGTGGATCTGGCCGCTGAGTTGT	TAAAACAACTCAGCGGCCAGATCCAC
Trim-library-2	Trim7-sgRNA-2	CACCGTCAAGAGTTTCGTCTTAAAGT	TAAAACTTTAAGACGAAACTCTTGAC
Trim-library-2	Trim7-sgRNA-3	CACCGTTTAAGACGAAACTCTTGAGT	TAAAACTCAAGAGTTTCGTCTTAAAC
Trim-library-2	Trim8-sgRNA-1	CACCGGGCTGCATCGGCGAGGCTTGT	TAAAACAAGCCTCGCCGATGCAGCCC
Trim-library-2	Trim8-sgRNA-2	CACCGAAGTTCAACGCCCTGCACGGT	TAAAACGTGCAGGGCGTTGAACTTC
Trim-library-2	Trim8-sgRNA-3	CACCGGTGGAGGCGGACGACGTGCGT	TAAAACGCACGTCGTCCGCCTCCACC
Trim-library-2	Triml2-sgRNA-1	CACCGTGTCTGTAGACGTTACTCCGT	TAAAACGGAGTAACGTCTACAGACAC
Trim-library-2	Triml2-sgRNA-2	CACCGAAGTTCAGGTCAACTTTGTGT	TAAAACACAAAGTTGACCTGAACTTC
Trim-library-2	Triml2-sgRNA-3	CACCGACTGAAGGCGAGTGAAGTCGT	TAAAACGACTTCACTCGCCTTCAGTC
