# Supplementary material for: TRIM37 interacts with TRIM28 to maintain primordial germ cell identity during migration
Source: Cell Res. 2026 Jul 30;36(10):785–803. doi: 10.1038/s41422-026-01272-2 (PMC13642344; doi:10.1038/s41422-026-01272-2)
Supplement: Supplementary file 10 [file 41422_2026_1272_MOESM19_ESM.pdf]

Table S3. Detailed information on mutations identified through genetic screening of semi-cloned embryos.

Library	Embryo	sgRNAs	sgRNA sequence	Indel efficiency	Analysis method	PGC phenotype
Trim-library-1	T1-2	Trim26-sgRNA-3	CCGCCCCATATCGGGGAAC	100.0%	TIDE	less
Trim-library-1	T1-3	Trim26-sgRNA-1	AGTGATATCCGCCCCATAT	100.0%	TIDE	normal
Trim-library-1	T1-4	Trim17-sgRNA-1	CCAGGAGCACCGGATGCAC	100.0%	TIDE	normal
Trim-library-1	T1-5	Trim12c-sgRNA-2	AACATTTCCTTATCGCATT	40.4%	TIDE	normal
Trim-library-1	T1-6	Trim37-sgRNA-2	AAACTTCGTCGACGTCTCA	0.0%	TIDE	normal
Trim-library-1	T1-7	Trim11-sgRNA-1	AGTGCATCCGACGTTGCTG	100.0%	TIDE	normal
Trim-library-1	T1-8	Multi-peaks	-	-	-	normal
Trim-library-1	T1-9	Trim25-sgRNA-2	AGGCCTCACTTCGACAGTC	100.0%	TIDE	normal
Trim-library-1	T1-10	Trim12c-sgRNA-2	AACATTTCCTTATCGCATT	67.2%	TIDE	normal
Trim-library-1	T1-28	Trim37-sgRNA-3	ATGAGACGTCGACGAAGTT	100.0%	TIDE	lack
Trim-library-1	T1-29	Trim37-sgRNA-3	ATGAGACGTCGACGAAGTT	100.0%	TIDE	lack
Trim-library-1	T1-30	Trim27-sgRNA-2	ACCGCTCGCGCGAGCACCG	100.0%	TIDE	normal
Trim-library-1	T1-45	Trim35-sgRNA-1	CAGACGACGCCCTCGTGTC	100.0%	TIDE	normal
Trim-library-1	T1-46	Trim27-sgRNA-2	ACCGCTCGCGCGAGCACCG	100.0%	TIDE	normal
Trim-library-1	T1-47	Trim11-sgRNA-1	AGTGCATCCGACGTTGCTG	100.0%	TIDE	normal
Trim-library-1	T1-48	Trim23-sgRNA-3	TCTAGGAGACTCAGGTGTA	100.0%	TIDE	normal
Trim-library-1	T1-49	Trim25-sgRNA-2	AGGCCTCACTTCGACAGTC	100.0%	TIDE	normal
Trim-library-1	T1-50	Trim25-sgRNA-2	AGGCCTCACTTCGACAGTC	100.0%	TIDE	normal
Trim-library-1	T1-52	Trim23-sgRNA-3	TCTAGGAGACTCAGGTGTA	91.0%	TIDE	normal

Trim-library-1	T1-53	Trim24-sgRNA-3	CTTGGGTGGCGAATGGCGA	100.0%	TIDE	normal
Trim-library-1	T1-56	Trim35-sgRNA-1	CAGACGACGCCCTCGTGTC	100.0%	TIDE	normal
Trim-library-1	T1-57	Trim11-sgRNA-3	GCCGACAGCTCGCGACACT	100.0%	TIDE	normal
Trim-library-2	T2-1	Trim34a-sgRNA-2	TTCTTCTTCGTGATGAGAC	100.0%	TIDE	normal
Trim-library-2	T2-2	Trim41-sgRNA-1	GTGCATCTGACGGATCACC	100.0%	TIDE	normal
Trim-library-2	T2-4	Trim34b-sgRNA-3	GCATGCTACAAAAGTTCAG	22.3%	TIDE	normal
Trim-library-2	T2-5	Trim45-sgRNA-1	CTGCAGGCTACATAACTCG	100.0%	TIDE	normal
Trim-library-2	T2-6	Trim32-sgRNA-3	TTCAGCACCGTCAGGTTGT	100.0%	TIDE	normal
Trim-library-2	T2-26	Trim44-sgRNA-1	GCCTGGGCGCCATGGACGT	100.0%	TIDE	normal
Trim-library-2	T2-27	Trim41-sgRNA-1	GTGCATCTGACGGATCACC	100.0%	TIDE	normal
Trim-library-2	T2-28	Trim39-sgRNA-1	CCATCGGCACGACGGTGTG	100.0%	TIDE	normal
Trim-library-2	T2-29	Trim32-sgRNA-3	TTCAGCACCGTCAGGTTGT	100.0%	TIDE	normal
Trim-library-2	T2-30	Trim32-sgRNA-2	ACACTGGCGGCAGATGGTA	99.9%	TIDE	normal
Trim-library-2	T2-31	Trim32-sgRNA-1	GGAGAAGTTGACTCGTCTA	100.0%	TIDE	normal
Trim-library-2	T2-34	Triml2-sgRNA-3	ACTGAAGGCGAGTGAAGTC	98.0%	TIDE	normal
Trim-library-2	T2-61	Triml2-sgRNA-3	ACTGAAGGCGAGTGAAGTC	98.1%	TIDE	normal
Trim-library-2	T2-73	Trim32-sgRNA-1	GGAGAAGTTGACTCGTCTA	100.0%	TIDE	normal
Trim-library-2	T2-74	Blank	GGGTCTTCGAGAAGAC	-	-	normal
Trim-library-2	T2-75	Triml2-sgRNA-3	ACTGAAGGCGAGTGAAGTC	100.0%	TIDE	normal
Trim-library-2	T2-76	Trim2-sgRNA-2	ACCATCCTCACCACTAACG	100.0%	TIDE	normal
Trim-library-2	T2-77	Trim7-sgRNA-2	TCAAGAGTTTCGTCTTAAA	0.0%	TIDE	normal
Trim-	T2-78	Trim32-	TTCAGCACCGTCAGG	100.0%	TIDE	normal

library-2		sgRNA-3	TTGT			
Trim-library-2	T2-79	Trim44-sgRNA-1	GCCTGGGCGCCATGGACGT	100.0%	TIDE	less
Trim-library-2	T2-80	Trim36-sgRNA-1	AGAGAATAACGGGCTCGTG	100.0%	TIDE	normal
Trim-library-2	T2-81	Trim41-sgRNA-2	ATAATCTTCTTCCCGCATA	100.0%	TIDE	normal
Trim-library-2	T2-88	Trim7-sgRNA-2	TCAAGAGTTTCGTCTTAAA	0.0%	TIDE	normal
Trim-library-2	T2-94	Blank	GGGTCTTCGAGAAGAC	-	-	normal
Trim-library-2	T2-95	Trim36-sgRNA-3	GGCTGCCCTAGCGGCCTGC	100.0%	TIDE	normal
Trim-library-2	T2-96	Trim32-sgRNA-3	TTCAGCACCGTCAGGTTGT	100.0%	TIDE	normal
Trim-library-2	T2-97	Triml2-sgRNA-3	ACTGAAGGCGAGTGAAGTC	98.0%	TIDE	normal
Trim-library-2	T2-98	Trim33-sgRNA-1	GGCTGGGCCCGACGACGGG	99.8%	TIDE	normal
Trim-library-2	T2-99	Triml2-sgRNA-2	AAGTTCAGGTCAACTTTGT	100.0%	TIDE	normal
Trim-library-2	T2-100	Trim3-sgRNA-3	CAGGCTCTGGTCAGCGACT	100.0%	TIDE	normal
Trim-library-2	T2-101	Blank	GGGTCTTCGAGAAGAC	-	-	normal
Trim-library-2	T2-102	Trim7-sgRNA-1	TGGATCTGGCCGCTGAGTT	100.0%	TIDE	normal
Trim-library-2	T2-103	Trim32-sgRNA-2	ACACTGGCGGCAGATGGTA	100.0%	TIDE	normal
Trim-library-2	T2-104	Trim39-sgRNA-1	CCATCGGCACGACGGTGTG	100.0%	TIDE	normal
Indel efficiency = 100% - percentage of wild-type allele, analysing by TIDE (Tracking of Indels by Decomposition, https://tide.nki.nl/)						
