# Supplementary material for: TRIM37 interacts with TRIM28 to maintain primordial germ cell identity during migration
Source: Cell Res. 2026 Jul 30;36(10):785–803. doi: 10.1038/s41422-026-01272-2 (PMC13642344; doi:10.1038/s41422-026-01272-2)
Supplement: Supplementary file 11 [file 41422_2026_1272_MOESM1_ESM.pdf]

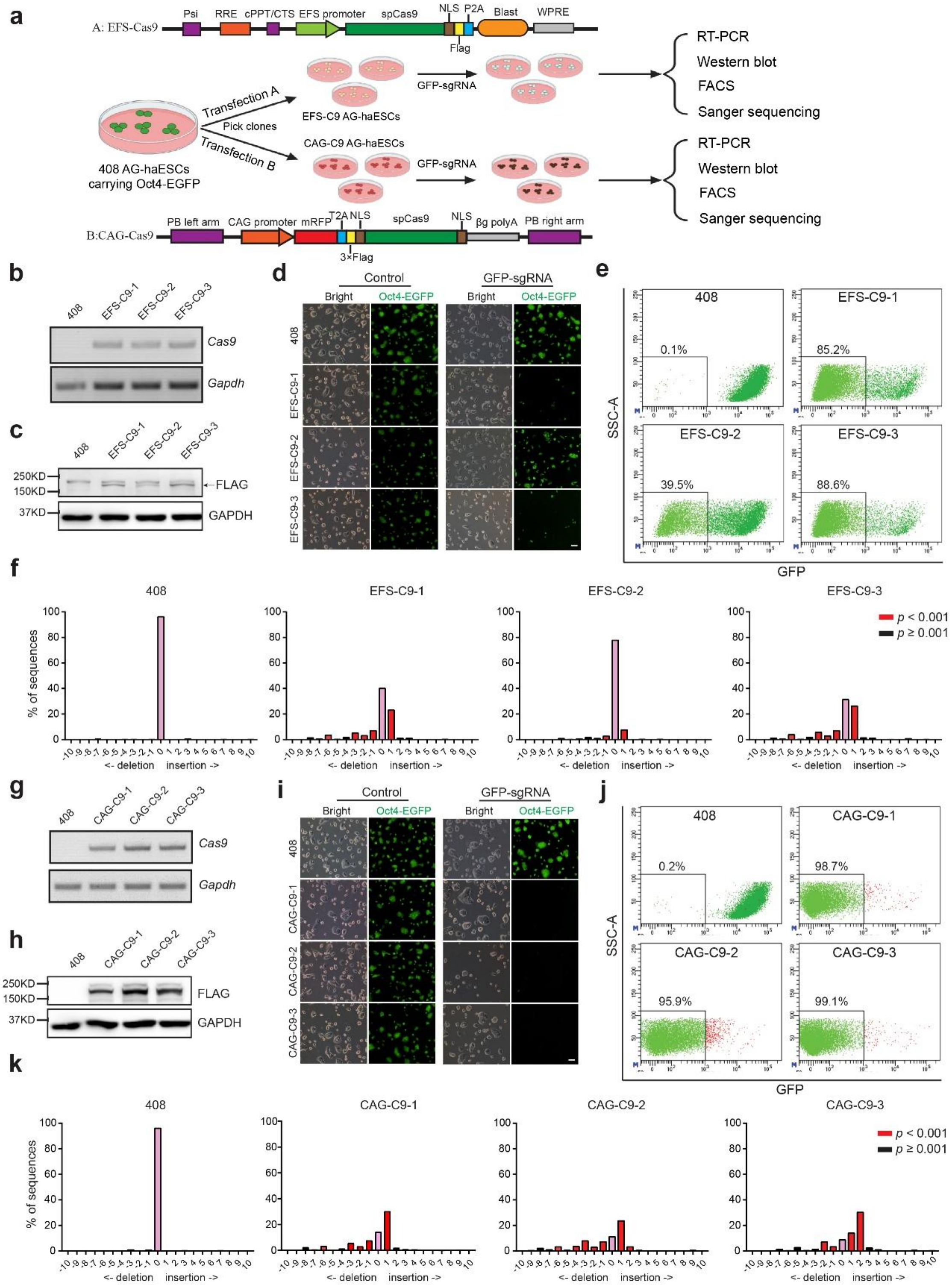

Fig. S1 Establishment of an enhanced Cas9 expression system in AG-haESCs. a Schematic illustration depicting the evaluation of editing efficiency in AG-haESCs using EFS-Cas9 and CAG-Cas9. EFS-Cas9 plasmid: Psi, retroviral Ψ packaging element; RRE, Rev response element; cPPT/CTS, central polypurine tract and central termination sequence of HIV-1; EFS, EFS promoter; NLS, nuclear location sequence; P2A, a 2A self-cleavage peptide from porcine teschovirus-1; Blast, blasticidin S deaminase; WPRE, Woodchuck hepatitis virus post-transcriptional regulatory element. CAG-Cas9 plasmid: CAG, CMV immediate enhancer/β-actin promoter; βg polyA, β-globin polyA

signal. **b** RT-PCR analysis of *Cas9* expression in different AG-haESC lines. 408 AG-haESCs carried Oct4-EGFP transgene. EFS-C9-1 to EFS-C9-3 cell lines were derived from 408 by infection with the Lenti-Cas9 virus. *Gapdh* was used as a control. **c** Western blot analysis of Cas9 expression in the indicated AG-haESC lines. The Cas9 protein is tagged with Flag, and the targeted band is indicated by the arrow. GAPDH was used as a loading control. **d** Representative images of EFS-C9-1 to EFS-C9-3 AG-haESCs infected by GFP-sgRNA lentivirus, which targets the EGFP gene. Scale bar, 100 μm. **e** Representative FACS results showing Oct4-EGFP signals in 408, EFS-C9-1, EFS-C9-2, and EFS-C9-3 AG-haESCs. **f** TIDE-mediated analysis of EGFP sequence in 408, EFS-C9-1, EFS-C9-2, and EFS-C9-3 AG-haESCs, showing indels in the cells expressing Cas9 and GFP-sgRNA. **g** RT-PCR analysis of *Cas9* expression in different AG-haESC lines. CAG-C9-1 to CAG-C9-3 were derived from 408 by transfection of the PB-Cas9 vector. *Gapdh* was used as a control. **h** Western blot analysis of Cas9 expression in different AG-haESC lines. The Cas9 protein is tagged with 3*x*Flag. GAPDH is used as a loading control. **i**, Representative images of CAG-C9-1 to CAG-C9-3 AG-haESCs infected by GFP-sgRNA lentivirus, which targets the EGFP gene. Scale bar, 100 μm. **j** Representative FACS results showing Oct4-EGFP signals in 408, CAG-C9-1, CAG-C9-2, and CAG-C9-3 AG-haESCs. **k** TIDE-mediated analysis of EGFP sequence in 408, CAG-C9-1, CAG-C9-2, and CAG-C9-3 AG-haESCs, showing indels efficiently happening in the cells expressing Cas9 and GFP-sgRNA.
