# Supplementary material for: TRIM37 interacts with TRIM28 to maintain primordial germ cell identity during migration
Source: Cell Res. 2026 Jul 30;36(10):785–803. doi: 10.1038/s41422-026-01272-2 (PMC13642344; doi:10.1038/s41422-026-01272-2)
Supplement: Supplementary file 12 [file 41422_2026_1272_MOESM20_ESM.pdf]

Table S4. Candidates of TRIM37 interacted proteins through IP-MS.

Bands	Protein	MW [kDa]	Protein name	WT-1	WT-2	WT-3	Trim37 3xFlag-1	Trim37 3xFlag-2	Trim37 3xFlag-3
Band A	Q60865	78.1	Caprin1	-	-	-	5058883	1879087.75	5043165.313
Band A	Q5EBP9	88.8	Trim28	-	-	0	1637251.875	4791107.938	4313127.5
Band A	Q80X98	140.5	Dhx38	-	0	-	3821999.125	1646641.969	4940984.063
Band A	Q8K010	137.5	Oplah	-	-	-	381635.4375	853439.0625	7506174.422
Band A	Q99KG3	103.4	Rbm10	-	-	-	3971484.313	716392.9375	3300763.531
Band A	B1ASP2	133.3	Jak1	-	-	-	2281675.313	1169984.031	3121236.531
Band A	Q6P5E4	176.3	Uggt1	-	0	-	3604061.063	472206.8438	2229850.281
Band A	Q9EPU0	123.9	Upf1	-	0	-	1476347.656	1779376.5	1995143.406
Band A	G5E866	145.7	Sf3b1	-	-	-	2605273.688	1439082.688	600468.25
Band B	Q60972	47.6	Rbbp4	-	-	-	709911.1875	0	3286672
Band A	Q3TFU3	65.4	Maged2	-	-	-	462272.25	2745809.125	478490.1563
Band A	Q8CI08	62.3	Slain2	-	-	-	1974145.156	360966.5625	1118774.75
Band A	A0A5F8MPE3	133.7	Prdm11	-	-	-	1648282.656	0	1206502.063
Band A	Q6ZQ61	95.2	Matr3	-	-	-	2176224.313	412027.7188	0
Band A	A3KGU9	287.4	Sptan1	-	-	0	708039.9063	212456.4688	1536182.875
Band C	Q9CX86	30.5	Hnrnpa0	0	-	-	891263	0	1355342.875
Band A	Q6ZQ58	121.1	Larp1	-	-	-	1454177.625	700677.375	0
Band A	Q3TI61	100.2	Psmd2	-	-	-	1345466.625	320186.375	436475.8438
Band A	A0A1B0GS91	253.1	Svil	-	-	0	0	1879062	0
Band A	Q5SUF2	51.4	Luc7l3	-	-	-	905969.875	390155.8438	438146.625
Band A	P58252	95.3	Eef2	-	-	-	353554.625	0	1213023.063
Band A	Q99JF8	59.7	Psip1	-	-	-	617857.9375	455568.8125	367062.4063
Band A	Q80VC9	135.1	Camsap3	-	-	-	461055.4375	0	916920.6875
Band A	Q6PCX9	107.6	Trim37	-	-	-	737724	626493.8125	0
Band A	Q8VDM6	95.9	Hnrnpul1	-	-	-	402770.4688	174868.75	752346.875
Band A	Q3TZP3	75.0	Mta2	-	-	-	204181.2031	0	1054318.75
Band C	Q5FW97	47.1	EG433182	0	-	-	0	0	1182789.625
Band A	A2A9M4	241.3	Dock7	-	-	-	731083.625	413910.0938	0
Band A	M1VMF6	82.3	Mta1	-	-	-	627934.625	0	494749.2188
Band A	Q059U9	76.2	Akap8	-	-	-	376492.5938	424567.7813	0
Band C	P62858	7.8	Rps28	-	-	-	0	0	656828.625
Band A	H3BJW3	63.4	Cpsf6	-	-	-	0	560941.8125	0
Band A	Q0VBL3	105.7	Rbm15	-	-	-	0	0	486957.0625

Band A	Q01320	172.7	Top2a	-	-	-	132220.0625	189749.25	0
Band A	Q91VX2	117.9	Ubap2	-	-	-	0	0	291508.3438
Band A	P38647	73.4	Hspa9	-	-	-	0	263904.5313	0
Band A	F8VQD1	194.6	Pbrm1	-	-	-	0	212417.7031	0
Band B	Q3U9N8	55.7	Impdh2	-	-	0	0	0	202967.7344
Band A	O08856	67.1	Ell	-	-	-	0	0	0

Note: The abundance values in columns F to K were calculated using Proteome Discoverer software (version 1.4; Thermo Fisher Scientific, USA) based on the original spectrum. A value of '0' indicates that the protein was identified, but its peak area was too small for quantification, so it is reported as 0.
