# Supplementary material for: TRIM37 interacts with TRIM28 to maintain primordial germ cell identity during migration
Source: Cell Res. 2026 Jul 30;36(10):785–803. doi: 10.1038/s41422-026-01272-2 (PMC13642344; doi:10.1038/s41422-026-01272-2)
Supplement: Supplementary file 13 [file 41422_2026_1272_MOESM21_ESM.pdf]

Table S5. List of primer information related to experimental procedures.

SgRNA sequences for the construction of cell and mouse lines	
sgRNA Name	Sequence (5'-3')
GFP-sgRNA	GTAGGTCAGGGTGGTCACGAGGG
Dnd1-sgRNA1	TGGTGCAGGTCAACGGGCAAAGG
Dnd1-sgRNA2	ACGGGCAAAGGAAGTATGGAGGG
Dnd1-sgRNA3	GAAGGTCAGTCACGCCTCTGTGG
Trim37-KO-sgRNA	GGGAACTGGTGAACTGTCGTTGG
Trim37-KO2-sgRNA	CATTTGTATGGAGAAATTGCGGG
Trim37-C18R-sgRNA	TTTCCGATGTTTCATTTGTATGG
Trim37-N-3*x*Flag-sgRNA	CTCTGCTCATCCATTGCCTCCGG
Trim37-flox-sgRNA1	GGTTACTTAATACCTGTGGGGGG
Trim37-flox-sgRNA2	CAGTAGGTACTATATCTGTGAGG
Trim37-NLS/NES-sgRNA	TACCTACTGTTGAGAAGAGAAGG
Trim37-delRING-sgRNA1	CATCCCAGCTATGACACGACAGG
Trim37-delRING-sgRNA2	TAAAACATGGTACTCGAGCAGGG
Trim37-delMATH-sgRNA1	ATGCTGTGCTTCGTAAAGATTGG
Trim37-delMATH-sgRNA2	TGAAGTGGTTCTTATCGAATGGG
Trim37-G322V-sgRNA	GATGGAAATGGAGTTGTACGTGG
FINmajor-sgRNA	TTCCTAGAAGATAAACACGAAGG
Trim37-sgRNA-stop	CATCAGTGTGCACTTTGGGGAGG
Trim28-flox-sgRNA1	GTAGGGTTAAGTAGGCCTGCTGG
Trim28-flox-sgRNA2	CCAGACACTGAGACCGCATAAGG
Trim28-K774R/K779R-sgRNA	ACAGTTCAACAAGCTGACTGAGG
Blimp1-T2A-Cre-sgRNA	TGAAAATCTTAAGGATCCATCGG
Genotyping of edited cells and mouse lines	
Primer Name	Sequence (5'-3')
Oct4-GFP-checkF	CATGGTGAGCCGTCTTTCCA
Oct4-GFP-checkR	TCGATGTTGTGGCGGATCTT
Dnd1-sg1/2-checkF	TCTAACGAGGTGGACCAGGG
Dnd1-sg1/2-checkR	AAGGGAGGCCAGACAAATAGC
Dnd1-sg3-checkF	GTGCTTGCGGCTCCTAGTG
Dnd1-sg3-checkR	AGGGCAAGGTTCCTCACAACTAA
Trim37-KO-check-F	AGCTTCAGTTCTGTAACCTTAGTG
Trim37-KO-check-R	GCATGTGGCCCCAGGATAAA
Trim37-KO2/C18R-check-F	TCAGTGTAATCTGGGACCCT
Trim37-KO2/C18R-check-R	AAAAAGGGGAGGGCATGGTT
Trim37-3*x*flag-check-F	TGGCGAAATGCGGGGTAG
Trim37-3*x*flag-check-R	ATAGAAAAGGAGCCCAGCCG
Trim37-flox1-check-F	TTTTCCGTTTGCGCCTTCC
Trim37-flox1-check-R	GTCATTTACTGAGGAACGGACT

Trim37-flox2-check-F	TGGATAGGCATGCTATTCTTGTATG
Trim37-flox2-check-R	AGCGAGGTTAACCGGAAGGA
Trim37-G322V-checkF	CTGTTTGTTGGCTTCATTGT
Trim37-G322V-checkR	AAGAGTTTCGACACAGCAAT
FINmajor-check-F	TCTTTCACCTTACTGACCCAAA
FINmajor-check-R	TGGCAACAGAATGCAAGCAC
Trim37-NLS/NES-check F	CAGTCTGGGAGCAGACACAG
Trim37-NLS/NES-check R	CACAGCACATGACACAGCAC
Trim37-wtRING-check-F	TTGTACCTGAATTCTGGGGTTCTC
Trim37-wtRING-check-R	ACATTAAAAAGGGGAGGGCATGG
Trim37-delRING-check-F	TCAGTCAGCTCGTCCTATGC
Trim37-delRING-check-R	TCTGTGCAGCTTCCAACTGA
Trim37-wtMATH-check-F	TGTGAGTTATCAGCGAGAGGC
Trim37-wtMATH-check-R	CACCAGCCTGGGTATGAGTC
Trim37-delMATH-check-F	TCAGTCAGCTCGTCCTATGC
Trim37-delMATH-check-R	TCTGTGCAGCTTCCAACTGA
Trim28-K774R/K779R-checkF	AGCTGGCTACCGACTCTACA
Trim28-K774R/K779R-checkR	TGGTTCTACCAGCACAGCAG
Trim28-flox-checkF	GCGCTAGTGAGTACTGGGTG
Trim28-flox-checkR	ATGCACTCAAATCTGTGCCG
Blimp1-T2A-Cre-checkF	CAGCAACCCACTACCTCTGG
Blimp1-T2A-Cre-checkR	TGAAAATCTTAAGGATCCATCGG
LentisgRNA-deep-F	CTTGAAAGTATTTCGATTTCTTGG
LentisgRNA-deep-R	ACTCGGTGCCACTTTTTCAA
Primers for RT-PCR and qPCR	
Primer Name	Sequence (5'-3')
Gapdh-qPCR-F	CACTCTTCCACCTTCGATGC
Gapdh-qPCR-R	CTCTTGCTCAGTGTCCTTGC
Cas9-qPCR-F	CTGAGCAAGGACACCTACGA
Cas9-qPCR-R	CTCGGTGTTCACTCTCAGGA
Trim37-qPCR-F	TCCAAGCTCTGTTGTTTCAGC
Trim37-qPCR-R	TTCCGCCCAACGACAGTTC
