# Supplementary material for: TRIM37 interacts with TRIM28 to maintain primordial germ cell identity during migration
Source: Cell Res. 2026 Jul 30;36(10):785–803. doi: 10.1038/s41422-026-01272-2 (PMC13642344; doi:10.1038/s41422-026-01272-2)
Supplement: Supplementary file 14 [file 41422_2026_1272_MOESM2_ESM.pdf]

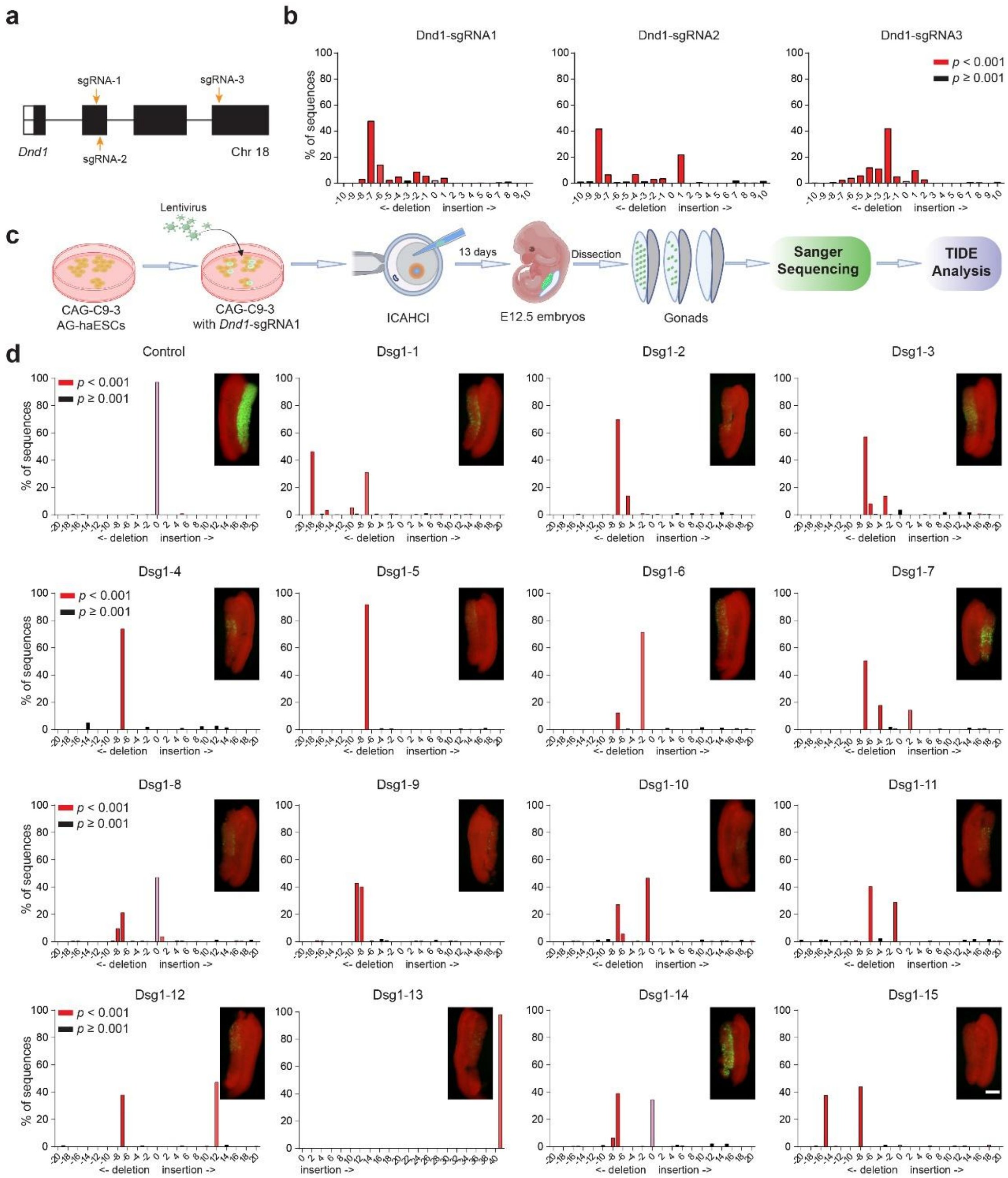

Fig. S2 CAG-C9-3 AG-haESC supports efficient generation of biallelic mutant semi-cloned embryos. a Schematic of three sgRNA-targeting sites in *Dnd1*. **b** TIDE-mediated analysis of *Dnd1* sequence in CAG-C9-3 AG-haESCs that expressed different sgRNAs, showing almost complete indel mutations. **c** Schematic of generating *Dnd1* biallelic mutant semi-cloned embryos subjected to phenotype and genotype analysis. ICAHCI, intracytoplasmic AG-haESC injection. **d** TIDE-mediated sequence analysis of semi-cloned embryos carrying constitutively expressed Cas9 and sgRNA targeting *Dnd1*. The images representing the phenotype of E12.5 gonads are shown. The red signals indicate Cas9 expression, and the green signals represent Oct4-EGFP-positive PGCs. Scale bar, 200 μm.
