# Supplementary material for: TRIM37 interacts with TRIM28 to maintain primordial germ cell identity during migration
Source: Cell Res. 2026 Jul 30;36(10):785–803. doi: 10.1038/s41422-026-01272-2 (PMC13642344; doi:10.1038/s41422-026-01272-2)
Supplement: Supplementary file 15 [file 41422_2026_1272_MOESM3_ESM.pdf]

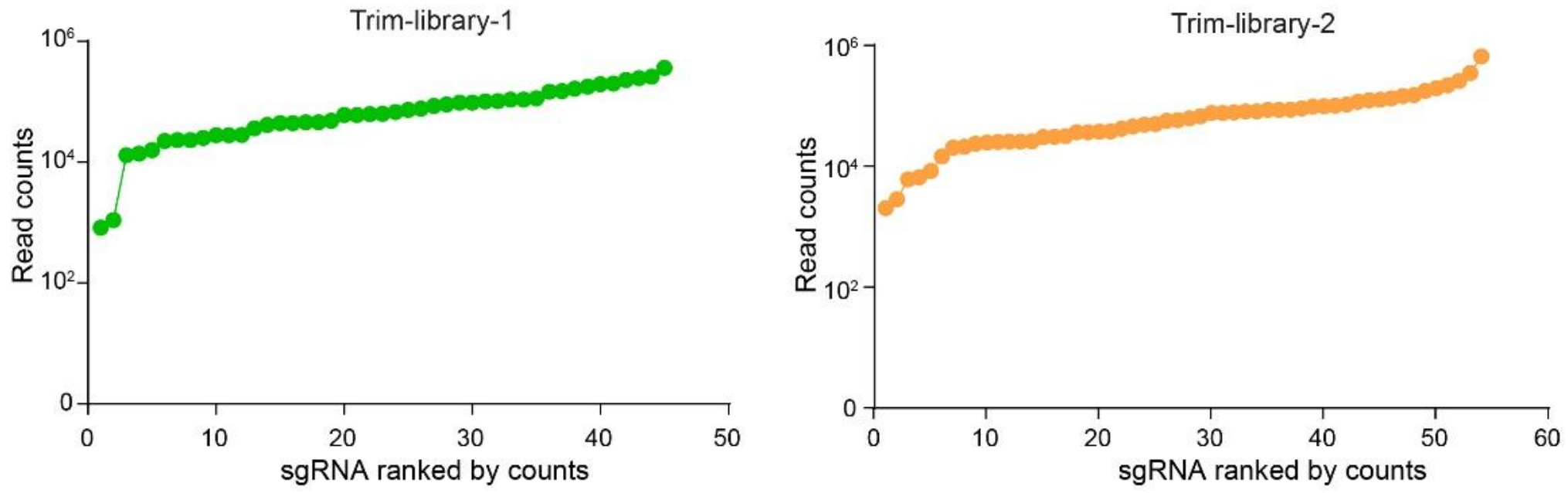

b

Summary of *in vivo* development of semi-cloned embryos derived from CAG-C9-3 with sgRNA library

Libraries	No. of genes	No. of sgRNAs	No. of two-cell embryos transferred	No. of normal embryos (% of transferred embryos)
Trim-library-1	15	45	1102	127 (11.5)
Trim-library-2	18	54	784	111 (14.2)

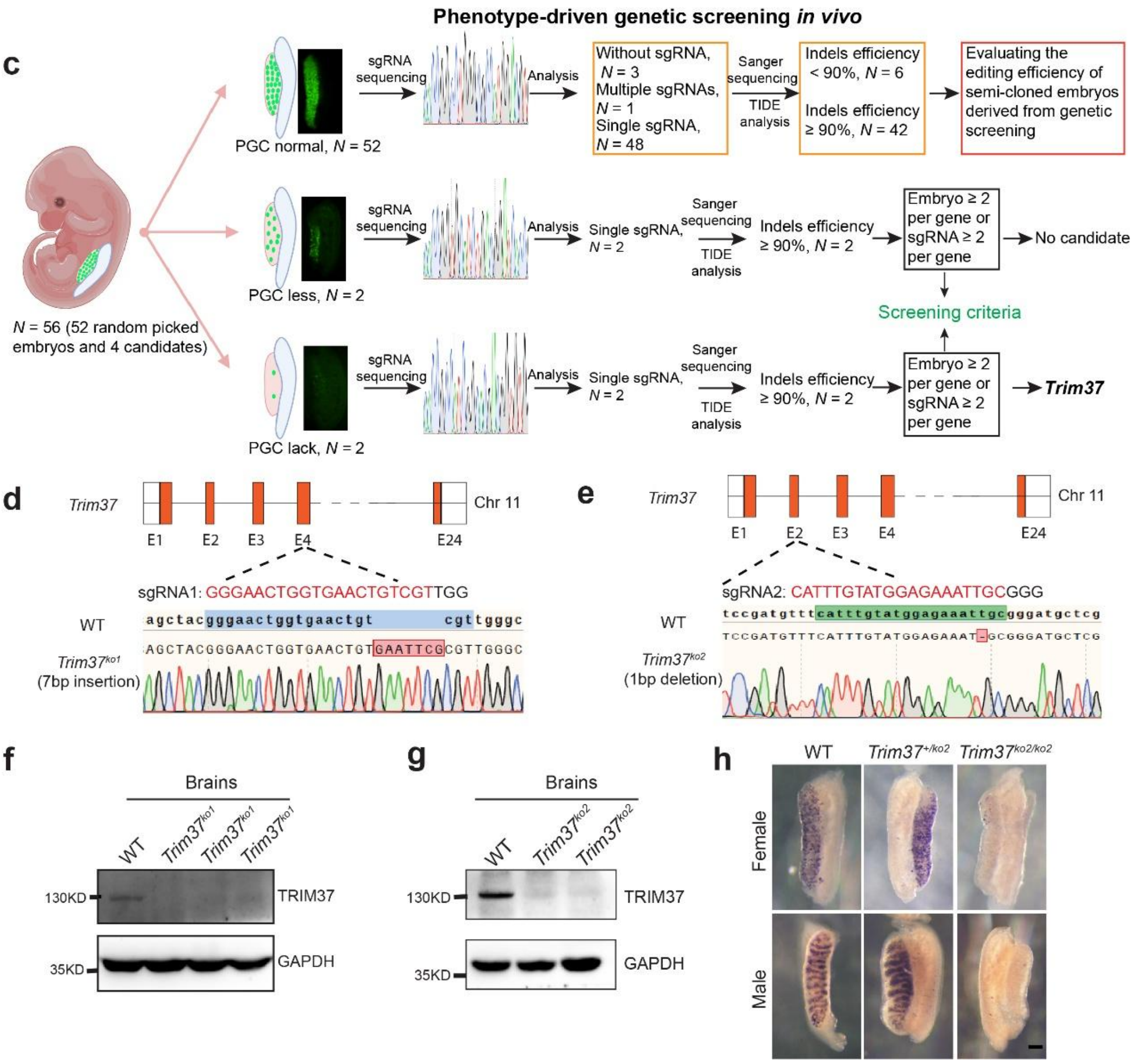

Fig. S3 Semi-cloning technology–mediated genetic screening identifies *Trim37* as an important regulator of mouse PGC development. a Deep sequencing analysis showed the sgRNA distribution of Trim-library-1 and Trim-library-2 in CAG-C9-3 AG-haESCs. **b** Summary of the generation of E12.5 semi-cloned embryos from CAG-C9-3 AG-haESCs carrying Trim-library-1 or Trim-library-2. **c**, Schematic of analyzing the screening results from phenotype-driven genetic

screening *in vivo*. The detailed information for these 56 checked embryos is provided in Supplementary Table 3. **d**, **e** The target sequences and genotyping of sgRNA1 (**d**) and sgRNA2 (**e**) for editing *Trim37* exon 4 and exon 2, resulting in a 7 bp insertion ($Trim37^{ko1}$) and a 1 bp deletion ($Trim37^{ko2}$), respectively. **f**, **g** Western blots showing the TRIM37 expression levels in the brain of $Trim37^{ko1}$ (**f**) and $Trim37^{ko2}$ (**g**) compared to wild-type mice. GAPDH is shown as a control for the amount of protein loading in each lane. Because the detection sensitivity of the anti-TRIM37 antibody is very low, brains with high expression of *Trim37* were chosen for detection. **h** Analysis of PGCs in E12.5 wild-type, $Trim37^{+/ko2}$, and $Trim37^{ko2/ko2}$ gonads by in situ hybridization of *Stella* probe, showing no PGCs in $Trim37^{ko2/ko2}$ gonads. Five embryos were used for each group. Scale bar, 100 μm.
