# Supplementary material for: TRIM37 interacts with TRIM28 to maintain primordial germ cell identity during migration
Source: Cell Res. 2026 Jul 30;36(10):785–803. doi: 10.1038/s41422-026-01272-2 (PMC13642344; doi:10.1038/s41422-026-01272-2)
Supplement: Supplementary file 16 [file 41422_2026_1272_MOESM4_ESM.pdf]

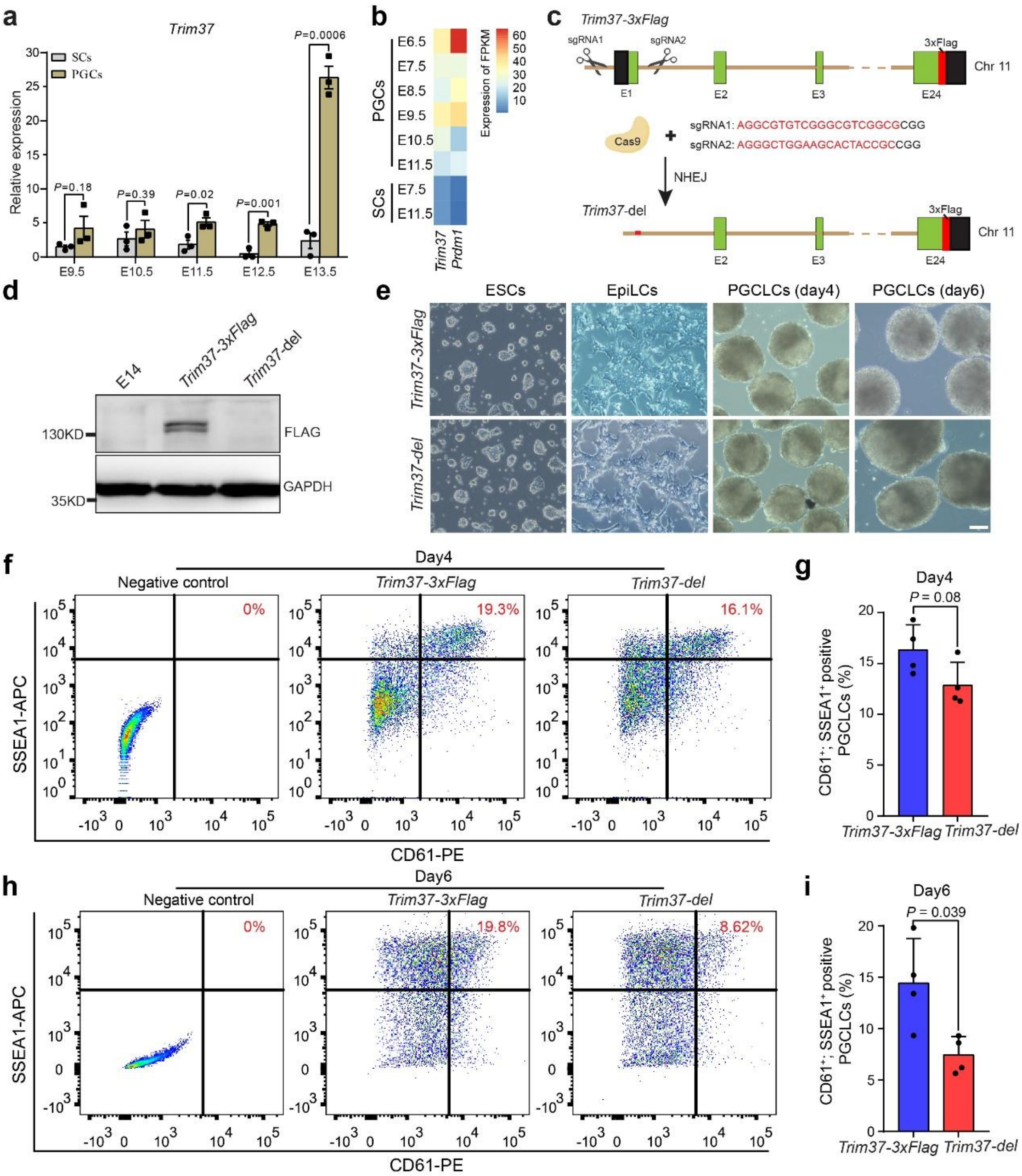

Fig. S4 *Trim37* knockout impairs the differentiation of PGC-like cells *in vitro*. a Transcriptional analysis of *Trim37* in somatic cells (SCs) and PGCs at different developmental stages (E9.5, E10.5, E11.5, E12.5, and E13.5). The expression values were normalized to those of *Gapdh*. $n = 3$ samples for each group. The *P*-value was calculated by the unpaired two-sided Student's t-test. **b** The heatmap shows the FPKM values of *Blimp1* and *Trim37* in PGCs from E6.5 to E11.5 and SCs at E7.5 and E11.5, based on RNA-seq data from a previous work [12]. **c** Schematic diagram of the construction of *Trim37* knockout embryonic stem cells (ESCs) based on E14-derived *Trim37-3xFlag* ESCs. The red bases represent the sgRNA sequence. The two sgRNAs were designed to delete exon 1 of *Trim37*. **d** Western blots showing the TRIM37 expression identified by anti-Flag antibody in the E14, *Trim37-3xFlag*, and *Trim37-del* ESCs. GAPDH is shown as a control for the amount of protein loading in each lane. **e** Representative images of ESCs, epiblast-like cells (EpiLCs), and PGC-like cells (PGCLCs at day 4 and day 6) from *Trim37-3xFlag* and *Trim37-del* ESCs. Scale bar, 100 µm. **f** Cells of the indicated genotypes were assessed by flow cytometry for

surface expression of SSEA1 and CD61 on day 4 of PGCLC differentiation. *Trim37-3 ×Flag* PGCLCs without antibody staining were used as the negative control. **g** Barplots showing the ratios of CD61 and SSEA1 double positive PGCLCs (*Trim37-3xFlag* and *Trim37-del*) at day 4. n = 4 independent experiments. **h** Cells of the indicated genotypes were assessed by flow cytometry for surface expression of SSEA1 and CD61 at day 6 of PGCLC differentiation. *Trim37-3 ×Flag* PGCLCs without antibody staining were used as the negative control. **i** Barplots showing the ratios of CD61 and SSEA1 double positive PGCLCs (*Trim37-3xFlag* and *Trim37-del*) at day 6. n = 4 independent experiments. Data are shown as the average mean ± s.e.m. The *P*-value was calculated by the paired two-sided Student's t-test in panels **g** and **i**.
