# Supplementary material for: TRIM37 interacts with TRIM28 to maintain primordial germ cell identity during migration
Source: Cell Res. 2026 Jul 30;36(10):785–803. doi: 10.1038/s41422-026-01272-2 (PMC13642344; doi:10.1038/s41422-026-01272-2)
Supplement: Supplementary file 17 [file 41422_2026_1272_MOESM5_ESM.pdf]

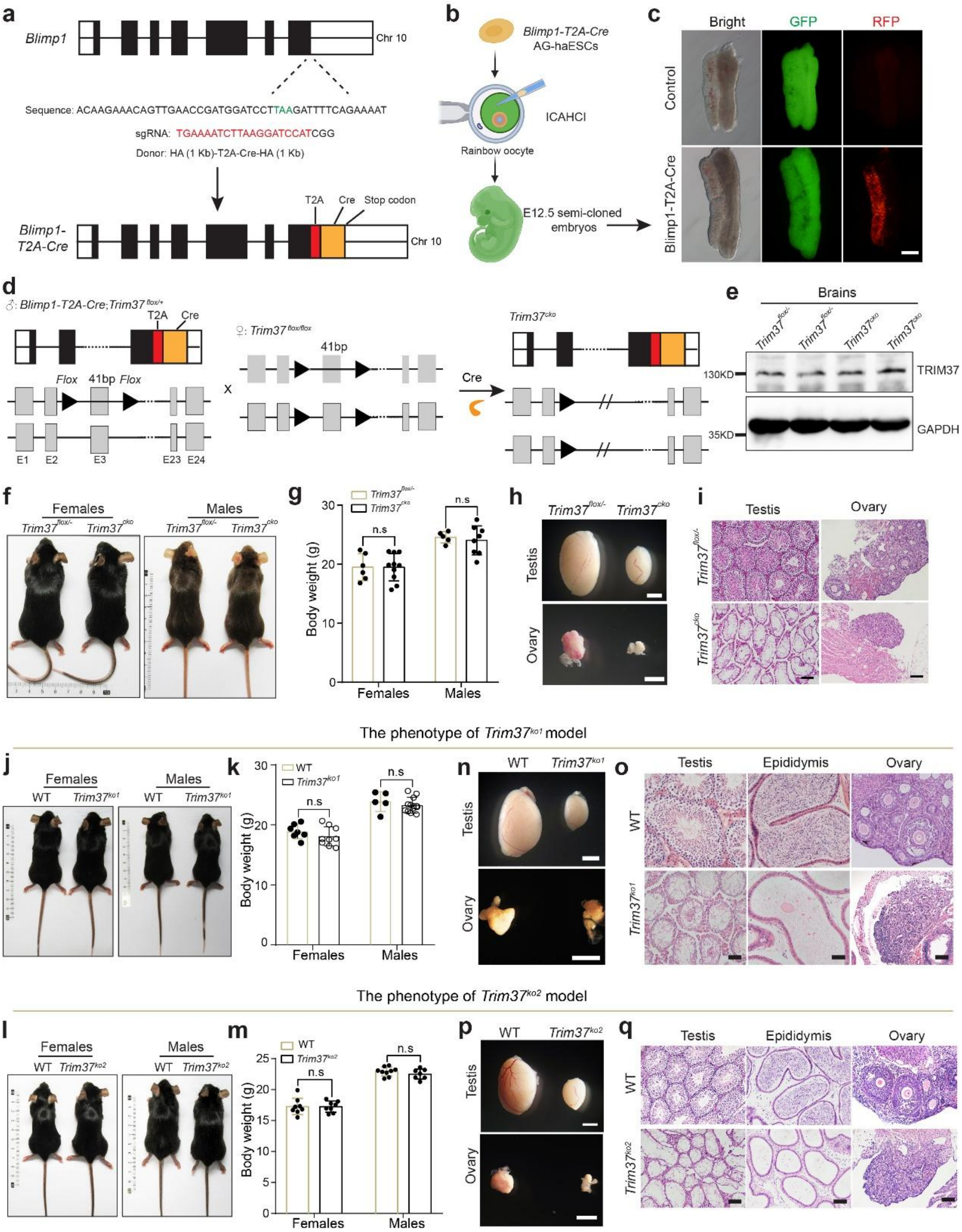

Fig. S5 *Trim37* mutants recapitulate infertility but not growth failure observed in MUL patients. a Schematic diagram of the construction of the *Blimp1-T2A-Cre* mouse model by CRISPR-Cas9-mediated homologous recombination. The red bases represent the sgRNA sequence. **b** Schematic of the injection of *Blimp1-T2A-Cre* AG-haESCs into Rainbow oocytes to generate E12.5 embryos. **c** Representative images of gonads from the injection of control or *Blimp1-T2A-Cre* AG-haESCs into the oocyte collected from Rainbow females. *Cre* under the promoter of *Blimp1* is specifically expressed in PGCs. Most GFP signals will transform to RFP in Rainbow cells that express the Cre enzyme [29]. The RFP signals only appear in the gonads, where PGCs colonize, indicating that the *Blimp1-T2A-Cre* model is very specific for labeling PGCs. Scale bar, 200 μm. **d**

Strategy of generating *Trim37*^{cko} model using *Blimp1-T2A-Cre* and *Trim37*$^{flox/flox}$ parents. **e** Western blots showing TRIM37 expression levels in the brain of *Trim37*$^{flox/-}$ and *Trim37*^{cko} mice, indicating that TRIM37 expression is normal in the brain of *Trim37*^{cko} mice. GAPDH is shown as a control for the amount of protein loading in each lane. **f**, **j**, **l** Images of *Trim37*^{cko} (**f**), *Trim37*^{ko1} (**j**), and *Trim37*^{ko2} (**l**) mice at eight weeks, including females and males. **g**, **k**, **m** Average body weight of *Trim37*^{cko} (**g**), *Trim37*^{ko1} (**k**), and *Trim37*^{ko2} (**m**) females and males at eight weeks. Data are presented as mean ± s.e.m, with each circle representing an individual mouse. n.s, no significant difference by the student's unpaired two-sided t-test in panels. **h**, **n**, **p** Morphology analysis showed testis and ovary from *Trim37*^{cko} (**h**), *Trim37*^{ko1} (**n**), and *Trim37*^{ko2} (**p**) mice were severely atrophied. Scale bars, 2 mm. **i**, **o**, **q** Histological analysis of the testis and ovary of the *Trim37*^{cko} (**i**), *Trim37*^{ko1} (**o**), and *Trim37*^{ko2} (**q**) mice, showing no germ cells in the mutant organs. Three adult mice (8 weeks of age) were used for each group. Scale bar in **i**, 100 μm; Scale bars in **o** and **q**, 50 μm.
