# Supplementary material for: TRIM37 interacts with TRIM28 to maintain primordial germ cell identity during migration
Source: Cell Res. 2026 Jul 30;36(10):785–803. doi: 10.1038/s41422-026-01272-2 (PMC13642344; doi:10.1038/s41422-026-01272-2)
Supplement: Supplementary file 18 [file 41422_2026_1272_MOESM6_ESM.pdf]

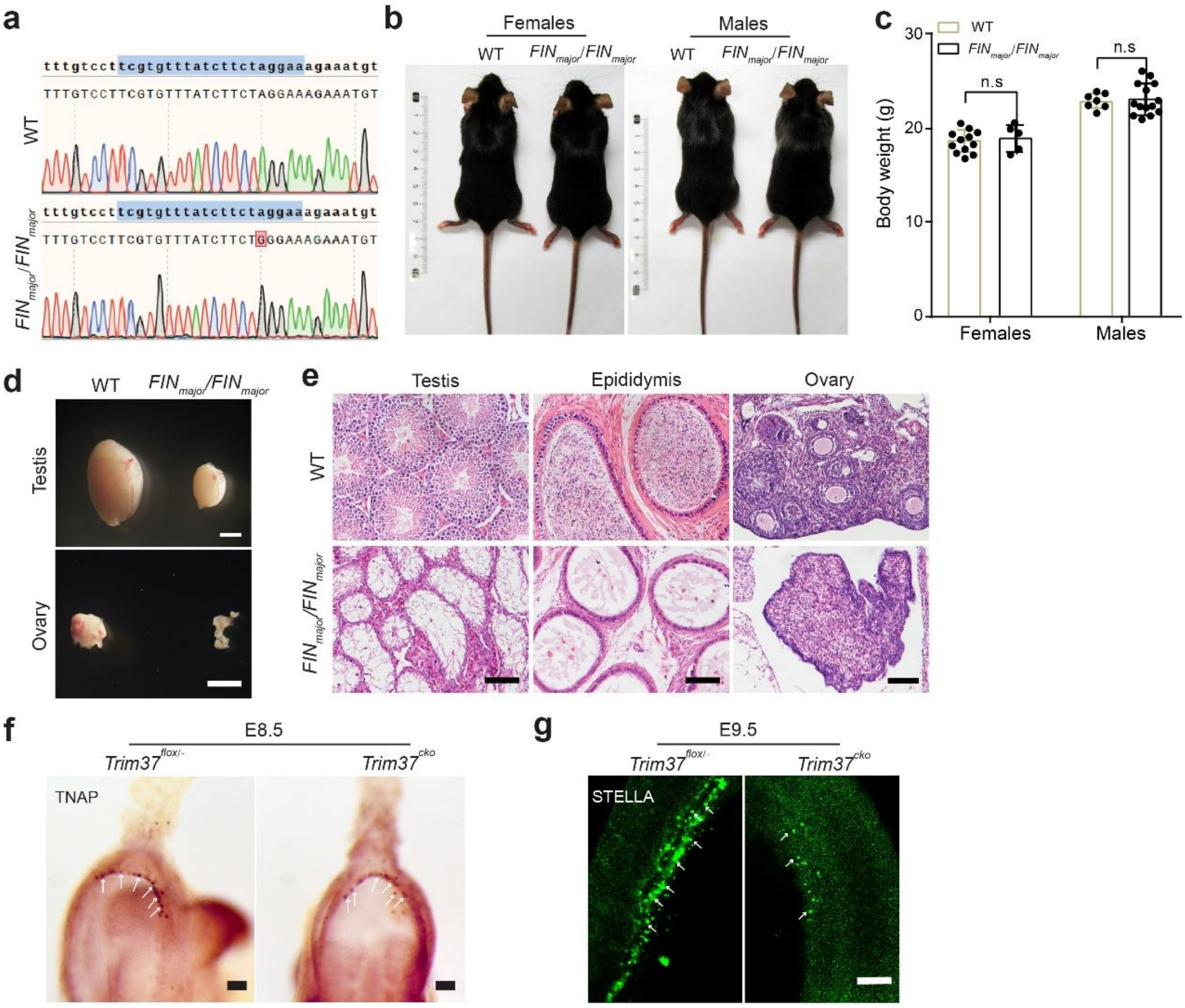

Fig. S6 Patient-derived mutation in *TRIM37* mimics infertility in mice. **a** Sanger sequencing of the patient-derived mutation, FIN_{major} (A-to-G substitution at the splice site), in the FIN_{major}/FIN_{major} mouse model. **b** Images of wild-type and FIN_{major}/FIN_{major} mice at eight weeks, including females and males. **c** Average body weight of FIN_{major}/FIN_{major} compared to wild-type mice, including males and females at eight weeks. n.s, no significant difference by the student's unpaired two-sided t-test. **d** Morphology analysis showed testis and ovary from FIN_{major}/FIN_{major} mice were severely atrophied. Scale bars, 2 mm. **e** Histological analysis of the ovaries, testis, and epididymis of the wild-type and FIN_{major}/FIN_{major} mice showed no germ cells in the homozygous mutants. Three adult mice (8 weeks of age) were used for each group. Scale bars, 100 µm. **f** Analysis of the number of PGCs in E8.5 $Trim37^{flox/-}$ and $Trim37^{cko}$ embryos by alkaline phosphatase staining. Arrows indicate TNAP-positive PGCs. Scale bars, 100 µm. **g** Whole-mount immunofluorescence staining of STELLA in E9.5 $Trim37^{flox/-}$ and $Trim37^{cko}$ embryos. Arrows indicate STELLA-positive PGCs. Scale bar, 100 µm.
