# Supplementary material for: TRIM37 interacts with TRIM28 to maintain primordial germ cell identity during migration
Source: Cell Res. 2026 Jul 30;36(10):785–803. doi: 10.1038/s41422-026-01272-2 (PMC13642344; doi:10.1038/s41422-026-01272-2)
Supplement: Supplementary file 19 [file 41422_2026_1272_MOESM7_ESM.pdf]

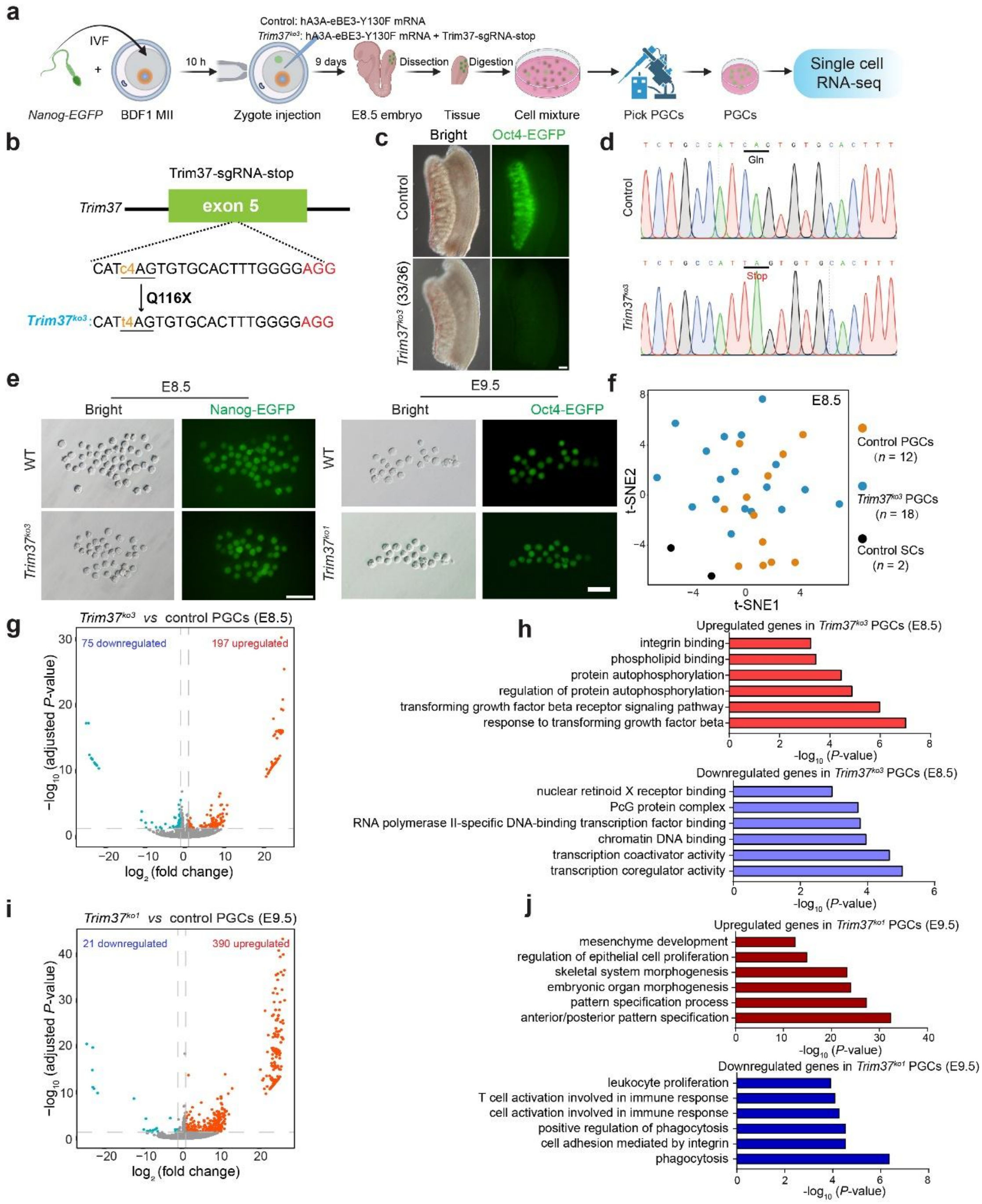

Fig. S7 The transcriptional profile of *Trim37* knockout PGCs at E8.5 and E9.5. a Schematic diagram of picking single PGCs from E8.5 embryos carrying the *Nanog-EGFP* transgene for RNA-seq. **b** The target sequence for the cytosine base editor in *Trim37* exon 5. The PAM sequence and the intended mutant bases are shown in red and yellow, respectively. The base editor can generate the stop code (p.Q116X) to inactivate *Trim37* (termed *Trim37*^{ko3}). **c** Images of gonads in E12.5 embryos obtained from control and *Trim37*^{ko3}. 33 out of 36 embryos obtained from this experiment were homozygotes. Scale bar, 200 μm. **d** Genotyping analysis of the control and *Trim37*^{ko3} embryos showing a base mutation in *Trim37*, leading to the replacement of Glutamine by a termination codon. **e** Images displaying PGCs collected from wild-type and *Trim37* deficiency embryos at E8.5 and E9.5 stages, respectively. E9.5 embryos carried the *Oct4-EGFP* transgene, while E8.5 embryos carried the *Nanog-EGFP* transgene. Scale bars, 100 μm. **f** t-SNE analysis and cluster allocation

for the RNA-seq data of single cells from wild-type and *Trim37*^{ko3} somatic cells (SCs) and PGCs at E8.5. *n* indicated the number of single cells. **g** Volcano plots show differentially expressed genes between *Trim37*^{ko3} and wild-type PGCs at E8.5 (adjusted *P*-value < 0.05, FC > 2). **h** Enriched GO terms of significantly upregulated and downregulated genes in *Trim37*^{ko3} PGCs compared to wild-type at E8.5. **i** Volcano plots show differentially expressed genes between *Trim37*^{ko1} and wild-type PGCs at E9.5 (adjusted *P*-value < 0.05, FC > 2). **j** Enriched GO terms of significantly upregulated and downregulated genes in *Trim37*^{ko1} PGCs compared to wild-type at E9.5.
