# Supplementary material for: TRIM37 interacts with TRIM28 to maintain primordial germ cell identity during migration
Source: Cell Res. 2026 Jul 30;36(10):785–803. doi: 10.1038/s41422-026-01272-2 (PMC13642344; doi:10.1038/s41422-026-01272-2)
Supplement: Supplementary file 20 [file 41422_2026_1272_MOESM8_ESM.pdf]

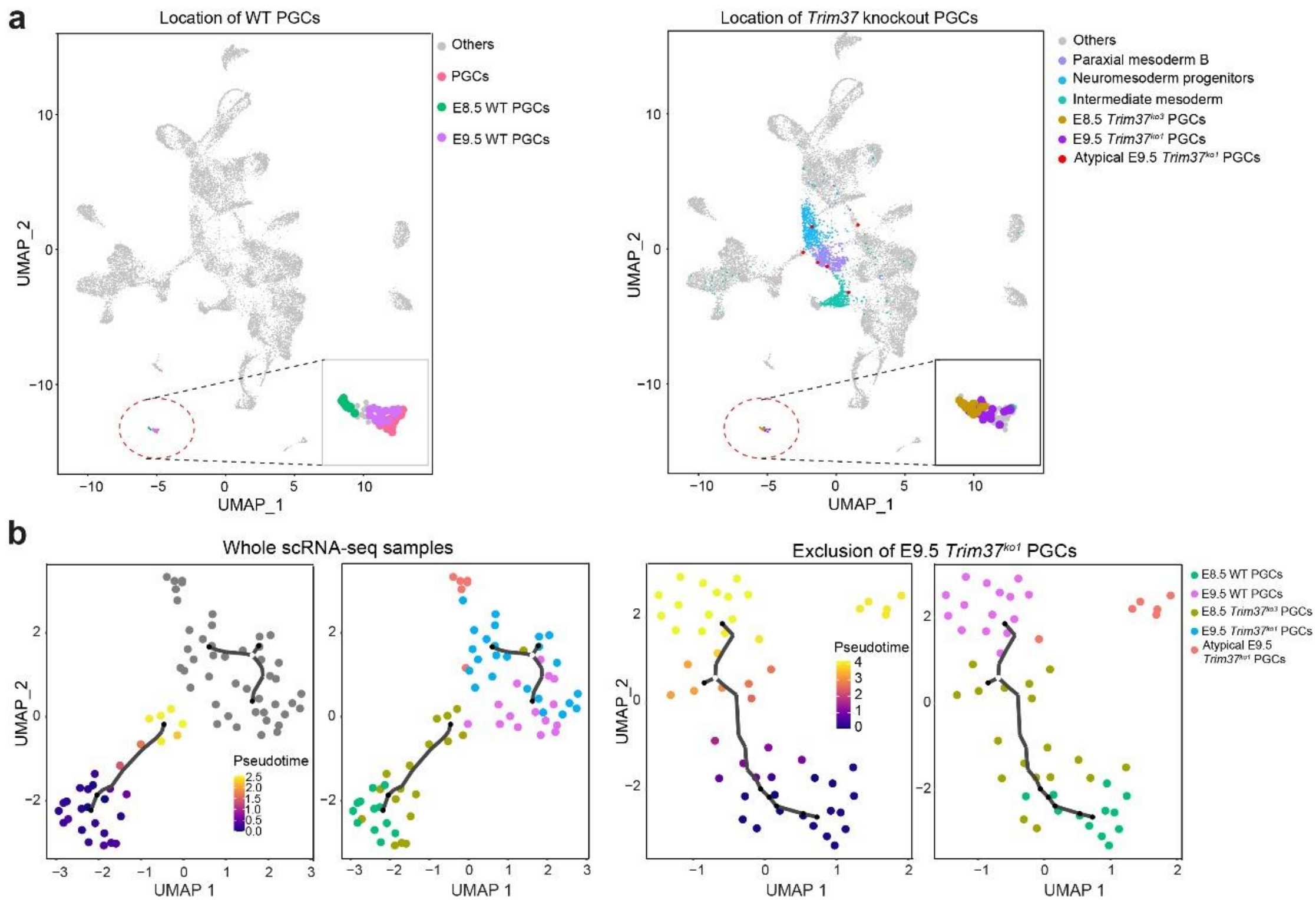

Fig. S8 Single-cell transcriptomic analysis reveals aberrant cell fate features in *Trim37*-deficient PGCs. a UMAP visualization of single-cell RNA-seq data. Left, integration of our E8.5 and E9.5 wild-type PGCs with published single-cell RNA-seq data from E9.5 embryos [34]. Right, UMAP showing E8.5 $Trim37^{ko3}$ and E9.5 $Trim37^{ko1}$ PGCs together with other embryonic germ layer–related cell types. Six atypical PGCs are separated from the main PGC cluster and positioned closer to mesoderm-associated lineages. **b** Pseudotime analysis of PGCs. Left, trajectory inference using all sequenced PGCs. Right, trajectory inference using E8.5 PGCs and atypical E9.5 $Trim37^{ko1}$ PGCs after excluding the normal E9.5 $Trim37^{ko1}$ PGC population, shown separately for clearer visualization of the inferred trajectory.
