# Supplementary material for: TRIM37 interacts with TRIM28 to maintain primordial germ cell identity during migration
Source: Cell Res. 2026 Jul 30;36(10):785–803. doi: 10.1038/s41422-026-01272-2 (PMC13642344; doi:10.1038/s41422-026-01272-2)
Supplement: Supplementary file 21 [file 41422_2026_1272_MOESM9_ESM.pdf]

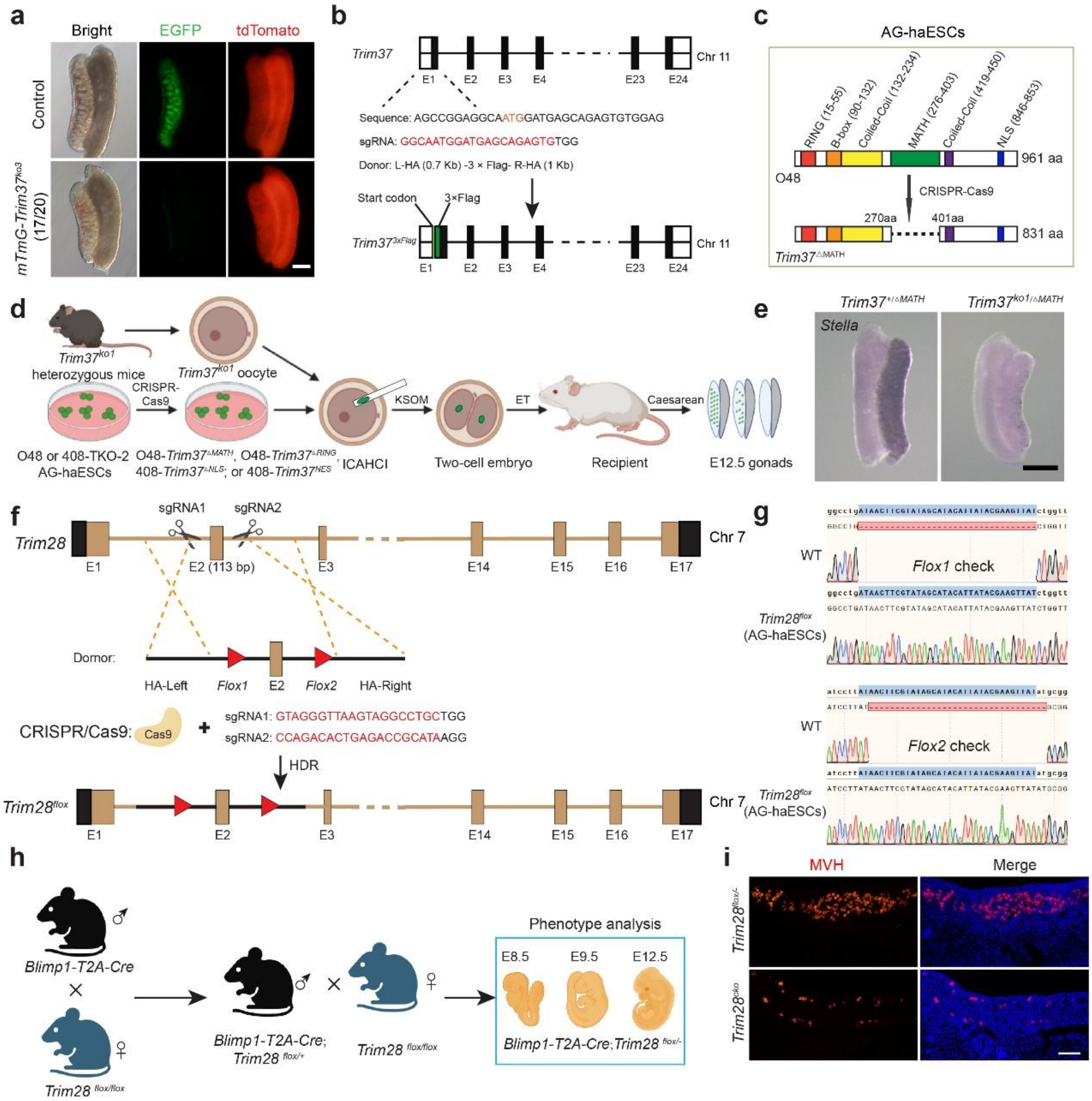

Fig. S9 The construction strategies and phenotypes of the *Trim37* deletion MATH and *Trim28* conditional knockout models. a Images of gonads in E12.5 embryos obtained from the lineage tracing system with or without *Trim37*-sgRNA-stop. The disappearance of GFP-positive PGCs in *mTmG-Trim37*^{ko3} gonads (17 out of 20 obtained embryos) demonstrated the efficiency of this lineage tracing system. Scale bar, 200 μm. **b** Schematic diagram of the construction of *Trim37*^{3xFlag} AG-haESCs by CRISPR-Cas9 mediated homologous recombination. The red bases represent the sgRNA sequence. **c** Schematic illustration of generating *Trim37*$^{\triangle MATH}$ AG-haESCs *via* CRISPR-Cas9 technology. **d** Experimental procedures for rapidly producing *Trim37* modified embryos through semi-cloning technology to analyze the phenotype of PGC development in one step. The *Trim37*-modified AG-haESC (*Trim37*$^{\triangle MATH}$, *Trim37*$^{\triangle RING}$, *Trim37*$^{\triangle NLS}$, and *Trim37*^{NES}) by CRISPR-Cas9 is injected into the *Trim37*^{ko1} oocyte to produce an embryo with a modification. **e** Analysis of PGCs in E12.5 *Trim37*$^{+/\triangle MATH}$ and *Trim37*$^{ko1/\triangle MATH}$ gonads by in situ hybridization of *Stella* probe, showing no PGCs in *Trim37*$^{ko1/\triangle MATH}$ gonads. Three embryos were used for each group. Scale bar, 200 μm. **f** Schematic diagram of the construction of *the Trim28*^{flox} mouse model by CRISPR-Cas9 mediated homologous recombination. The red bases represent the sgRNA sequence. **g** Sanger sequencing confirmed the accurate insertion of two *flox* sequences into the genome of the *Trim28*^{flox} AG-haESC line. **h** Strategy of generating *Trim28*^{cko} embryos using *Blimp1-T2A-Cre* and

Trim28$^{flox/flox}$ parents. **i** Immunofluorescence of E12.5 *Trim28*$^{flox/-}$ and *Trim28*^{cko} gonads evaluating MVH positive PGCs (red) and DAPI (blue). Three embryos were used for each group. Scale bar, 100 μm.
